# Association of men's exposure to family planning programming and reported discussion with partner and family planning use: The case of urban Senegal

**DOI:** 10.1371/journal.pone.0204049

**Published:** 2018-09-25

**Authors:** Ilene S. Speizer, Meghan Corroon, Lisa M. Calhoun, Abdou Gueye, David K. Guilkey

**Affiliations:** 1 University of North Carolina at Chapel Hill, Gillings School of Global Public Health, Department of Maternal and Child Health, Chapel Hill, NC, United States of America; 2 Carolina Population Center, University of North Carolina at Chapel Hill, Chapel Hill, NC, United States of America; 3 IntraHealth International, Dakar, Senegal; 4 University of North Carolina at Chapel Hill, Department of Economics, Chapel Hill, NC, United States of America; University of Michigan Medical School, UNITED STATES

## Abstract

**Background:**

Family planning programs increasingly aim to encourage men to be involved in women’s reproductive health decision-making as well as support men to be active agents of change for their own and the couple’s reproductive health needs. This study contributes to this area of work by examining men’s exposure to family planning (FP) program activities in urban Senegal and determining whether exposure is associated with reported FP use and discussion of family planning with female partners.

**Methods:**

This study uses data from two cross-sectional surveys of men in four urban sites of Senegal (Dakar, Pikine, Guédiawaye, Mbao). In 2011 and 2015, men ages 15–59 in a random sample of households from study clusters were approached and asked to participate in a survey about their fertility and family planning experiences. These data were used to determine the association between exposure to the Initiative Sénégalaise de Santé Urbaine (in English: Senegal Urban Reproductive Health Initiative) family planning program interventions with men’s reported modern family planning use and their reported discussion of FP with their partners. Since data come from the same study clusters at each time period, fixed effects methods at the cluster level allowed us to control for possible program targeting by geographic area.

**Results:**

Multivariate models demonstrate that religious leaders speaking favorably about family planning, seeing FP messages on the television, hearing FP messages on the radio, and exposure to community outreach activities with a FP focus (e.g., house to house and community religious dialogues) are associated with reported modern family planning use and discussion of family planning with partners among men in the four urban sites of Senegal.

**Conclusions:**

This study demonstrates that it is possible to reach men with FP program activities in urban Senegal and that these activities are positively associated with reported FP behaviors.

## Introduction

Prior to the 1994 International Conference on Population and Development (ICPD), family planning (FP) efforts almost exclusively focused on delivery of clinical services to women [1, 2]. Following the 1994 ICPD meeting, there was increasing attention to engaging men as partners to increase couple communication and encourage men’s support for women’s FP decision-making [3]. Engaging men is particularly important given that husbands and male partner opposition are often given as reasons for contraceptive non-use by women [4–7]. Programs that involve men can increase spousal communication frequency, address gender inequitable norms, and lead to greater FP use [8].

The evidence on male targeted FP programs has evolved over time. Several systematic reviews have examined programs targeting men to identify effective strategies for engaging men as partners and strategies to improve men’s own reproductive health needs [3, 9, 10]. One earlier review of evaluations of men’s involvement in sexual and reproductive health programs demonstrated that 10 years after the 1994 ICPD conference there was still little programmatic engagement of men and few evaluations of interventions engaging men in sexual and reproductive health programs [10]. The studies at the time of the review demonstrated that men’s involvement was related to men’s positive support for women’s contraceptive use and that men were not necessarily a barrier to use [10]. A more recent review of evidence of changing gender norms among men to improve reproductive health outcomes demonstrates that programs that use a gender-transformative approach (i.e., promote gender-equitable relationships between women and men) and those that include multiple components were the most successful [9]. Finally, Hardee and colleagues [3] recently reviewed 47 interventions that reached men as users/clients (for condoms, vasectomy, withdrawal, and Standard Days Method) to provide recommendations to strengthen FP programming for men. Notably, an identified gap in the review was the need for more robust evaluations of programs that target men [3]. Among the programs identified, those that were considered “proven” (i.e., strong evidence) included social marketing and outreach with male motivators/peer educators [3]. Social marketing generally increases men’s access to contraceptives whereas outreach by male motivators can improve men’s knowledge and attitudes, address community norms around FP use, as well as increase access to methods [11]. Promising activities included community dialogue (i.e., community engagement), mass and social media, and clinic-level provision of information and services. Community dialogue and mass/social media can address men’s knowledge and attitudes as well as community norms, whereas, clinic-level activities generally focus on access to methods. Finally, emerging areas for reaching men included mobile health interventions (mHealth), hotlines, and engaging religious leaders [3]. The Malawi Male Motivator project is an example of a peer-led program that used a randomized evaluation design to demonstrate that engaging men and promoting couple communication lead to greater reported family planning uptake among men [12]. In addition, spousal discussion of family planning has been found to be significantly related to male engagement and contraceptive uptake in varying contexts [13–20].

Recent qualitative studies from Nigeria and Togo demonstrate that men want and expect to be part of the decision-making process about family size and childbearing [21, 22]. That said, while the Togo study demonstrated common misperceptions around FP among men, there were also clear socioeconomic motivations that led men to consider (or use) FP [22]. In both Nigeria and Togo, a key barrier to men’s involvement in FP was the common thinking among the male participants that FP is the woman’s domain [21, 22]. The authors of these West African studies promote the need for community engagement strategies that reach men and couples to address myths and misperceptions and improve couple communication about FP [21, 22].

In Senegal, the site of this study, between 2012 and 2015, significant increases were observed in modern contraceptive use from 12% of married women to 21% of married women [23, 24]. This impressive increase fell below the government’s commitment made at the 2012 London Summit on Family Planning which was to achieve a modern contraceptive prevalence rate of 27% by 2015 [23]. A key program that supported Senegal’s increase in FP use between 2012 and 2015 was the Initiative Sénégalaise de Santé Urbaine (ISSU) or Senegal Urban Reproductive Health Initiative, launched in 2010 with funding from the Bill & Melinda Gates Foundation (BMGF). The ISSU project, with government support and engagement, undertook a multi-component program that included improving the quality and availability of contraceptive services by trained providers, integrating service delivery, and developing a reliable contraceptive supply system to reduce stockout of methods. The program also undertook a number of activities to increase demand for modern FP including mass media campaigns on the radio and television; community outreach activities that included one-on-one interactions at a person’s home and community drama productions; and engaging religious leaders to speak favorably about FP in their sermons as well as part of a radio series.

Notably, the Senegal ISSU program examined here used many of the proven, promising, and emerging program approaches for engaging men [3] with the goal of increasing modern contraceptive use in six cities and particularly among the urban poor. In a separate impact evaluation, women exposed to ISSU-led community outreach activities were significantly more likely to report using modern contraception at endline than women not exposed to community outreach [25]. None of the other ISSU program activities were found to be related to women’s reported use over time. All of the ISSU mass media activities reached men as well as women; some of the messages were targeted to men as key gatekeepers of FP within the household. In addition, community-based activities that involved drama on couple communication and community-based activities with religious leaders also sought to engage men and encourage couple communication. This paper examines the associations between ISSU programming and men’s reported modern contraceptive use and reported spousal discussion of FP in the ISSU study sites. We hypothesize that we will find a positive association between men’s exposure to the ISSU program activities and their reported modern family planning use and discussion of family planning with their partner.

## Materials and methods

The data for this study come from baseline (2011) and endline (2015) surveys collected as part of the evaluation of the ISSU program. The Measurement, Learning & Evaluation (MLE) project at the University of North Carolina at Chapel Hill was responsible for evaluating the ISSU project and sister projects in Kenya, Nigeria, and the state of Uttar Pradesh, India. In Senegal, data from men were collected from four urban sites that are part of the wider region of Dakar: Dakar, Guédiawaye, Pikine, and Mbao. At baseline, a two-stage sampling design was used to obtain a representative sample of households and men. At baseline, we used the 2009 updated version of the 2002 General Population and Housing Census list of census districts (also called clusters) which served as our study primary sampling units–PSUs. In each city, a random sample of clusters was selected in the first stage with 64 clusters selected in Dakar and 32 in each of the smaller sites (Guédiawaye, Pikine, Mbao); the number of clusters selected was reflective of the census population size estimates for the sites. In each selected cluster, a full household listing and mapping was conducted. Following the listing and mapping, 11 households were randomly selected for the men’s interview in each cluster with equal probability of selection; more details on the study design can be found in the baseline report [26]. At endline in 2015, we returned to the same clusters as baseline but a new listing and mapping exercise was undertaken and 11 households were again randomly selected for interview.

At each round of data collection, in each selected household in the four sites, all men ages 15–59 were eligible for interview. All eligible men were approached by a trained male interviewer and asked for their signed consent to be interviewed. For this analysis, we pooled the data from the two rounds of data collection to permit making comparisons between the baseline and endline cross-sectional samples. This analysis examines two dependent variables. The first dependent variable is reported use of modern contraception. At baseline and endline, men were asked if they (or their partner) were using a contraceptive method to delay or avoid childbearing and those who reported yes were asked what method they used. Modern methods of contraception include male and female sterilization, daily pill, intrauterine device (IUD), implants, injectables, male and female condoms, emergency contraception, Standard Days Method, and lactational amenorrhea; these last two methods are coded as modern, in accordance with the Senegal Demographic and Health Survey [24]. Men who reported traditional method use (e.g., rhythm method, withdrawal, or folkloric methods) were coded as non-modern method users. The second dependent variable is specifically focused on men who were in union (married or living with a partner). Men who were in union at the time of interview were eligible to be asked about whether (and when) they discussed FP with their partner. Those men in union who reported that they discussed FP in the last six months were coded one and all others were coded zero.

This analysis examines the association between exposure to various ISSU program activities and the outcomes of interest. Table 1 presents the description of the program exposure variables as measured in the survey as well as the percentage of men exposed to each program element at baseline and endline. Because the ISSU program activities had not begun before baseline data collection, ISSU-specific variables were coded as zero at baseline (e.g., exposure to ISSU community religious talks, ISSU community activities, and ISSU radio or television). Two types of radio exposure variables are included in the model: exposure to any FP message on the radio and exposure to ISSU specific messages on the radio. Likewise, two types of television exposure variables are included in the analysis: general FP television exposure and ISSU specific exposure. For both radio and the television, we asked about specific shows and stations where the ISSU program was aired to more specifically measure ISSU media exposure. Most of the men who were exposed to the ISSU radio or television also reported general radio and television exposure. Numerous community-level exposure variables were measured as part of the evaluation and these were specifically related to activities undertaken by the ISSU program. These included a) participating in a community-based religious talk on FP; b) hearing a religious leader speak favorably about FP (asked at baseline and endline); and c) participating in outreach activities (community activities). The community activities measured were those that ISSU implemented in the study cities and included community meetings on FP, community conversations about FP, small group discussions (niche) on FP, and an outreach worker visiting the home to discuss FP. Notably, outreach workers were generally targeting women but the men were not excluded from participation.

**Table 1:** Description of program exposure measures and percentage of men who ever had sex who report being exposed to each program exposure activity at baseline (2011) and endline (2015) in Senegal, MLE.

FP Exposure Measure	Exposure Components	Baseline* (n = 1,579)	Endline* (n = 1,524)
Newspaper/magazine	• Read about FP in a newspaper or magazine in the last three months	15.30	20.10
General radio	• Heard FP message on the radio in the last three months	42.86	79.87
	• Heard an FP spot/advertisement on the radio in the last year		
ISSU radio message	• Heard FP on a health radio program in last year^1^	0	19.47
	• Heard FP on a religious radio program in last year^2^		
	• Heard FP on a musical radio program in last year^3^		
	• Heard FP on an interactive radio program in last year ^4^		
	• Heard FP on the radio program Xam sa yaram in last year^5^		
General TV	• Seen an FP message on the TV in the last three months	58.79	87.75
	• Seen an FP spot/advertisement on the TV in the last year		
ISSU TV^6^ message	• Heard FP on Dine Ak Diamano on Walf TV in last year	0	60.96
	• Heard FP on Ndieguemar on Télévision Futurs Média (TFM) in last year		
	• Heard FP on Li ci penc mi on TFM in last year		
	• Heard FP on Thow li Thiow li on 2STV in last year		
	• Heard FP on Sen DINE on la SEN TV in last year		
	• Heard FP on Decriptage on la 2STV in last year		
	• Heard FP on Xam sa yaram on Lamp FALL TV in last year		
ISSU community religious talks	• Participated in the last year in a community-based religious talk	0	14.05
Religious leader	• Heard a religious leader speak favorably about FP in the last year	18.25	47.41
ISSU community activities	• Heard about FP at a community meeting in the last year	0	11.54
	• Heard about FP at a community conversation in the last year		
	• Heard about FP at a niche anime in the last year		
	• Heard about FP at a home visit by outreach worker in the last year		

^1^ On the following radio stations: Rail Bi FM, Oxyjeunes, Afia FM, Mozdair, Jokko rufisque, dounya, Sud FM, Renndo, Lamp fall, Mbour FM or Alfayda.

^2^ On the following radio stations: Rail Bi FM, Oxyjeunes, Afia FM, Jokko rufisque, dounya, Sud FM KL, Renndo, Lamp fall, Mbour FM or Alfayda.

^3^ On the following radio stations: Rail Bi FM, Oxyjeunes, Afia FM, Jokko rufisque, dounya, Sud FM KL, Afia FM, Jokko rufisque, dounya, Sud FM KL

^4^ On the following radio stations: Loci Xam sur Sud FM.

^5^ On the radio stations Lamp Fall FM.

^6^ Exposure to all ISSU TV programs was measured in the last year.

* Weighted percentages and numbers; Note: all differences between baseline and endline have a p-value less than 0.05 on the Pearson F-test comparison.

Given that we interviewed men from the same communities at baseline and endline, we use fixed effect methods to control for possible program geographic targeting. For example, the effect of community outreach programs could be biased if these programs were targeted to communities based on unmeasured characteristics of the communities. Our methods correct for this source of community level bias. However, they do not correct for individual recall bias to the exposures which is why our analysis is one of association rather than causality.

All descriptive analyses use weights from the corresponding baseline or endline survey. To compare the two time periods, we present p-values from Pearson F-tests performed in Stata statistical software version 14.1. For multivariate analyses, we present the coefficients and standard errors from the fixed effect regression models and focus on the sign and significance of the results. All analyses adjust for clustering based on the survey design using Huber-White type sandwich estimators for standard errors. All study procedures were approved by the Institutional Review Board at the University of North Carolina at Chapel Hill as well as the Comité National d’Ethique pour la Recherche en Santé through the Ministry of Health in Senegal.

## Results

At baseline, 2,270 men were successfully interviewed across the four cities. At endline, a total of 2,214 men were interviewed in the same cities and same survey clusters. For the analysis of men’s modern contraceptive use, we focus on the men who reported that they had ever had sex, which reduces the baseline sample to 1,491 (66% of the full sample) and the endline sample to 1,490 (67% of the full sample). Most of the men dropped from the analysis sample have never been married. At baseline, 51% of the never married men had never had sex and at endline, 57% of the never married men had never had sex. To examine spousal communication about FP, we focus on the sub-sample of men who ever had sex and were in union at the time of the baseline (n = 833) or endline (n = 978) surveys. Men who were not in union were not asked the questions about partner communication and therefore were dropped from the analysis of this outcome.

Table 2 presents the demographic characteristics of the cross-sectional analysis sample of men who had ever had sex at baseline and endline. Table 2 demonstrates that the endline sample is somewhat older than the baseline sample; this corresponds to a greater percentage of the endline sample being in union and the endline sample having higher parity compared to the baseline sample that includes more men without children and who have never been married. In the four urban sites, a quarter of men have no education or only Quranic education; another 30% have only a primary education level. A quarter of men at both time periods have secondary or higher education levels. As expected, most of the sample is Muslim (nearly 90%).

**Table 2:** Percentage of men who ever had sex by demographic characteristics among men from four urban sites in Senegal at baseline (2011) and endline (2015), MLE.

	Baseline Analysis†	Endline Analysis†
Characteristics	(n = 1,491)	(n = 1,490)
**Age:** 15–19	4.51	1.42
20–24	13.76	10.26
25–29	19.22	14.14
30–34	16.27	20.95
35–39	16.75	18.16
40–44	10.07	11.64
45–59	19.43	23.43***
**Education:** No education/Quranic	22.39	23.20
Primary	29.27	29.92
Secondary incomplete	21.69	19.49
Secondary complete	14.25	11.81
Higher	12.40	15.59
**Religion:** Christian	11.31	10.39
Muslim	88.60	89.52
None/other/missing	0.09	0.09
**Wealth:** Poorest	23.81	25.67
Poor	21.28	21.26
Middle	18.97	17.58
Rich	17.34	18.33
Richest	18.60	17.17
**Marital status:** Never married	42.61	33.62
In union (at time of interview)	54.23	63.54
Divorced, widowed, separated, missing	3.93	2.84**
**City:** Dakar	53.16	52.54
Guédiawaye	11.82	9.59
Pikine	13.25	11.62
Mbao	21.77	26.25
**Parity:** Zero	46.01	37.23
1	11.90	16.63
2	10.88	12.29
3	7.72	11.38
4	7.78	6.18
5	4.78	5.18
6+	10.93	11.11**

†Each survey uses the weights from that sample. Unweighted number of observations shown; weighted n’s are 1,579 at baseline and 1,524 at endline

** p≤ 0.01

*** p≤ 0.001, from Pearson F-test comparison.

Table 3 presents descriptive statistics for the outcomes at baseline and endline. First, examining contraceptive use, at baseline 40% of men who ever had sex report that they or their partner is using a modern method and 5% report using a traditional method. By endline, the percentage reporting modern method use has dropped slightly to 37% and traditional method use remains about the same; this difference is not significant. While use did not change significantly between baseline and endline, the reported method mix did change significantly towards a larger share of men reporting use of long-acting methods. At endline, a greater percentage of men report that their partner is using implants and injectables compared to baseline. This represents a decline in use of less effective methods such as male condoms and pills. Also presented in Table 3 is the answer to a question about discussion of family planning among men in union. We see that recent reported discussion of family planning (in the last 6 months) declined somewhat, although the difference is not significant. Multivariate analyses presented in Tables 4 and 5 control for the observed differences in demographic factors (e.g., age, marital status and parity) between the samples to better inform the differences in the outcomes over time.

**Table 3:** Men’s reported contraceptive use with their partner, method mix, and discussion of family planning with spouse at each time period among men who ever had sex in four cities at baseline (2010) and endline (2015), Senegal, MLE.

	Baseline	Endline
**Reported Contraceptive Use with Partner**	(n = 1,579)	(n = 1,524)
Modern	39.84	36.82
Traditional	5.23	5.77
Non-user	54.93	57.41
**Reported Method mix (among method users)**	(n = 711)	(n = 649)
Sterilization	0.44	1.54
Implants	3.29	5.94
IUD	1.46	2.34
Injectables	6.22	15.45
Pill	14.53	10.69
Male condom	61.67	48.84
Other modern methods†	0.47	1.56
Traditional method	11.61	13.55***
**Among men in union (%)**	(n = 856)	(n = 968)
Discussed FP with partner in last 6 months	39.50	35.98

†Other modern methods include lactational amenorrhea, emergency contraception, female condom, spermicides, and Standard Days Method (endline only). Note: All results in this table are weighted

*** p≤ 0.001, from Pearson F-test comparison.

**Table 4:** Multivariate fixed effect regression coefficients and standard errors for the association between program activities on men's reported modern method use among men who ever had sex in four cities of Senegal, MLE, n = 2,981.

	β (SE)
**Newspaper/magazine**	**-0.03 (0.03)**
**General radio exposure**	**0.03 (0.02)**
**ISSU radio exposure**	**-0.01 (0.04)**
**General television exposure**	0.10 (0.02)***
**ISSU television exposure**	**-0.01 (0.03)**
**ISSU community religious talks**	0.10 (0.04)*
**Religious leader**	0.09 (0.02)***
**ISSU community activities**	0.10 (0.04)*
Endline (baseline—ref)	-0.09 (0.03)**
Parity: (no children—ref)	ref
1 child	0.09 (0.03)**
2 children	0.15 (0.04)***
3 children	0.21 (0.04)***
4 children	0.20 (0.04)***
5 children	0.22 (0.05)***
6+ children	0.19 (0.04)***
Marital status (single—ref)	ref
In union	-0.28 (0.03)***
Divorced/widowed/separated	-0.33 (0.05)***
Age group (15–24—ref)	ref
25–29	0.07 (0.03)*
30–34	0.11 (0.03)***
35–39	0.16 (0.03)***
40–44	0.04 (0.03)
45–59	0.05 (0.03)
Muslim (vs. Christian/other)	-0.08 (0.04)*
Education (no education/ Quranic—ref)	ref
Primary	0.16 (0.02)***
Secondary incomplete	0.16 (0.03)***
Secondary complete	0.20 (0.03)***
Higher	0.21 (0.03)***
Wealth group (poorest—ref)	ref
Poor	0.03 (0.03)
Middle	0.03 (0.03)
Rich	0.03 (0.03)
Richest	0.08 (0.03)*

Note: Fixed effects models of cross-sectional male samples interviewed in 2011 and 2015 from the same clusters. Overall model p-value from F-test is less than 0.001; this indicates that the model is significantly different than a model with just a constant term.

+ p≤ 0.1

* p≤ 0.05

** p≤ 0.01

*** p≤ 0.001.

**Table 5:** Multivariate fixed effect regression coefficients and standard errors for the association between program activities on men's discussion of family planning in the last 6 months among men in union in four cities of Senegal, MLE, n = 1,811.

	Discussed FP in last 6 months β (SE)
**Newspaper/magazine**	**0.05 (0.04)**
**General radio exposure**	0.06 (0.03)*
**ISSU radio exposure**	0.09 (0.04)*
**General television exposure**	**0.07 (0.03)+**
**ISSU television exposure**	**0.02 (0.04)**
**ISSU community religious talks**	**-0.03 (0.04)**
**Religious leader**	0.12 (0.03)***
**ISSU community activities**	0.11 (0.05)*
Endline (baseline—ref)	-0.16 (0.03)***
Parity: (no children—ref)	Ref
1 child	0.10 (0.04)**
2 children	0.18 (0.04)***
3 children	0.24 (0.04)***
4 children	0.14 (0.05)**
5 children	0.27 (0.05)***
6+ children	0.18 (0.04)***
Age group (15–24—ref)	Ref
25–29	0.20 (0.05)***
30–34	0.13 (0.04)**
35–39	0.12 (0.04)**
40–44	0.08 (0.04)*
45–59	-0.02 (0.04)
Muslim (vs. Christian/other)	-0.01 (0.05)
Education (no education/ Quranic—ref)	Ref
Primary	0.08 (0.03)*
Secondary incomplete	0.16 (0.04)***
Secondary complete	0.14 (0.04)***
Higher	0.17 (0.05)***
Wealth group (poorest—ref)	Ref
Poor	0.14 (0.03)***
Middle	0.07 (0.04)+
Rich	0.06 (0.04)+
Richest	0.14 (0.04)**

Note: Fixed effects models of cross-sectional male samples interviewed in 2011 and 2015 from the same clusters.

Overall model p-value from F-test is less than 0.001; this indicates that the model is significantly different than a model with just a constant term.

+ p≤ 0.1

* p≤ 0.05

** p≤ 0.01

*** p≤ 0.001.

Table 1, presented earlier, provides a description of the program exposure variables including ISSU specific and general FP exposure. Also presented in Table 1 is the percentage of men exposed to each of the activities at baseline (if applicable) and endline. At baseline, about 15% of men reported that they had read about FP in the newspaper or in a magazine in the last three months; by endline this percentage had increased somewhat to 20% (p = 0.04). Exposure to FP messages on the radio increased significantly over time from 43% of men reporting exposure at baseline to 80% at endline. Some of the increase in exposure to FP messages on the radio is contributed by ISSU specific messages and programming; by endline, about 20% of men had heard the ISSU specific radio programming. FP is also being presented on the television such that at baseline 59% of men reported television exposure to FP and by endline, this had significantly increased to 88%. At endline, a high percentage of men (61%) had seen an ISSU specific television program that covered FP. A question was asked at baseline and endline about exposure to a religious leader speaking favorably about FP. At baseline, nearly 18% of men reported exposure to positive messages from religious leaders and by endline this had more than doubled to 47%. While this was not an ISSU specific question, working with religious leaders on FP messaging was a key activity mainly implemented in the four cities by the ISSU program. In addition, 14% of men reported participating in community-based religious talks at endline and 12% were exposed to another type of community-based activity with a FP theme; most of these activities are ISSU-specific community-based activities.

Table 4 presents the multivariate fixed effect regression coefficients and standard errors from the model examining the association between ISSU program exposure and men’s reported modern method use among men who had ever had sex. In the full model, the endline dummy variable, coded 1 in 2015, is negative and significant; this is consistent with the decline in CPR seen in the descriptive results. Table 4 demonstrates that controlling for the demographic factors and survey period, there are a number of program factors associated with men’s reported modern method use. Men who were exposed to FP messages on the television (p≤0.001), men who heard a religious leader speak favorable about FP (p≤0.001), men who heard community-level religious talks on FP (p≤0.05), and men who were exposed to ISSU community-level activities (p≤0.05) were significantly more likely to be using a modern method than men who were not exposed to these activities. While a number of these exposure effects are not specific to the program, controlling for general exposure (e.g., radio and television) still results in significant associations between the ISSU community and religious-based activities on modern contraceptive use. The control variables in this model are all in the expected direction: men with more children were more likely to use FP and men in the prime reproductive years (ages 25–35) were more likely to use than men ages 15–24. Men who were in union and men who were divorced, widowed or separated were less likely to be modern method users than sexually experienced men who had never been in union. Men who were Muslim were less likely to use than men who were Christian. In addition, men who were more educated were significantly more likely to use than men with no education or Quranic education only.

Table 5 presents the association between program exposure and the discussion outcome in the sample of men in union. Those men exposed to religious leaders speaking favorably about FP were significantly more likely to report recently discussing family planning with their spouse (p≤0.001) than those men who did not hear a religious leader speaking favorably about FP. Further, radio exposure, both general and ISSU specific, was associated with greater recent discussion. Exposure to community-based activities was positively associated with reported recent family planning discussion. Finally, general television exposure was positively associated with reported discussion of FP in the last six months. The control variables show expected results. The time variable (endline vs. baseline) is negative and significant indicating that after controlling for exposure and the demographic factors, reported discussion declined. Further, those men with more children were more likely to report discussion of FP. Those in the reproductive years were more likely to report discussion. More educated men reported more recent discussion than men with no education or Quranic only education and richer men reported more discussion of FP than the poorest men.

## Discussion

Senegal needs to identify ways to continue positive trends in contraceptive use to attain its FP goals and commitments. Including men in the FP equation as potential users or supporters of FP is an important step for meeting these goals as men can be a barrier (perceived or real) to couples’ use [9, 27]. Programs targeting men are needed to address men’s desire and expectation to be part of the decision-making process about family size and childbearing and to shift social norms around FP use [21, 22]. This study demonstrates which of the ISSU program components were associated with reported FP use and discussion of FP among men. In particular, men who were exposed to a religious leader speaking favorably about FP were more likely to report using FP and discussing FP with their spouses. Further, radio activities (both ISSU specific and general programming on FP) were associated with FP discussion and television exposure (general) was associated with FP use. Finally, there was an association between community-based activities and these outcomes.

Interestingly, in the evaluation that examined the impact of the ISSU program on women’s modern method use, only community-based activities were found to be significant [25]. There were no identified effects of radio, television or religious leaders on women’s likelihood to use modern contraception. This is in contrast to the findings here that show that these other activities are associated with men’s reported FP use and spousal discussion. The observed associations found here may reflect the program activities that are important for men and potentially, these may influence women’s choices indirectly. As discussed earlier, men do play a role in decision-making in the region either directly or indirectly and thus, should also be engaged in FP programming in urban Senegal [21, 22]. Programs should consider tailored interventions for men separately from women (and to examine outcomes among both) since exposure and effects may differ by sex [3, 21, 22]. Thus, as part of developing program strategies, it is worth considering the direct and indirect effects of program activities on both women and men.

Earlier studies from urban West Africa show that program exposure is related to positive family planning outcomes among men. In particular, one study that used data collected from men in two cities in Nigeria (Kaduna and Ibadan) demonstrated that men who were exposed to the Nigerian Urban Reproductive Health Initiative (NURHI) media campaign had significantly greater contraceptive use ideation [28]. In the analysis, contraceptive use ideation was a summary measure capturing contraceptive awareness, myths and rumors, approval of government officials discussing FP, perceived self-efficacy to use family planning, spousal discussion of FP, and men’s approval of FP [28]. Further, a recent analysis of men from urban areas of Kenya, Nigeria and Senegal demonstrated which programmatic factors were associated with men’s reported contraceptive use [29]. The authors showed that among men in Kenya, participation in community events and exposure to television programs related to FP were associated with modern contraceptive use [29]. Further, among men in Nigeria, the only program activity that was significantly associated with modern method use was exposure to program slogans in English (i.e., branding) [29]. Finally, the analysis also included men from three cities in Senegal and examined ISSU program activities based on data from midterm in 2013 (the current paper uses data from endline in 2015). The earlier analysis among men in Senegal showed that exposure to program-led radio and television programming and exposure to religious leaders speaking in favor of FP were associated with modern method use [29]. The results presented in this paper are consistent with these earlier findings, however, by endline there were also significant associations between men’s exposure to community-level activities (community dialogues by religious leaders and community outreach) and men’s reported contraceptive use and discussion of FP with their partner. These community-level activities that are interpersonal in nature may take longer to attain a large enough coverage to see associations; the four-year follow-up may have provided the longer time period necessary for associations to be observed.

This study has a few limitations that need to be acknowledged. First, the study sample represents two separate cross-sections from the same sample clusters in the four cities. While we can use the clusters in the fixed effect analyses to control for possible program targeting at the cluster level, the results of the analyses are simply associations. We cannot show causal relationships between program activities and the outcomes of interest. Second, overall, we observe slight declines in the outcomes in the endline sample; this may reflect unobserved sample differences between baseline and endline but may also reflect true declines among men. In models that did not include the program variables, this negative time effect was attenuated or disappeared. In the women’s longitudinal sample, reported modern method use increased by five percentage points [25]. Among the longitudinal sample of women, this may reflect life course factors that increase the need for FP with older age and continued childbearing. That said, repeated cross-sectional Demographic and Health Survey (DHS) data from Senegal suggests increases in contraceptive use over time as reported by women [24]. Notably, the 2015 DHS data from Senegal demonstrates that more women in union report modern method use (21%) than men in union (15%) and among users, only 4% of women in union report condom use whereas 18% of men in union who report using a modern method report condom use (author calculations). Therefore, it is possible that men are mis-reporting their use, men report less condom use as a FP method over time, men do not know about increases in long-acting method use among their partners, or there are true declines in use across the two cross-sectional samples. With the data available, we cannot determine which of these scenarios is the correct one. Another limitation of this analysis is that the general television and radio exposure variables that increase over time are picking up increases in ISSU programming but may also be picking up increases in other mass media programming taking place in Senegal; with the data available, it is not possible to make this distinction fully. Finally, there is some collinearity between the survey wave (time) and the variables that are only measured at endline; this is a consequence of the program not existing at baseline.

## Conclusions

This study takes a first step to examine which types of program activities are associated with changes in men’s reported FP behaviors and communication. Working with religious leaders, which was identified as an emerging strategy [3], was associated with modern FP use and spousal communication in the urban Senegal context. Further, we demonstrated that community-based activities and radio and television programs can lead to high exposure to FP messages among men. Identifying the best combination of mass media and interpersonal activities to increase men’s engagement in FP is an important next step for programs seeking to meet men’s own FP needs as well as those of their partners. The findings from this study were used by the ISSU program to strengthen their programming and can also be used to inform future programming in urban Senegal and in other parts of urban francophone Africa.

## Supporting information

S1 Baseline Men’s Questionnaire (PDF)

S1 Endline Men’s Questionnaire (PDF)

S1 Supporting Information (DOCX)
