# Supplementary material for: Association of men's exposure to family planning programming and reported discussion with partner and family planning use: The case of urban Senegal
Source: PLoS One. 2018 Sep 25;13(9):e0204049. doi: 10.1371/journal.pone.0204049 (PMC6155530; doi:10.1371/journal.pone.0204049)
Supplement: S1 Baseline Men’s Questionnaire — (PDF) [file pone.0204049.s001.pdf]

S	GR	N°MEN	N°LIGN

MESURE APPRENTISSAGE ET EVALUATION (MLE) DE L'INITIATIVE SENEGALAISE DE SANTE URBAINE (ISSU)
ENQUETE DE BASE (2011)

QUESTIONNAIRE HOMME

S	GR	N°MEN	N°LIGN

République du Sénégal

MINISTERE DE LA SANTE ET DE LA PREVENTION

CRDH

IDENTIFICATION

SITE ______	SITE………………………
(DAKAR=1, GUEDIAWAYE=2, PIKINE=3, MBAO=4, MBOUR=5, KAOLACK=6)	
DISTRICT SANITAIRE……………………………………………	DS……………………
NOM DU QUARTIER ______	
NUMÉRO DE GRAPPE………………………………………………..	GRAPPE……
STATUT DE LA GRAPPE (PAUVRE=1, NON PAUVRE=0)…………………………..	STATUT……………………
NUMÉRO DE CONCESSION……………………………………………	NO CONS…….
NUMÉRO DE MÉNAGE……………………………………………	N^O MÉN……….
NOM ET NUMÉRO DE LIGNE DE L'HOMME ______	N^O LIGNE……………

VISITES POUR L'INTERVIEW

	1	2	3	VISITE FINALE
DATE	______	______	______	JOUR . . . .
				MOIS . . . .
RÉSULTAT*	☐	☐	☐	ANNÉE 2 0 1 1
NOM DE L'ENQUÊTRICE	______	______	______	CODE RÉSULTAT…
CODE	______	______	______	CODE ENQ…
PROCHAINE VISITE DATE	______	______		NBRE TOTAL DE VISITES……………
HEURE	______	______		

*CODES RÉSULTAT :

01	REMPLI	04	REFUSÉ		
02	PAS À LA MAISON	05	REMPLI PARTIELLEMENT	96	AUTRE ______
03	DIFFÉRÉ	06	INCAPACITÉ		(PRÉCISER)

LANGUE DE QUESTIONNAIRE** ______ 1

LANGUE DE L'INTERVIEW** ______

RECOURS A UN INTERPRÈTE : (OUI=1, NON=2)

**CODES LANGUE :

01 FRANÇAIS	04 SERER	96 AUTRES______ (PRECISER)
02 WOLOF	05 DIOLA	
03 POULAR	06 MANDINGUE	

SUPERVISEUR	CHEF D'ÉQUIPE	CONTRÔLE BUREAU	SAISI PAR :
NOM ______	NOM ______	NOM ______	NOM ______
CODE…………	CODE…………	CODE…….	CODE..
DATE ______	DATE ______	DATE ______	DATE ______

S		GR		N°MEN		N°LIGN

Lettre d'information de l'homme dans l'enquête ménage

Nom et prénoms de l'homme : ____

Monsieur,

Mon nom est_______________________ et je travaille comme enquêteur/chef d'équipe dans une étude commanditée par Intrahealth pour le compte du Ministère de la Santé et de la Prévention et réalisée par le CRDH (Centre de Recherche pour le Développement Humain).

Il vous est proposé de participer à une enquête sur la santé maternelle et infantile qui vise à améliorer la qualité de vie des populations urbaines des villes sénégalaises à travers l'augmentation de l'accès et l'utilisation des services de santé de la reproduction. Si vous acceptez de participer, vous ferez partie des 14 000 personnes qui seront interrogées dans le cadre de cette étude Les informations que vous allez nous fournir seront très utiles au gouvernement pour planifier les politiques de santé et répondre aux besoins non satisfaits en santé de la reproduction. Votre localité a été choisie au hasard parmi d'autres de votre région.

Vous serez interviewé par une enquêtrice/enquêteur. Des informations sur l'accès et l'utilisation des services de santé, sur la santé maternelle et infantile, sur votre couple ainsi que des questions d'opinions et d'attitudes vous seront demandées.

L'entretien prend généralement entre 30 et 90 minutes. Nous avons pris les dispositions idoines pour que toute information que vous nous fournirez reste strictement secrète. L'entretien va être confidentiel et se tiendra en privé. Si les conditions pour que l'entretien se tienne en privé ne sont pas réunies, nous allons arrêter l'interview jusqu'à ce que celles-ci le soient. L'exploitation de l'information sera et restera anonyme.

Vous ne tirerez pas d'avantages directs de cette enquête, mais les informations que vous allez nous fournir aideront le Gouvernement à planifier les actions futures dans le domaine de la santé de la reproduction.

Il n'y a pas d'inconvénients à participer à cette enquête, hormis le fait de rester chez vous et de répondre aux questions posées. Aucun prélèvement ou traitement n'est envisagé dans le cadre de cette enquête. La participation à cette enquête est volontaire; s'il y a une question à laquelle vous ne voulez pas répondre, faites-le savoir et l'enquêteur passera à la question suivante. Vous pouvez également renoncer à l'entretien à tout moment. Nous espérons cependant que vous participerez à cette enquête car votre opinion est particulièrement importante pour nous.

Si vous acceptez de participer, vous devez signer ce document pour signifier que vous avez reçu l'information et marquer votre accord. Une copie du document signé vous sera remise.
Vous pouvez contacter le Directeur du Centre de Recherche pour le Développement Humain, HLM Hann Mariste, Im. A, # 7A/7B. Téléphone : 33 832 63 79, à tout moment si vous avez des questions au sujet de l'enquête.

Vous pouvez contacter Dr. Samba Cor Sarr, Direction de la Santé, MSP, 1 Rue Aimé Césaire, 2ème étage, Dakar Fann. BP 4024, Tél : 33 869 43 13, à tout moment si vous avez des questions au sujet de vos droits en tant que participant à l'enquête.

Votre participation à cette enquête est volontaire, ni votre entourage ni vous-même, ne serez pénalisés, ni ne perdrez aucun de vos droits si vous décidez d'arrêter votre participation.

Fait à_______________, le _______________

Signature de l'enquêteur/trice

S	GR	N°MEN	N°LIGN

Fiche de consentement libre et éclairé pour l'homme dans l’enquête ménage

Je soussigné Mr ___________________________ atteste avoir reçu la lettre d’information contenant les explications détaillées sur le déroulement de l’enquête et les conditions de ma participation et avoir reçu une réponse satisfaisante à toutes les questions que j’ai posées.

Certifie avoir donné mon accord volontaire et libre de participer à l’enquête.

Je retiens garder mon droit de suspendre ma participation à cette étude à tout moment et sans aucun préjudice.

Si j’ai des questions au sujet de l’enquête, je peux contacter à tout moment le Directeur du Centre de Recherche pour le Développement Humain, Téléphone : 33 832 63 79.

Au sujet des questions sur mes droits en tant que participant à l’enquête, je peux à tout moment contacter Dr. Samba Cor Sarr, Direction de la Santé, MSP, 1 Rue Aimé Césaire, 2ème étage, Dakar Fann. BP 4024, Tél : 33 869 43 13.

En foi de quoi, j’accepte de signer cette fiche de consentement pour servir et valoir ce que de droit.

Fait à_______________, le _______________ Fait à_______________, le _______________

Signature de l’enquêteur/trice Signature Participant

SECTION 1: CARACTERISTIQUES DE BASE

	Questions et filtres	Modalités de codage	Aller à
	ENREGISTREZ L'HEURE	HEURE……….. ☐☐<br>MINUTES…..…. ☐☐	
Merci d'avoir accepté de participer à l'enquête. Comme je l'ai mentionné lorsque je vous ai demandé votre consentement, nous cherchons à évaluer vos besoins en santé et en informations. Pour commencer, je vais vous poser des questions de base sur vous-mêmes.			
Q101	En quel mois et en quelle année êtes-vous né?	MOIS…………….………………. ☐☐<br>NE CONNAIT PAS LE MOIS…………. 98<br>ANNEE……..………. ☐☐☐☐<br>NE CONNAIT PAS L'ANNEE……… 9998	
Q102	Quel âge aviez-vous à votre dernier anniversaire ?<br><br>**COMPAREZ ET CORRIGEZ 101 ET/OU 102 SI INCOHERENT**	AGE EN ANNEES REVOLUES ☐☐	
Q103	VERIFIEZ 101 & 102<br>A-T-IL ENTRE 15 ET 59 ANS ?<br><br>SI OUI, CONTINUER L'INTERIEW : ☐	SI NON : ☐	**ARRETER L'INTER-VIEW**
Q104	Depuis combien de temps vivez-vous de manière continue dans cette maison ou cet appartement?<br><br>**SI DE QUELQUES MOIS A 11 MOIS, ENREGISTRER 00-11. AUTREMENT, ENREGISTRER NOMBRE D'ANNEES.**	MOIS……………………………. 1 ☐☐<br>ANNEES…………..……………... 2 ☐☐<br>TOUJOURS………………..…………. 995<br>VISITEUR…..………………..……………. 996	 <br>**Q107**<br>**Q107**
Q105	Juste avant d'habiter dans cette maison/cet appartement, viviez-vous à Mbao, Guédiawaye, Pikine, Dakar, Mbour, Kaolack, ou ailleurs?<br><br>**SI LA REPONSE EST LE NOM D'UNE REGION, DEMANDER S'IL S'AGIT D'UNE ZONE RURALE OU D'UNE ZONE URBAINE. SI LA REPONSE EST KAOLACK OU MBOUR, ENCERCLER LES CODES '06' OU '05' SI SEULEMENT IL S'AGIT DES COMMUNES CORRESPONDANTES.**	MBAO………………………………………. 01<br>GUEDIAWAYE………………………………. 02<br>PIKINE………………………………………. 03<br>DAKAR………………………………………. 04<br>MBOUR………………………………………. 05<br>KAOLACK……………………………………. 06<br>AUTRE VILLE………………………………. 07<br>ZONE RURALE…………………………….. 08<br>ÉTRANGER……..…………………………… 09	01–06 → **Q107**<br><br>09 → **Q107**
Q106	Quel est le nom et l'emplacement de ce lieu?	NOM VILLE/VILLAGE________________________<br>________________________________<br><br>NOM DEPARTEMENT ______________ ☐☐<br>A CODIFIER AU BUREAU<br>NOM REGION __________________ ☐☐<br>A CODIFIER AU BUREAU	
Q107	Au cours des 12 derniers mois, à combien d'occasions différentes avez-vous voyagé hors de votre lieu résidence régulière et dormi ailleurs pendant au moins une nuit ?	NOMBRE DE DEPLACEMENTS... ☐☐<br>AUCUN ………………………………………. 00	 **Q109**
Q108	Au cours des 12 derniers mois, vous êtes-vous absenté de votre domicile pendant plus d'un mois d'affilé ?	OUI…………………………..…………………. 1<br>NON…………………………………………. 2	
Q109	Avez-vous déjà fréquenté l'école?	OUI…………………………..…………………. 1<br>NON…………………………………………. 2	 **Q114**
Q110	Quel est le plus haut niveau d'études que vous avez atteint: Primaire, secondaire, ou supérieur?	PRIMAIRE………………………………….. 1<br>SECONDAIRE 1……………………………. 2<br>SECONDAIRE 2……………………………. 3<br>SUPERIEUR…………………………………. 4	
Q111	Quelle est (l'année/classe) la plus élevée que vous avez achevée à ce niveau ?<br>**SI AUCUNE ANNEE/CLASSE ACHEVEE A CE NIVEAU, REPORTEZ "00"**	CLASSE / ANNEE ……………. ☐☐	

Q112	**VERIFIEZ 110:**<br>**PRIMAIRE (Q110=1) :** ☐ ↓	**SECONDAIRE OU SUPERIEUR (Q110=2, 3, 4) :** ☐ →	**Q115**
Q114	Maintenant, je voudrais vous demander de me lire cette phrase.<br><br>**MONTREZ A L'ENQUETE UNE PHRASE FIGURANT SUR LA CARTE D'ALPHABETISATION**<br><br>**SI L'ENQUETE NE PEUT PAS LIRE LA PHRASE ENTIERE, INSISTEZ** : Pouvez-vous me lire certaines parties de la phrase?	NE PEUT PAS LIRE DU TOUT ……………… 1<br>PEUT LIRE SEULEMENT CERTAINES PARTIES DE LA PHRASE …………… 2<br>PEUT LIRE TOUTE LA PHRASE …………. 3<br>PAS DE CARTE DANS LA LANGUE QUI CONVIENT ________________ 4<br>(PRECISER LA LANGUE)<br>AVEUGLE/MALVOYANT………………….…… 5	
Q115	Quelle est votre religion?	CATHOLIQUE………………………………… 1<br>AUTRE RELIGION CHRETIENNE…………. 2<br>MUSULMAN…………………………………… 3<br>TRADITIONNELLE…………………………… 4<br>AUCUNE RELIGION………………………… 5 →<br>AUTRE___________________________ 6<br>(PRECISER)	**SQ1**
Q116	Dans quelle mesure vos croyances religieuses influencent-elles vos décisions en matière de PF ? Diriez vous: Pas du tout, quelques fois, souvent ou toujours?	PAS DU TOUT………………………………… 1<br>PARFOIS… …………………………………… 2<br>SOUVENT……………………………………… 3<br>TOUJOURS…………………………………… 4<br>NE CONNAIT PAS LA PF…………………… 8	
SQ1	Êtes-vous sénégalais ?	OUI…………………………………………… 1<br>NON……………………………………………… 2 →	**Q118**
Q117	Quel est votre groupe ethnique?	WOLOF/LEBOU……………………………… 01<br>POULAR……………………………………… 02<br>SERER………………………………………… 03<br>DIOLA…………………………………………… 04<br>MANDINGUE………………………………… 05<br>SONINKE……………………………………… 06<br>AUTRE___________________________ 96<br>(PRECISER)	
Q118	Que faites-vous comme travail, c'est-à-dire, quel genre de travail faites-vous principalement ?	NOM DE L'OCCUPATION ACTUELLE<br>________________________________<br>________________________________<br>☐☐☐ *RESERVE AU CODAGE BUREAU*	
Q119	Au cours des 12 derniers mois, avez-vous eu un travail, quel qu'il soit ?	OUI…………………………………………… 1 →<br>NON……………………………………………… 2	**Q121**
Q120	Qu'avez-vous fait la plupart du temps au cours des 12 derniers mois?	RIEN…………………………………………… 01<br>A LA RECHERCHE D'UN EMPLOI………… 02<br>ALLER A L'ECOLE/ ETUDIER ……………… 03<br>INCAPABLE DE TRAVAILLER/ HANDICAPE……………………………… 04<br>RETRAITE …………………………………… 05<br>TRAVAUX DOMESTIQUES/S'OCCUPER DES ENFANTS ……………………………… 06<br>AUTRE___________________________ 96<br>(A PRECISER)	**Q201**
Q121	Etes-vous employé par un membre de votre famille, par quelqu'un d'autre, ou vous auto-employez-vous ?	POUR UN MEMBRE DE LA FAMILLE……. 1<br>POUR QUELQU'UN D'AUTRE……………… 2<br>S'AUTO-EMPLOIE…………………………… 3	
Q122	Travaillez-vous d'habitude pendant toute l'année, de manière saisonnière, ou seulement de temps en temps?	PENDANT TOUTE L'ANNEE ……………… 1<br>DE MANIERE SAISONNIERE/UNE PARTIE DE L'ANNEE ………………………… 2<br>DE TEMPS EN TEMPS ……………………… 3	
Q123	Etes-vous payé en espèces ou en nature pour ce travail, ou n'êtes-vous pas payé du tout?	SEULEMENT EN ESPECES ……………… 1<br>EN ESPECES ET EN NATURE …………… 2<br>SEULEMENT EN NATURE ………………… 3<br>PAS PAYE ……………………………………… 4	

SECTION 2: REPRODUCTION			
	**Questions et filtres**	**Modalités de codage**	**ALLER A**
Maintenant, je voudrais vous poser des questions sur vos enfants. S'il vous plaît, soyez le plus honnête possible et sachez que vos réponses ne seront communiquées à personne d'autre.			
Q201	Maintenant, je voudrais vous interroger sur les enfants que vous avez eus pendant votre vie. Je suis uniquement intéressé par les enfants qui sont biologiquement les vôtres.<br><br>Avez-vous déjà été/Êtes-vous pére d'un ou de plusieurs enfants?	OUI………………………………………… 1<br>NON………………………………………… 2<br>NE SAIT PAS……………………………… 8	 → **Q206**<br>→ **Q206**
Q202	Y a-t-il des fils ou des filles dont vous êtes le père et qui vivent actuellement avec vous ?	OUI………………………………………… 1<br>NON………………………………………… 2	 → **Q204**
Q203	Combien de fils vivent avec vous ?<br><br>Et combien de filles vivent avec vous?	FILS VIVANT A LA MAISON….. [ ][ ]<br><br>FILLES VIVANT A LA MAISON.. [ ][ ]	
Q204	Y a-t-il des fils ou des filles dont vous êtes père, qui sont toujours en vie, mais qui ne vivent pas avec vous ?	OUI………………………………………… 1<br>NON………………………………………… 2	 → **Q206**
Q205	Combien de fils sont en vie mais ne vivent pas avec vous?<br><br>Et combien de filles sont en vie mais ne vivent pas avec vous ?	FILS VIVANT AILLEURS ………. [ ][ ]<br><br>FILLES VIVANT AILLEURS …… [ ][ ]	
Q206	Avez-vous eu un fils ou une fille qui est né vivant(e) mais qui est décédé(e) par la suite ?<br><br>**SI NON, INSISTEZ: UN BEBE AYANT CRIE OU AYANT MONTRE DES SIGNES DE VIE, MAIS QUI N'A PAS SURVECU?**	OUI………………………………………… 1<br>NON………………………………………… 2<br>NE SAIT PAS……………………………… 8	 → **Q208**<br>→ **Q208**
Q207	Combien de garçons sont décédés?<br><br>Et combien de filles sont décédées?	FILS DECEDES…………………… [ ][ ]<br><br>FILLES DECEDEES …………… [ ][ ]	
Q208	FAIRE LA SOMME DES REPONSES AUX QUESTIONS Q203, Q205, ET Q207, ET ECRIVEZ LE TOTAL<br><br>**S'IL N'Y EN A AUCUN, ECRIVEZ '00'.**	TOTAL D'ENFANTS …………… [ ][ ]	
Q209	**VERIFIEZ Q208:**<br>A UN ENFANT OU DES ENFANTS : [ ] ↓	N'A PAS EU D'ENFANTS : [ ]	→ **Q214**
Q210	Quel était votre âge lorsque vous êtes devenu père <u>pour la première fois</u> ?<br>(AGE EN ANNEES REVOLUES)	AGE EN ANNEES ……………… [ ][ ]<br>NE SAIT PAS/NE SE RAPPELLE PAS…… 98	
Q211	Quel âge a votre plus jeune enfant vivant (dernier-né)?<br><br>**(AGE EN MOIS SI MOINS D'UN AN ; SI MOINS D'UN MOIS, METTEZ 00; AGE EN ANNEES REVOLUES SI PLUS D'UN AN)**	AGE EN MOIS…………………… [ ][ ]<br>AGE EN ANNEES ……………… [ ][ ]<br>PAS D'ENFANTS VIVANTS……………… 995<br>NE SAIT PAS/NE SE RAPPELLE PAS ….. 998	
Q212	Au moment où vous avez eu votre plus jeune enfant (dernier-né), vouliez-vous avoir des enfants <u>à ce moment,</u> vouliez-vous attendre <u>plus tard,</u> ou vouliez-vous <u>ne plus/ne pas avoir d'enfants</u> ?	A CE MOMENT ……………………………… 1<br>PLUS TARD…………………………………… 2<br>NE PAS/NE PLUS AVOIR D'ENFANTS … 3	→ **Q214**<br><br>→ **Q214**

Q213	Combien de temps de plus auriez-vous souhaité attendre pour avoir votre (plus jeune) enfant ?	MOIS……………………………… 1 [ ][ ]<br>ANNEES……………………….. 2 [ ][ ]<br>AUTRE ____________________ 996<br>NE SAIT PAS……………………… 998	
Q214	*A présent, je voudrais vous poser des questions sur les risques de survenue d'une grossesse chez la femme.*<br>Entre la période des règles et les règles suivantes, y-a-t-il un moment où une femme a plus de chances de tomber enceinte que d'autres, si elle a des rapports sexuels ?	OUI ……………………………………………… 1<br>NON……………………………………………… 2<br>NSP……………………………………………… 8	 <br>→ **Q216**<br>→ **Q216**
Q215	A quelle période du cycle menstruel ces jours correspondent t-ils ? Juste avant le début des règles, Pendant les règles, Juste après la fin des règles, Au milieu entre 2 périodes ?<br><br>**UNE SEULE REPONSE EST ENREGISTREE.**	JUSTE AVANT LES REGLES…………………… 1<br>PENDANT LES REGLES………………………… 2<br>TOUT JUSTE APRES LA FIN DES REGLES… 3<br>AU MILIEU ENTRE 2 PERIODES……………… 4<br>AUTRE______________________________ 6<br>(PRECISER)<br>NE SAIT PAS……………………………………… 8	
Q216	Pensez-vous qu'une femme qui allaite son bébé au sein peut tomber enceinte ?	OUI ……………………………………………… 1<br>NON……………………………………………… 2<br>CELA DEPEND………………………………… 3<br>NSP……………………………………………… 8	 <br><br>→ **Q301**
Q218	Dans quelles conditions l'allaitement maternel peut-être considéré comme une méthode contraceptive efficace ?<br><br>**INSISTER: Quelque chose d'autre?**<br><br>**ENREGISTRER TOUT CE QUI EST MENTIONNE.**	ALLAITEMENT MATERNEL EXCLUSIF (PAS DE SUPPLEMENTS)…………… A<br>ALLAITEMENT MATERNEL EXCLUSIF JUSQU' A 6 MOIS……………………… B<br>AVANT LE RETOUR DE COUCHES……… C<br>AUTRES____________________________ X<br>(PRECISER)	

SECTION 3: CONTRACEPTION ET IMPLICATION DE L'HOMME			
	VERIFIEZ LA PRESENCE D'AUTRES PERSONNES. AVANT DE CONTINUER, FAITES TOUT POUR GARANTIR UN CADRE INTIME.		
	Maintenant, je voudrais parler de planification familiale, des divers moyens ou des diverses méthodes qu'un couple peut utiliser pour retarder ou éviter une grossesse.		
	Quels sont les moyens ou les méthodes dont vous avez entendu parler?<br>**POUR LES METHODES QUI N'ONT PAS ETE MENTIONNEES SPONTANEMENT, DEMANDEZ:**<br><br>ENCERCLEZ '1' POUR CHAQUE METHODE MENTIONNEE SPONTANEMENT. PUIS PROCEDEZ EN LISANT DANS LA COLONNE EN BAS LE NOM ET LA DESCRIPTION DE CHAQUE METHODE QUI N'A PAS ETE MENTIONNEE SPONTANEMENT. ENCERCLEZ '2' POUR CHAQUE METHODE RECONNUE APRES ECOUTE DE LA DESCRIPTION. ENCERCLEZ '3' POUR CHAQUE METHODE QUI N'A PAS ÉTÉ MENTIONNEE NI RECONNUE.		
		301. Avez-vous déjà entendu parler de (METHODE)?<br><br>OUI(Mentionnée spontanément)…1<br>OUI (Après description).?…..……..2<br>NON……….………………………...…3	302. Avez-vous ou votre (vos) partenaire(s) déjà utilisé (METHODE)?
1	LA STERILISATION FEMININE les femmes peuvent subir une opération pour éviter d'avoir d'autres d'enfants.	1 2 3<br>↓<br>METHODE SUIVANTE	Votre partenaire a-t-elle jamais effectué une opération de stérilisation pour éviter d'avoir des enfants ?<br>OUI………. 1<br>NON……… 2
2	LA STERILISATION MASCULINE/VASECTOMIE<br>Les hommes peuvent subir une opération pour éviter d'avoir d'autres d'enfants	1 2 3<br>↓<br>METHODE SUIVANTE	Avez-vous jamais effectué une opération (vasectomie) pour éviter d'avoir davantage d'enfants ?<br>OUI…..…… 1<br>NON……… 2
3	LES PILULES JOURNALIERES Les femmes peuvent avaler une pilule chaque jour pour éviter de tomber enceinte.	1 2 3<br>↓<br>METHODE SUIVANTE	OUI………. 1<br>NON……… 2<br>NSP……… 8
4	DIU Les femmes peuvent avoir un stérilet que le médecin, la sage-femme ou l'infirmier/ière leur place à l'intérieur de l'utérus.	1 2 3<br>↓<br>METHODE SUIVANTE	OUI………. 1<br>NON……… 2<br>NSP……… 8
5	INJECTABLES Les femmes peuvent se faire faire une injection par un prestataire de santé qui les empêche de tomber enceinte pendant un ou plusieurs mois.	1 2 3<br>↓<br>METHODE SUIVANTE	OUI………. 1<br>NON……… 2<br>NSP……… 8
6	IMPLANTS Les femmes peuvent se faire placer sous la peau de la partie supérieure du bras un ou plusieurs petits bâtonnets qui vont les empêcher de tomber enceinte pendant un ou plusieurs années.	1 2 3<br>↓<br>METHODE SUIVANTE	OUI………. 1<br>NON……… 2<br>NSP……… 8
7	PRESERVATIF MASCULIN Les hommes peuvent porter un préservatif en caoutchouc sur leur pénis avant l'acte sexuel.	1 2 3<br>↓<br>METHODE SUIVANTE	OUI………. 1<br>NON……… 2
8	PRESERVATIF FEMININ Les femmes peuvent placer un préservatif dans leur vagin avant l'acte sexuel.	1 2 3<br>↓<br>METHODE SUIVANTE	OUI………. 1<br>NON……… 2<br>NSP……… 8

9	LA METHODE DU RYTHME chaque mois, pendant qu'elle est sexuellement active, une femme peut éviter de tomber enceinte en n'ayant pas de rapports sexuels les jours du mois pendant lesquels elle court beaucoup de risques de tomber enceinte.	1 2 3 → METHODE SUIVANTE	OUI………. 1<br>NON……… 2	
10	LA METHODE DU RETRAIT Les hommes peuvent être prudents et se retirer avant d'éjaculer.	1 2 3 → METHODE SUIVANTE	OUI………. 1<br>NON……… 2	
11	CONTRACEPTION D'URGENCE Les femmes peuvent prendre des pilules jusqu'à cinq jours après un rapport sexuel pour éviter de tomber enceinte.	1 2 3 → METHODE SUIVANTE	OUI………. 1<br>NON……… 2<br>NSP………. 8	
12	METHODE DE L'ALLAITEMENT MATERNEL ET DE L'AMENORRHEE (MAMA) Jusqu'à 6 mois après l'accouchement, une femme peut utiliser une méthode qui exige qu'elle allaite fréquemment son bébé, le jour comme la nuit, et à condition qu'elle n'ait pas encore eu le retour de couche.	1 2 3 → METHODE SUIVANTE	OUI………. 1<br>NON……… 2<br>NSP………. 8	
13	SPERMICIDE/MOUSSE/GEL Les femmes peuvent introduire un suppositoire, un gel ou de la crème dans leur vagin avant l'acte sexuel.	1 2 3 → METHODE SUIVANTE	OUI………. 1<br>NON……… 2<br>NSP………. 8	
14	Avez-vous entendu parler d'autres moyens ou d'autres méthodes que les femmes ou les hommes peuvent utiliser pour éviter la grossesse ?<br>**NOTE: SI L'ENQUETE MENTIONNE L'ABSTINENCE COMME UNE METHODE DE PF, NE PAS LE REPORTER. INSISTER POUR TOUTE AUTRE METHODE CONNUE.**	OUI…………………………… 1<br>____________________<br>(A PRECISER)<br>NON………………………… 3	OUI………. 1<br>NON……… 2<br>NSP………. 8	
15		OUI…………………………… 1<br>____________________<br>(A PRECISER)<br>NON………………………… 3	OUI………. 1<br>NON……… 2<br>NSP………. 8	
Q303	**VERIFIEZ Q301**<br>**SI L'ENQUETE A ENTENDU PARLER D'AU MOINS UNE METHODE (Q301=1 OU 2)** ☐ ↓	**SI LA REPONSE EST 'NON' (3) A TOUTES** ☐ →		**Q401**

Q304	Au cours de 12 derniers mois, où avez-vous vu ou entendu parler de contraception ?<br><br>INSISTER: D'AUTRES SOURCES?<br><br>ENREGISTRER TOUT CE QUI EST MENTIONNE	**MEDIA**<br>RADIO……… AA<br>TELEVISION……… AB<br>JOURNAL……… AC<br>MAGAZINE……… AD<br>PANNEAUX D'AFFICHAGE……… AE<br>ÉVENEMENTS COMMUNAUTAIRES……… AF<br>THEATRE SUR SCENE……… AG<br>**SECTEUR PUBLIC**<br>HOPITAL GOUVERNEMENTAL……… BA<br>CENTRE SANTE GOUVERNEMENTAL……… BB<br>POSTE DE SANTE……… BC<br>STRATEGIE AVANCEE/EQUIPE MOBILE……… BD<br>CENTRE CONSEILS ADOS……… BE<br>CASE DE SANTE……… BF<br>AUTRE PUBLIC……… BG<br>**SECTEUR PRIVE FORMEL**<br>HOPITAL/CLINIQUE/CABINET PRIVE……… CA<br>PHARMACIE……… CB<br>DISPENSAIRE RELIGIEUX……… CC<br>AUTRE MEDICAL PRIVE……… CD<br>**SECTEUR PRIVE INFORMEL**<br>MEDECIN……… DA<br>SAGE-FEMME……… DB<br>INFIRMIER/AI……… DC<br>MATRONE / ASC ……… DD<br>GUERISSEUR /ACCOUCHEUSE DE<br>**AUTRES SOURCES**<br>ÉCOLE……… EA<br>ÉGLISE/MOSQUEE……… EB<br>BAR……… EC<br>PARENTS/AMIS ……… ED<br>ONG/OCB……… EE<br>**AUTRE**____________ XX<br>(PRECISER)<br>N'A NI VU, NI ENTENDU……… YY<br>NSP……… ZZ	
Q305	**VERIFIEZ Q302.**<br>A DEJA UTILISE AU MOINS UNE METHODE (AU MOINS UN "1" DANS Q302) : ☐ ↓	N'A JAMAIS UTILISE UNE METHODE (PARTOUT "2" A Q302) : ☐ →	**Q322**
Q306	Maintenant, je voudrais vous poser des questions sur la <u>première fois</u> que vous avez fait quelque chose ou utilisé une méthode pour éviter d'avoir un enfant.<br>Combien d'enfants vivants aviez-vous en ce moment?<br>Combien de fils vivants aviez-vous en ce moment?<br>Combien de filles vivants aviez-vous en ce moment?<br><br>**S'IL N'Y EN A AUCUN, ENREGISTREZ '00'.**	NOMBRE D'ENFANTS……… ☐☐<br>NOMBRE DE FILS……… ☐☐<br>NOMBRE DE FILLES……… ☐☐	
Q307	Vous (ou votre femme/partenaire) faites-vous quelque chose ou utilisez-vous une méthode actuellement pour retarder ou éviter d'avoir une grossesse ?	OUI……… 1<br>NON……… 2<br>NSP……… 8	 → **Q322**<br>→ **Q322**
Q308	Quelles méthodes utilisez-vous actuellement vous et votre femme/partenaire dans votre couple ?<br><br>SI PILULE, INSISTER POUR SAVOIR SI PILULE JOURNALIERE OU CONTRACEPTION D'URGENCE.<br><br>INSISTER: D'autres méthodes?<br><br>ENCERCLER TOUT CE QUI EST MENTIONNE.	STERILISATION FEMININE ……… A<br>STERILISATION MASCULINE……… B<br>IMPLANT……… C<br>DIU ……… D<br>INJECTABLES……… E<br>PILULE……… F<br>CONTRACEPTION D'URGENCE……… G<br>PRESERVATIF MASCULIN……… H<br>PRESERVATIF FEMININ……… I<br>SPERMICIDE/MOUSSE/GEL……… J<br>METHODES NATURELLES (METHODE DU RYTHME /ABSTINENCE PERIODIQUE / RETRAIT)……… K<br>ALLAITEMENT AU SEIN /MAMA……… L<br>AUTRE____________ X<br>(PRECISEZ)	

Q309	**VERIFIEZ Q308 :**<br>**SI PLUSIEURS METHODES MENTIONNEES, ENCERCLEZ DANS CETTE LISTE CELLE DONT LE CODE EST ARRIVE LE PREMIER PAR ORDRE ALPHABETIQUE ET CONTINUEZ AVEC CELLE-CI POUR LES QUESTIONS SUIVANTES.**<br><br>**SI UNE SEULE METHODE EST MENTIONNEE, ENCERCLEZ CETTE MÊME METHODE DANS CETTE LISTE.**	STERILISATION FEMININE .... 01<br>STERILISATION MASCULINE....................................... 02<br>IMPLANT.. 03<br>DIU .... 04<br>INJECTABLES... 05<br>PILULE.. 06<br>CONTRACEPTION D'URGENCE........................ 07<br>PRESERVATIF MASCULIN.. 08<br>PRESERVATIF FEMININ.. 09<br>SPERMICIDE/MOUSSE/GEL...................................... 10<br>METHODES NATURELLES<br>(METHODE DU RYTHME /ABSTINENCE<br>PERIODIQUE / RETRAIT)........................ 11<br>ALLAITEMENT AU SEIN /MAMA.............................. 12<br>AUTRE_______________________________ 96<br>(PRECISEZ)	
Q310	Pourquoi avez-vous choisie d'utiliser cette méthode? (METHODE ENCERCLEE EN Q309)<br><br>INSISTEZ : Existe-t-il une autre raison ?<br><br>ENREGISTRER TOUT CE QUI EST MENTIONNE.	NE VEUT PAS RENDRE ENCEINTE................................. A<br>PAS D'EFFETS SECONDAIRES.. B<br>NE VEUT PAS ÊTRE INFECTÉ PAR LE<br>VIH OU LES AUTRES ISTs... C<br>FACILE / PRATIQUE À UTILISER...................................... D<br>METHODE DISCRETE.. E<br>ABORDABLE... F<br>FACILE A OBTENIR... G<br>BEAUCOUP DE PERSONNES L'UTILISENT...................... H<br>J'AIME COMME ON LE PREND AU QUOTIDIEN............... I<br>J'AIME COMME ON NE LE PREND PAS<br>AU QUOTIDIEN.. J<br>FAIT PERDRE DU POIDS A MA PARTENAIRE.................. K<br>FAIT GAGNER DU POIDS A MA PARTENAIRE................. L<br>DONNE/MAINTIENT BON TEINT A PARTENAIRE............. M<br>RECOMMANDÉ PAR UN PRESTATAIRE.......................... N<br>MA PARTENAIRE LA PRÉFÈRE...................................... O<br>JE N'AI PAS À M'OCCUPER DE ÇA; MA<br>PARTENAIRE EST RESPONSABLE DU CHOIX............ P<br>AUTRE ___________________________________ X<br>(PRÉCISER)	
Q311	Qui décide de quelle méthode contraceptive utiliser ou ne pas utiliser ? Est-ce vous principalement, votre partenaire principalement, ou décidez-vous ensemble?	VOUS PRINCIPALEMENT.............................. 1<br>PARTENAIRE PRINCIPALEMENT................. 2<br>CONJOINTEMENT.. 3<br>AUTRE______________________________ 6<br>(PRECISER)	
Q312	**VERIFIEZ Q309:**<br>**SI PRESERVATIF MASCULIN "8"** ☐ ↓	**SI STERILISATION MASCULINE "2"** ☐<br><br>**SI STERILISATION FEMININE "1", IMPLANTS "3", DIU "4", INJECTABLES "5", PILULES "6", CONTRACEPTION D'URGENCE "7", PRESERVATIF FEMININ "9", SPERMICIDE "10", OU AUTRE PRODUIT "96"** ☐<br><br>**SI METHODES NATURELLES "11", MAMA "12", OU AUTRE METHODE NATURELLE EN "96" (POSTPARTUM, ABSTINENCE, ETC)** ☐	**Q318**<br><br>**Q316**<br><br>**Q329**
Q313	Où (de qui) aviez-vous obtenu <u>la dernière fois</u> les condoms ?<br><br>**DEMANDEZ A CONNAITRE LE NOM ET L'EMPLACEMENT DE LA STRUCTURE ET ENREGISTREZ.**	NOM ________________________________<br><br>LIEU ________________________________<br>ADRESSE PHYSIQUE ET DESCRIPTION<br>______________________________________	

Q314	Quel est le type de place/personne?<br><br>**ENCERCLEZ LE TYPE DE STRUCTURE APPROPRIE.**	**SECTEUR PUBLIC**<br>HOPITAL GOUVERNEMENTAL........ 11<br>CENTRE SANTE GOUVERNEMENTAL........ 12<br>POSTE DE SANTE........ 13<br>STRATEGIE AVANCEE/EQUIPE MOBILE........ 14<br>CENTRE CONSEILS ADOS........ 15<br>CASE DE SANTE........ 16<br>AUTRE PUBLIC........ 17<br><br>**SECTEUR PRIVE FORMEL**<br>HOPITAL/CLINIQUE/CABINET PRIVE........ 21<br>PHARMACIE........ 22<br>DISPENSAIRE RELIGIEUX........ 23<br>AUTRE MEDICAL PRIVE........ 24<br><br>**SECTEUR PRIVE INFORMEL**<br>MEDECIN........ 31<br>SAGE-FEMME........ 32<br>INFIRMIER/AI........ 33<br>MATRONE / ASC .... 34<br>GUERISSEUR /ACCOUCHEUSE TRADITIONNELLE........ 35<br><br>**AUTRES SOURCES**<br>CLINIQUE AU LIEU DE TRAVAIL........ 41<br>CENTRE POUR JEUNE........ 42<br>MACHINE A VENDRE........ 43<br>CENTRE DE CONSEIL/TEST DE VIH........ 44<br>BAR........ 45<br>KIOSQUE/BOUTIQUE/MARCHE........ 46<br>VOLONTAIRES/PAIRS ÉDUCATEURS........ 47<br>ONG........ 48<br>**AUTRE**________ 96<br>(PRECISER)<br>NSP........ 98	
Q315	Combien de préservatifs (**unités**) avez-vous payés / obtenues la dernière fois ?	NOMBRE DE PRESERVATFS........ [ ][ ]<br><br>NE SAIT PAS........98	
Q316	Quel est le prix que vous avez payé pour cette méthode **(METHODE ENREGISTREE A Q309)** la dernière fois?	MONTANT (FCFA).. [ ][ ][ ][ ][ ][ ]<br>GRATUIT........0000000<br>NE SAIT PAS........9999998	 → **Q329**<br>→ **Q329**
Q317	Diriez-vous que ce prix est bas, normal ou cher?	PRIX BAS........ 1<br>PRIX NORMAL........ 2<br>PRIX CHER........ 3	1, 2, 3 → **Q329**
Q318	POUR LES HOMMES AYANT ETE STERILISES,<br>En quel mois et en quelle année la stérilisation avait t-elle été réalisée?<br><br>Combien d'enfants aviez-vous à ce moment-là?<br>Nombre de garçons?<br>Nombre de filles ?	MOIS........ [ ][ ]<br>NE CONNAIT PAS LE MOIS........ 98<br>ANNEE........ [ ][ ][ ][ ]<br>NE CONNAIT PAS L'ANNEE........9998<br><br>NOMBRE D'ENFANTS........ [ ][ ]<br>FILS........ [ ][ ]<br>FILLES .... [ ][ ]	

Q319	Où avez-vous effectué l'opération de stérilisation?	**SECTEUR PUBLIC**<br>HÔPITAL GOUVERNEMENTAL.......... 11<br>CENTRE SANTÉ GOUVERNEMENTAL.......... 12<br>POSTE DE SANTÉ.......... 13<br>STRATÉGIE AVANCÉE/ÉQUIPE MOBILE.......... 14<br>CENTRE CONSEILS ADOS.......... 15<br>AUTRE MEDICAL PUBLIQUE.......... 16<br>**SECTEUR PRIVÉ FORMEL**<br>HÔPITAL/CLINIQUE/CABINET PRIVÉ.......... 21<br>PHARMACIE.......... 22<br>DISPENSAIRE RELIGIEUX.......... 23<br>AUTRE MÉDICAL PRIVÉ.......... 24<br>**SECTEUR PRIVÉ INFORMEL**<br>MÉDECIN.......... 31<br>SAGE-FEMME.......... 32<br>INFIRMIER/AI.......... 33<br>MATRONE / ASC .... 34<br>GUÉRISSEUR/ACCOUCHEUSE TRADITIONNELLE... 35<br>**AUTRE**<br>ONG/OCB .... 41<br>AUTRE STRUCTURE________________ 96<br>(PRECISEZ)<br>NSP.......... 98	
Q320	Quel est le prix que vous avez payé pour l'opération de stérilisation ?	MONTANT (FCFA).. [ ][ ][ ][ ][ ][ ]<br>GRATUIT..........0000000<br>NE SAIT PAS..........9999998	 → **Q329**<br>→ **Q329**
Q321	Diriez-vous que ce prix est bas, normal ou cher?	PRIX BAS.......... 1<br>PRIX NORMAL.......... 2<br>PRIX CHER.......... 3	1–3 → **Q329**
Q322	Quelles sont les principales raisons pour lesquelles vous n'utilisez pas/plus une méthode de planification familiale pour retarder ou éviter une grossesse?<br><br>INSISTER: D'autres raisons?<br><br>**ENREGISTRERTOUT CE QUI EST MENTIONNE**.	**RAISONS DE FECONDITE**<br>RAPPORTS SEXUELS NON FREQUENTS / PAS DE RAPPORTS SEXUELS.......... A<br>PARTENAIRE/FEMME ABSENT.......... B<br>PARTENAIRE EN MENOPAUSEE/HYSTERECTOMIE.. C<br>PARTENAIRE ENCEINTE.......... D<br>PARTENAIRE ALLAITE.......... E<br>LUI OU PARTNENAIRE NE PEUT PAS EN AVOIR ...... F<br>SOUHAITE AVOIR AUTANT D'ENFANTS AUSSITOT QUE POSSIBLE.......... G<br>SOUHAITE TOMBER/ESSAIE DE TOMBER ENCEINTE.......... H<br>FEMME EN AMENORRHEE POSTPARTUM.......... I<br>**OPPOSITION A L'UTILISATION**<br>L'ENQUETE EST OPPOSE.......... J<br>LA PARTENAIRE EST OPPOSEE.......... K<br>D'AUTRES PERSONNES SONT OPPOSEES.......... L<br>INTERDICTION RELIGIEUSE.......... M<br>**MANQUE DE CONNAISSANCE** :<br>NE SAIT PAS COMMENT UTILISER UNE METHODE.......... N<br>NE CONNAIT AUCUNE SOURCE.......... O<br>**RAISONS LIEES A LA METHODE**<br>PROBLEMES DE SANTE POUR LUI OU FEMME.......... P<br>PEUR DES EFFETS SECONDAIRES.......... Q<br>MANQUE D'ACCES / TROP ELOIGNE.......... R<br>COUTE TROP CHERE.......... S<br>PAS PRATIQUE A UTILISER.......... T<br>N'AIME PAS LES METHODES EXISTANT.......... U<br>EXPERIENCES MALHEUREUSES AVEC LES METHODES EXISTANT.......... V<br>FATALISTE: DEPEND DE DIEU .... W<br>AUTRE________________ X<br>(PRECISER)<br>NE SAIT PAS .... Z	C → **Q329**<br>F → **Q329**

Q323	Pensez-vous que vous (ou votre femme/partenaire) utiliserez une méthode pour retarder ou éviter une grossesse au cours des 12 prochains mois ?	OUI…… 1<br>NON…… 2<br>NE SAIT PAS…… 8	 →Q329<br>→Q329
Q324	Quelle méthode préfèreriez-vous utiliser **LE PLUS**, si vous utilisez effectivement une méthode à l'avenir?<br><br>SI PILULE, INSISTER POUR SAVOIR SI PILULE JOURNALIERE OU CONTRACEPTION D'URGENCE.	STERILISATION FEMININE …… 01<br>STERILISATION MASCULINE…… 02<br>IMPLANT…… 03<br>DIU …… 04<br>INJECTABLES…… 05<br>PILULE…… 06<br>CONTRACEPTION D'URGENCE…… 07<br>PRESERVATIF MASCULIN…… 08<br>PRESERVATIF FEMININ…… 09<br>SPERMICIDE/MOUSSE/GEL…… 10<br>METHODES NATURELLES (METHODE DU RYTHME /ABSTINENCE PERIODIQUE / RETRAIT)…… 11<br>ALLAITEMENT AU SEIN /MAMA…… 12<br>AUTRE______ 96<br>(PRECISEZ)<br>NE SAIT PAS…… 98	 <br><br><br><br><br><br><br><br><br>→Q329<br>→Q329<br><br><br>→Q329
Q325	Connaissez-vous un endroit ou une personne où vous pouvez obtenir cette méthode (METHODE ENCERCLER A Q324)?	OUI…… 1<br>NON…… 2	 → Q327
Q326	Quel est le type de place/personne où vous pouvez obtenir cette méthode (METHODE ENCERCLER A Q324)?<br><br>**ENCERCLEZ LE TYPE DE STRUCTURE APPROPRIE.**	**SECTEUR PUBLIC**<br>HOPITAL GOUVERNEMENTAL…… 11<br>CENTRE SANTE GOUVERNEMENTAL…… 12<br>POSTE DE SANTE…… 13<br>STRATEGIE AVANCEE/EQUIPE MOBILE…… 14<br>CENTRE CONSEILS ADOS…… 15<br>CASE DE SANTE…… 16<br>AUTRE PUBLIC…… 17<br><br>**SECTEUR PRIVE FORMEL**<br>HOPITAL/CLINIQUE/CABINET PRIVE…… 21<br>PHARMACIE…… 22<br>DISPENSAIRE RELIGIEUX…… 23<br>AUTRE MEDICAL PRIVE…… 24<br><br>**SECTEUR PRIVE INFORMEL**<br>MEDECIN…… 31<br>SAGE-FEMME…… 32<br>INFIRMIER/AI…… 33<br>MATRONE / ASC …… 34<br>GUERISSEUR /ACCOUCHEUSE TRADITIONNELLE…… 35<br><br>**AUTRE**<br>CLINIQUE AU LIEU DE TRAVAIL…… 41<br>CENTRE DE JEUNES…… 42<br>CENRE DE VIH…… 43<br>AU BAR…… 44<br>AU MARCHE…… 45<br>ONG/OCB…… 46<br>VOLONTAIRES/PAIRS ÉDUCATEURS…… 47<br><br>**AUTRE**______ 96<br>(PRECISER)	
Q327	Seriez-vous prêt à payer pour obtenir cette méthode (METHODE ENCERCLER A Q324)?	OUI…… 1<br>NON…… 2<br>NE SAIT PAS…… 8	 →Q329<br>→Q329
Q328	Si OUI, combien seriez-vous prêt à payer ?	MONTANT…[ ][ ][ ][ ][ ][ ][ ]<br>(en francs CFA)	

Q329	**VERIFIEZ Q301 :**<br>**A ENTENDU PARLER DE PRESERVATIFS MASCULINS "7" CODES = 1 OU 2** ☐ ↓	**N'A JAMAIS ENTENDU PARLER DE PRESERVATIFS MASCULINS "7" CODE = 3** ☐	**Q336**
Q330	Est-il facile de vous procurer des préservatifs dans votre zone lorsque vous en avez besoin ?	OUI…… 1<br>NON…… 2<br>NE SAIT PAS…… 8	
Q331	Avez-vous jamais recommandé l'utilisation de préservatifs masculins comme une méthode de PF/Contraception à vos amis ou parents ?	OUI…… 1<br>NON…… 2	
Q332	Si un préservatif masculin est utilisé correctement, pensez-vous qu'il protège contre une grossesse la plupart du temps, seulement quelques fois, ou pas du tout ?	LA PLUPART DU TEMPS…… 1<br>SOUVENT…… 2<br>PAS DU TOUT…… 3<br>NE SAIT PAS/ PAS SUR…… 8	
Q333	Pensez-vous que l'utilisation d'un préservatif réduit le plaisir sexuel de la femme ?	OUI…… 1<br>NON…… 2<br>NE SAIT PAS…… 8	
Q334	Pensez-vous que l'utilisation d'un préservatif réduit le plaisir sexuel de l'homme ?	OUI…… 1<br>NON…… 2<br>NE SAIT PAS…… 8	
Q335	Pensez-vous que l'utilisation d'un préservatif est un signe d'infidélité?	OUI…… 1<br>NON…… 2<br>NE SAIT PAS…… 8	

Croyances - Représentations

	S'il vous plait, pouvez vous me dire si vous êtes totalement d'accord, d'accord, pas d'accord ou totalement contre les arguments suivants:	**ENTIER-MENT D'ACCORD**	**D'ACCORD**	**PAS D'ACCORD**	**TOTALEMENT CONTRE**
Q336	Se faire injecter un produit contraceptif rend la femme stérile pour toujours	4	3	2	1
Q337	Les personnes qui utilisent des contraceptifs finissent par avoir des problèmes de santé	4	3	2	1
Q338	Les contraceptifs peuvent faire du mal à l'utérus	4	3	2	1
Q339	Les contraceptifs réduisent le désir sexuel	4	3	2	1
Q340	Les contraceptifs peuvent causer le cancer	4	3	2	1
Q341	Les contraceptifs peuvent vous donner des bébés malformés	4	3	2	1
Q342	Les contraceptifs sont dangereux pour la santé	4	3	2	1

MARIAGE ET ACTIVITES SEXUELLES

	Questions et filtres	Modalités de codage	Allez à
	Maintenant, je voudrais vous poser des questions sur votre activité sexuelle et vos relations maritales. Rappelez-vous que vos réponses sont confidentielles		
Q401	Quel âge aviez-vous lorsque vous avez eu un rapport sexuel pour la toute première fois?	AGE.................. [ ][ ]<br>N'A JAMAIS EU DE RAPPORTS SEXUELS.................. 00	 → Q415
Q402	La première fois que vous avez eu un rapport sexuel, aviez-vous (ou votre partenaire) utilisé une méthode de PF?	OUI.................. 1<br>NON.................. 2<br>NE SE SOUVIENT PAS.................. 8	 → Q404<br>→ Q404
Q403	Quelle(s) méthode(s) aviez vous utilisé ?<br>INSISTER: D'autres méthodes?<br>**ENCERCLER TOUT CE QUI EST MENTIONNE**	STERILISATION FEMININE .... A<br>STERILISATION MASCULINE.................. B<br>IMPLANT.................. C<br>DIU .... D<br>INJECTABLES.................. E<br>PILULE.................. F<br>CONTRACEPTION D'URGENCE.................. G<br>PRESERVATIF MASCULIN.................. H<br>PRESERVATIF FEMININ.................. I<br>SPERMICIDE/MOUSSE/GEL.................. J<br>METHODES NATURELLES<br>(METHODE DU RYTHME /ABSTINENCE<br>PERIODIQUE / RETRAIT).................. K<br>ALLAITEMENT AU SEIN /MAMA.................. L<br>AUTRE__________ X<br>(PRECISEZ)	
Q404	Maintenant, je voudrais vous posez des questions sur votre activité sexuelle récente. Laissez-moi vous assurer encore que vos réponses resteront strictement confidentielles et ne seront communiquées à personne. Si nous arrivons à une question à laquelle vous ne souhaitez pas répondre, dites le-moi juste et nous irons à la question suivante.		
Q405	Quand avez-vous eu vos derniers rapports sexuels?<br>***SI MOINS DE 12 MOIS, LA REPONSE DOIT ETRE ENREGISTREE EN JOURS, SEMAINES OU MOIS.***<br>***SI 12 MOIS (UNE ANNEE) OU PLUS, LA REPONSE DOIT ETRE ENREGISTREE EN ANNEES.***<br>***SI MOINS D'UNE JOURNEE, ENREGISTREZ « 00 »***	IL Y A DES JOURS.................. 1 [ ][ ]<br>IL Y A DES.SEMAINES.................. 2 [ ][ ]<br>IL Y A DES MOIS.................. 3 [ ][ ]<br>IL Y A DES ANNEES.................. 4 [ ][ ]	 <br><br>→ Q415

		DERNIERE PARTENAIRE	SECONDE PARTENAIRE LA PLUS RECENTE	TROISIEME PARTENAIRE LA PUS RECENTE
Q406	Quand a lieu vos derniers rapports sexuels avec cette personne ?		JOURS........ 1 [ ][ ]<br>SEMAINES... 2 [ ][ ]<br>MOIS.......... 3 [ ][ ]<br>ANNEES...... 4 [ ][ ]	JOURS........ 1 [ ][ ]<br>SEMAINES.. 2 [ ][ ]<br>MOIS.......... 3 [ ][ ]<br>ANNEES...... 4 [ ][ ]
Q407	La dernière fois que vous avez eu un rapport sexuel avec (cette dernière/ seconde / troisième) personne, aviez-vous (ou elle) fait quelque chose ou utiliser une méthode pour éviter une grossesse?	OUI.................. 1<br>NON.................. 2<br>**Q411** ←<br>NSP/PAS SÛR........ 8	OUI.................. 1<br>NON.................. 2<br>**Q411** ←<br>NSP/PAS SÛR... 8	OUI.................. 1<br>NON.................. 2<br>**Q411** ←<br>NSP/PAS SÛR......... 8
Q408	Quelle(s) méthode(s) aviez vous utilisé?<br>**ENCERCLER TOUT CE QUI EST MENTIONNE**<br>**SI PILULE, INSISTER POUR SAVOIR SI PILULE JOURNALIERE OU CONTRACEPTION D'URGENCE**	STERILISATION FEMININE .... A<br>STERILISATION MASCULINE........ B<br>IMPLANT........ C<br>DIU .... D<br>INJECTABLES........ E<br>PILULE........ F<br>CONTRACEPTION D'URGENCE........ G<br>PRESERVATIF MASCULIN........ H<br>PRESERVATIF FEMININ........ I<br>SPERMICIDE/MOUSSE/GEL........ J<br>METHODES NATURELLES<br>(METHODE DU RYTHME /<br>ABSTINENCE / RETRAIT)........ K<br>ALLAITEMENT AU SEIN /MAMA........ L<br>AUTRE__________ X<br>(A PRECISER)	STERILISATION FEMININE .... A<br>STERILISATION MASCULINE...... B<br>IMPLANT........ C<br>DIU .... D<br>INJECTABLES........ E<br>PILULE........ F<br>CONTRACEPTION D'URGENCE. G<br>PRESERVATIF MASCULIN........ H<br>PRESERVATIF FEMININ........ I<br>SPERMICIDE/MOUSSE/GEL........ J<br>METHODES NATURELLES<br>(METHODE DU RYTHME /<br>ABSTINENCE / RETRAIT)...... K<br>ALLAITEMENT AU SEIN /MAMA.. L<br>AUTRE__________ X<br>(A PRECISER)	STERILISATION FEMININE .... A<br>STERILISATION MASCULINE........ B<br>IMPLANT........ C<br>DIU .... D<br>INJECTABLES........ E<br>PILULE........ F<br>CONTRACEPTION D'URGENCE........ G<br>PRESERVATIF MASCULIN........ H<br>PRESERVATIF FEMININ........ I<br>SPERMICIDE/MOUSSE/GEL........ J<br>METHODES NATURELLES<br>(METHODE DU RYTHME /<br>ABSTINENCE / RETRAIT)........ K<br>ALLAITEMENT AU SEIN /MAMA........ L<br>AUTRE__________ X<br>(A PRECISER)

		DERNIERE PARTENAIRE	SECONDE PARTENAIRE LA PLUS RECENTE	TROISIEME PARTENAIRE LA PUS RECENTE	
Q409	***VERIFIEZ Q408:***	***A UTILISE UNE METHODE AUTRE QUE LE PRESERVATIF*** ☐ → Q412<br><br>***PRESERVATIF MASCULIN OU FEMININ (Q408 = "H" OU "I")*** ☐	***A UTILISE UNE METHODE AUTRE QUE LE PRESERVATIF*** ☐ → Q412<br><br>***PRESERVATIF MASCULIN OU FEMININ (Q408 = "H" OU "I")*** ☐	***A UTILISE UNE METHODE AUTRE QUE LE PRESERVATIF*** ☐ →<br><br>***PRESERVATIF MASCULIN OU FEMININ (Q408 = "H" OU "I")*** ☐	**Q412**
Q410	**A UTILISE UN PRESERVATIF AU DERNIER RAPPORT**<br>Quelle était la principale raison pour laquelle vous avez utilisé un préservatif à cette occasion-là?	EVITER MALADIES.......... 1<br>EVITER GROSSESSE.......... 2<br>EVITER A LA FOIS VIH/IST.......... 3<br>N'A PAS CONFIANCE A LA PARTENAIRE.......... 4<br>LA PARTENAIRE L'A EXIGE / A INSISTE.......... 5<br>AUTRE__________ 6<br>(A PRECISER)<br>→ **Q412**	EVITER MALADIES.......... 1<br>EVITER GROSSESSE.......... 2<br>EVITER A LA FOIS VIH/IST.......... 3<br>N'A PAS CONFIANCE A LA PARTENAIRE.......... 4<br>LA PARTENAIRE L'A EXIGE / A INSISTE.......... 5<br>AUTRE__________ 6<br>(A PRECISER)<br>→ **Q412**	EVITER MALADIES.......... 1<br>EVITER GROSSESSE.......... 2<br>EVITER A LA FOIS VIH/IST.......... 3<br>N'A PAS CONFIANCE A LA PARTENAIRE.......... 4<br>LA PARTENAIRE L'A EXIGE / A INSISTE.......... 5<br>AUTRE__________ 6<br>(A PRECISER)	→ **Q412**
Q411	Quelle est la principale raison pour laquelle vous n'avez pas utilisé une méthode (OU NE SAIT PAS QU'UNE METHODE A ÉTÉ UTILISE)?	PARTENAIRE OCCASIONNELLE N'AS PAS FAIT ATTENTION.......... 11<br>LA CONTRACEPTION EST UNE AFFAIRE DE FEMME.......... 12<br><br>FEMME MENOPAUSEE.......... 21<br>COUPLE STERILE.......... 22<br>FEMME ENCEINTE.......... 23<br>FEMME AMENORRHEIQUE.......... 24<br>FEMME ALLAITANTE.......... 25<br>DESIR D'ENFANT.......... 26<br><br>ENQUÊTE OPPOSE A LA PF.......... 31<br>FEMME OPPOSEE A LA PF.......... 32<br>LES AUTRES S'OPPOSENT.......... 33<br>INTERDICTION RELIGIEUSE.......... 34<br><br>NE CONNAIT PAS DE METHODE.......... 41<br>NE CONNAIT PAS D'ENDROIT.......... 42<br><br>PROBLEMES DE SANTE.......... 51<br>CRAINT EFFETS SECONDAIRES.......... 52<br>PROBLEME D'ACCES/TROP LOIN.......... 53<br>COÛT TROP ELEVE.......... 54<br>PAS FACILE A UTILISER.......... 55<br>INFLUENCE LE PROCESSUS NORMAL DE CROISSANCE.......... 56<br><br>AUTRE__________ 96<br>(A PRECISER)<br>NSP.......... 98	PARTENAIRE OCCASIONNELLE N'AS PAS FAIT ATTENTION... 11<br>LA CONTRACEPTION EST UNE AFFAIRE DE FEMME.......... 12<br><br>FEMME MENOPAUSEE.......... 21<br>COUPLE STERILE.......... 22<br>FEMME ENCEINTE.......... 23<br>FEMME AMENORRHEIQUE.......... 24<br>FEMME ALLAITANTE.......... 25<br>DESIR D'ENFANT.......... 26<br><br>ENQUÊTE OPPOSE A LA PF.......... 31<br>FEMME OPPOSEE A LA PF.......... 32<br>LES AUTRES S'OPPOSENT.......... 33<br>INTERDICTION RELIGIEUSE.......... 34<br><br>NE CONNAIT PAS DE METHODE 41<br>NE CONNAIT PAS D'ENDROIT... 42<br><br>PROBLEMES DE SANTE.......... 51<br>CRAINT EFFETS SECONDAIRES 52<br>PROBLME D'ACCES/TROP LOIN 53<br>COÛT TROP ELEVE.......... 54<br>PAS FACILE A UTILISER.......... 55<br>INFLUENCE LE PROCESSUS NORMAL DE CROISSANCE... 56<br><br>AUTRE__________ 96<br>(A PRECISER)<br>NSP.......... 98	PARTENAIRE OCCASIONNELLE N'AS PAS FAIT ATTENTION.......... 11<br>LA CONTRACEPTION EST UNE AFFAIRE DE FEMME.......... 12<br><br>FEMME MENOPAUSEE.......... 21<br>COUPLE STERILE.......... 22<br>FEMME ENCEINTE.......... 23<br>FEMME AMENORRHEIQUE.......... 24<br>FEMME ALLAITANTE.......... 25<br>DESIR D'ENFANT.......... 26<br><br>ENQUÊTE OPPOSE A LA PF.......... 31<br>FEMME OPPOSEE A LA PF.......... 32<br>LES AUTRES S'OPPOSENT.......... 33<br>INTERDICTION RELIGIEUSE.......... 34<br><br>NE CONNAIT PAS DE METHODE.......... 41<br>NE CONNAIT PAS D'ENDROIT.......... 42<br><br>PROBLEMES DE SANTE.......... 51<br>CRAINT EFFETS SECONDAIRES.......... 52<br>PROBLEME D'ACCES/TROP LOIN..... 53<br>COÛT TROP ELEVE.......... 54<br>PAS FACILE A UTILISER.......... 55<br>INFLUENCE LE PROCESSUS NORMAL DE CROISSANCE.......... 56<br><br>AUTRE__________ 96<br>(A PRE (A PRECISER)<br>NSP.......... 98	
Q412	Quelle est votre lien/relation avec cette femme?<br><br>SI LA FEMME EST "FIANCÉE" OU "PETITE AMIE", DEMANDEZ:<br>Votre fiancée ou petite amie vivait-elle avec vous quand vous aviez eu votre dernier rapport sexuel?<br><br>SI LA REPONSE EST "OUI", REPORTEZ "01" ("EPOUSE"); SI "NON", REPORTEZ "02" (FIANCEE) OU "03" (PETITE AMIE)	EPOUSE/CONCUBINE.......... 01<br>FIANCÉE.......... 02<br>PETITE AMIE .... 03<br>AUTRE AMIE .... 04<br>PARTENAIRE OCCASIONNELLE.......... 05<br>PARTENAIRE COMMERCIALE / TRAVAILLEUSE DE SEXE.......... 06<br>AUTRE__________ 96<br>(A PRECISER)	EPOUSE/CONCUBINE.......... 01<br>FIANCÉE.......... 02<br>PETITE AMIE .... 03<br>AUTRE AMIE .... 04<br>PARTENAIRE OCCASIONNELLE... 05<br>PARTENAIRE COMMERCIALE / TRAVAILLEUSE DE SEXE... 06<br>AUTRE__________ 96<br>(A PRECISER)	EPOUSE/CONCUBINE.......... 01<br>FIANCÉE.......... 02<br>PETITE AMIE .... 03<br>AUTRE AMIE .... 04<br>PARTENAIRE OCCASIONNELLE.......... 05<br>PARTENAIRE COMMERCIALE / TRAVAILLEUSE DE SEXE.......... 06<br>AUTRE__________ 96<br>(A PRECISER)	
Q413	Quelle a été la durée de la relation avec cette femme?<br><br>SI A EU RAPPORT SEXUEL UNE SEULE FOIS AVEC CETTE FEMME, NOTEZ "01"JOURS.	JOURS.......... 1<br>SEMAINES.... 2<br>MOIS............ 3<br>ANNEES....... 4<br>☐☐	JOURS..... 1<br>SEMAINES 2<br>MOIS........ 3<br>ANNEES... 4<br>☐☐	JOURS..... 1<br>SEMAINES 2<br>MOIS....... 3<br>ANNEES... 4<br>☐☐	
Q413b	A part cette personne, avez vous eu des rapports sexuels avec une autre personne au cours des 12 derniers mois?<br><br>OUI................... 1 Retourner à Q406<br>NON................... 2 → **Q414**		A part ces deux partenaires avez vous eu des rapports sexuels avec d'autre personne au cours des 12 derniers mois?<br>OUI......1 Retourner à Q406<br>Non......2 → **Q414**		

Q414	Au total, avec combien de femmes avez-vous eu un rapport sexuel au cours des 12 derniers mois?<br><br>ASSUREZ-VOUS DE LA COHERENCE ENTRE LE NOMBRE DE PARTENAIRES MENTIONNES ICI ET LE TABLEAU PRECEDENT	NOMBRE DE FEMMES……………………………………… [ ][ ]<br>PLUS DE 95 FEMMES ……………………………………… 95	
	Maintenant, je voudrais vous poser des questions sur votre vie conjugale. Rappelez-vous que vos réponses sont confidentielles		
Q415	Avez-vous déjà été marié ou avez-vous déjà vécu avec une femme ?	OUI,A ETE MARIE……………………………………………… 1<br>OUI, A VECU AVEC UNE FEMME……………………………… 2<br>NON ……………………………………………………………… 3	3 → Q501
Q416	Quel est votre état matrimonial actuel. Êtes-vous actuellement marié, ou vivez vous avec une femme, veuf, divorcé ou séparé ?	ACTUELLEMENT MARIE……………………………………… 1<br>VIT AVEC UNE FEMME……………………………………… 2<br>VEUF……………………………………………………… 3<br>DIVORCE…………………………………………………… 4<br>SEPARE……………………………………………………… 5	3, 4, 5 → Q420
Q417	Votre/vos femme (s)/partenaire(s) vit/vivent-elle(s) actuellement avec vous, où habite(ent)-elle(s) ailleurs?	VIT AVEC PARTENAIRE/EPOUSE……………………………… 1<br>EPOUSE/PARTENAIRE HABITE AILLEURS……………………… 2	
Q418	Avez-vous plus d'une épouse ou femme avec qui vous vivez maritalement ?	OUI………………………………………………………… 1<br>NON………………………………………………………… 2	2 → Q420
Q419	En tout, avec combien d'épouses ou d'autres partenaires vivez-vous de manière maritale (ou comme si vous êtiez marié)?	NOMBRE TOTAL D'EPOUSES/<br>DE PARTENAIRES:……………….. [ ][ ]	
Q420	Avez-vous été marié une seule fois ou plus d'une fois/ avez-vous vécu avec une seule femme ou avec plus d'une femme?	SEULEMENT UNE FOIS……………………………………… 1<br>PLUS D'UNE FOIS ……………………………………… 2	
Q421	***VERIFIEZ Q420:***<br>A ÉTÉ MARIE UNE SEULE FOIS/A VECU AVEC UNE SEULE FEMME [ ] ↓ En quel mois et en quelle année avez-vous commencé à vivre avec votre femme?<br>A ÉTÉ MARIE PLUS D'UNE FOIS/A VECU AVEC PLUS D'UNE FEMME [ ] ↓ En quel mois et année aviez-vous commencé à vivre avec votre première partenaire ou épouse?	MOIS…………………………………………… [ ][ ]<br>NE CONNAIT PAS LE MOIS ……………………… 98<br>ANNEE…………………………………… [ ][ ][ ][ ]<br>NE CONNAIT PAS L'ANNEE ……………………… 9998	

SECTION 5: PREFERENCES EN MATIERE DE FECONDITE			
	**Questions et filtres**	**Modalités de codage**	**Allez à**
Q501	**VERIFIEZ Q416 & Q415 = STATUT MARITAL**<br>ACTUELLEMENT MARIE OU VIT EN UNION (Q416=1 OU 2) ☐ ↓	PAS EN UNION (Q415=3 OU Q416=3, 4 OU 5) ☐ →	**Q504**
Q502	**VERIFIEZ 302 : (02) ; STERILISATION MASCULINE**<br>HOMME **PAS** STERILISE ☐ ↓	HOMME STERILISE ☐ →	**Q506**
Q503	Votre épouse/partenaire (ou une de vos épouses/ partenaires) est-elle actuellement enceinte ?	OUI .... 1<br>NON.. 2<br>NE SAIT PAS 8	
Q504	**VERIFIEZ Q501 ET Q503:**<br>Maintenant, j'ai des questions concernant votre futur.<br>PAS D'EPOUSE OU PARTENAIRE /PARTENAIRE N'EST PASENCEINTE OU PAS SÛRE SI ELLE EST ENCEINTE ☐ ↓<br>Voudriez-vous avoir un (autre) enfant ou préfèreriez-vous ne pas d'(autres) enfants?<br>FEMME/PARTENAIRE ENCEINTE ☐ ↓<br>Après l'enfant que votre épouse/partenaire attend, voudriez-vous avoir un autre enfant, ou préfèreriez vous ne pas avoir d'autre enfant du tout?	AVOIR UN (UN AUTRE) ENFANT.................. 1<br>PLUS D'ENFANTS/AUCUN........................ 2<br>FEMME/FEMMES TOUTES INFECONDES / STERILISEES........................... 3<br>ENQUETE INFECOND.................................. 4<br>INDECIS / NE SAIT PAS.............................. 8	(2, 3, 4, 8) → **Q506**
Q505	**VERIFIEZ Q503**<br>PAS ENCEINTE OU PAS SURE D'ÊTRE ENCEINTE ☐ ↓<br>Combien de temps voudriez-vous attendre à partir de maintenant avant la naissance d'un (autre) enfant ?<br>ENCEINTE ☐ ↓<br>Après la naissance de l'enfant que vous attendez, combien de temps voudriez-vous attendre pour avoir un autre enfant ?<br>SI LA REPONSE EST UN NOMBRE DECIMAL DE MOIS OU D'ANNES, CONVERTIR LE TOUT EN MOIS ET REPORTER DANS LA CASE "MOIS" (PAR EXEMPLE, 2 1/2 ANS=30 MOIS)	MOIS..................................... 1 ☐☐<br>ANNÉES .... 2 ☐☐<br>BIENTÔT/MAINTENANT........................... 993<br>FEMME NE PEUT PAS AVOIR D'ENFANTS.. 994<br>APRÈS MARIAGE .... 995<br>AUTRE ______________________________ 996<br>(PRÉCISER)<br>NE SAIT PAS 998	→ **Q508**
Q505.1	ACTUELLEMENT MARIE VIT EN UNION (Q416=1 OU 2) ☐ ↓	PAS EN UNION (Q415=3 OU Q416=3, 4 OU 5) ☐ →	**Q508**
Q506	Combien d'enfants pensez-vous que votre femme (toutes vos femmes ensemble) voudrait(ent)-elle(s) avoir dans la vie ?<br>**SI AUCUN REPORTEZ "00"**	NOMBRE D'ENFANTS..................... ☐☐<br>NE SAIT PAS 98	98 → **Q507.1**
Q507	Diriez-vous que ....<br>Vous voudriez avoir le même nombre d'enfants qu'elle(s) ou plus ou bien moins d'enfants qu'elle(s) ?	MEME NOMBRE... 1<br>PLUS D'ENFANTS...................................... 2<br>MOINS D'ENFANTS.................................... 3<br>NE SAIT PAS 8	
Q507.1	Qui décide du nombre d'enfants que vous allez avoir -diriez-vous que cette décision vous appartient principalement, appartient principalement à votre femme/partenaire, ou avez-vous décidé tous les deux ensemble ?	PRINCIPALEMENT VOUS.................................. 1<br>PRINCIPALEMENT VOTRE PARTENAIRE............ 2<br>CONJOINTEMENT... 3<br>AUTRE___________________________________ 6<br>(PRECISER)	

Q508	**VERIFIEZ Q202 & Q204:**<br>**INSITEZ POUR AVOIR UNE REPONSE NUMERIQUE**<br>A DES ENFANTS EN VIE ☐ → Si vous pouviez retourner à l'époque où vous n'aviez pas encore d'enfants et où vous pouviez choisir exactement le nombre d'enfants à avoir dans toute votre vie, combien auriez-vous voulu en avoir?<br>PAS D'ENFANTS EN VIE ☐ → Si vous pouviez choisir exactement le nombre d'enfants que vous voulez toute votre vie, combien auriez-vous voulu?	AUCUN .... 00<br>NOMBRE...................................... ☐☐<br>AUTRE______________________________ 96<br>(PRECISEZ)	→ **Q510**<br><br>→ **Q510**
Q509	Parmi ces enfants, combien souhaiteriez-vous de garçons, combien souhaiteriez-vous de filles, et pour combien d'entre eux, le sexe n'aurait-il pas d'importance ?	GARçONS / FILLES / INDIFFERENT<br>NOMBRE ☐☐ ☐☐ ☐☐	
Q510	Seriez-vous prêt à utiliser une méthode de PF en vue d'avoir exactement ce nombre d'enfants ?	OUI .... 1<br>NON... 2	
	Maintenant je voudrais vous demandez vos opinions concernant la grossesse et la naissance.		
Q511	Selon vous, combien de temps un couple doit il attendre après le mariage pour avoir leur premier enfant?	IMMEDIATEMENT.. 1<br>MOINS D'UN AN.. 2<br>ENTRE UN AN ET AVANT 2 ANS APRES MARIAGE.. 3<br>2 OU PLUSIEURS ANNEES.................................. 4<br>AUTRE________________________________ 6<br>(PRECISER)	
Q512	Selon vous, quel devrait être l'âge idéal en années pour une femme de tomber enceinte ou avoir un enfant pour la première fois ?	ÂGE EN ANNEES .... ☐☐<br>NE SAIT PAS 98	
Q513	Selon vous, quel devrait être l'âge idéal en années pour un homme d'être pour la première fois pére d'un enfant ?	ÂGE EN ANNEES .... ☐☐<br>NE SAIT PAS 98	
Q514	A votre avis, quel devrait être l'intervalle d'âge idéal entre deux enfants?<br>SI LA REPONSE EST UN NOMBRE DECIMAL DE MOIS OU D'ANNES, CONVERTIR LE TOUT EN MOIS ET REPORTER DANS LA CASE "MOIS" (PAR EXEMPLE, 2 1/2 ANS=30 MOIS)	MOIS..................................... 1 ☐☐<br>OU<br>ANNEES................................. 2 ☐☐<br>NE SAIT PAS 998	
Q515	Quels sont les effets (positifs et/ou négatifs) sur la mère si elle utilise une méthode de PF ou d'espacement des naissances ?<br><br>INSISTER: D'autres effets?<br><br>ENREGISTRER TOUT CE QUI EST MENTIONNE.	MEILLEUR ETAT NUTRITIONNEL.................... A<br>INCIDENCE D'ANEMIE PLUS FAIBLE.............. B<br>MOINS DE COMPLICATIONS DE GROSSESSE C<br>PREVENIR IST /VIH...................................... D<br>PREVENIR GROSSESSES INDESIRABLES...... E<br>MOINS D'ENFANT A EDUQUER...................... F<br>LA FEMME A PLUS DE TEMPS LIBRE............. G<br>LA FAMILLE GAGNE PLUS D'ARGENT............ H<br>ESPRIT TRANQUILLE..................................... I<br>BONHEUR FAMILIAL /ENTENTE DANS LE COUPLE.. J<br>MEILLEURE SANTE DE LA MERE................... K<br>PROBLEMES DE COUPLE/DISPUTES/DIVORCI L<br>REJET COMMUNAUTE/PROCHES.................. M<br>PROBLEMES DE CYCLE................................ N<br>PROBLEMES DE SANTE................................ O<br>GAIN DE POIDS... P<br>PERTE DE POIDS... Q<br>AFFAIBLISSEMENT....................................... R<br>RETOUR TARDIF DE FECONDITE S<br>AUTRE________________________________ X<br>(PRECISER)<br>AUCUN... Y	

Q516	Quels sont les effets (positifs/négatifs) sur l'enfant si la mère utilise une méthode de PF ou d'espacement des naissances ?<br><br>INSISTER: D'autres effets?<br><br>NE PAS LIRE LES REPONSES.	MEILLEURE CROISSANCE............................ A<br>MEILLEUR ETAT NUTRITIONNEL.................. B<br>MEILLEURE SANTE...................................... C<br>MEILLEURE CHANCE DE SURVIE.................. D<br>PLUS GRANDE ATTENTION DE LA MERE....... E<br>MEILLEURE EDUCATION.............................. F<br>PLUS FAIBLE INCIDENCE DES MALADIES...... G<br>PLUS D'OPPORTUNITES DANS LA VIE........... H<br>MALFORMATIONS.. I<br>PROBLEMES DE CROISSANCE..................... J<br>OBESITE.. K<br>PROBLEMES NUTRITIONNELS..................... L<br>MALADIES FREQUENTES............................. M<br>NAISSANCES MULTIPLES/JUMEAUX.............. N<br>AUTRE_________________________________ X<br>(PRECISER)<br>AUCUN.. Y	
Q517	**VERIFIEZ Q415 ET Q416: DÉJÀ MARIE ET ACTUELLEMENT MARIE**<br>ACTUELLEMENT MARIE (Q416=1 OU 2) ☐ ↓	PAS EN UNION (Q415=3 OU (Q416=3, 4 OU 5) ☐ →	**Q601**
Q518	Avez-vous jamais accompagné votre épouse (ou votre enfant) dans une structure de santé ?	OUI .... 1<br>NON.. 2	→ **Q601**
Q519	A quelle fréquence accompagnez-vous votre épouse (ou enfant) dans une structure sanitaire ?	RAREMENT.. 1<br>QUELQUEFOIS... 2<br>SOUVENT... 3<br>TOUJOURS... 4	

**SECTION 6: EXPOSITION AUX MEDIA ET COMMUNICATION INTERPERSONNELLE**			
	**Questions et filtres**	**Modalités de codage**	**Sauts**
Maintenant, je voudrais vous parler de vos besoins en informations et des endroits où obtenir des informations sur la planification familiale.			
Q601	Quelles sont vos principales sources d'informations sur la santé ?<br><br>**INSISTER SEPAREMENT POUR :**<br>A. SOURCES MEDIATIQUES<br>B. SOURCES MEDICALES<br>C. SOURCES COMMUNAUTAIRES<br>D. SOURCES INTERPERSONNELLES<br><br>ENREGISTRER TOUT CE QUI EST MENTIONNE.	**SOURCES MEDIATIQUES**<br>RADIO.......... AA<br>TV.......... AB<br>JOURNAUX.......... AC<br>MAGAZINES.......... AD<br>PANNEAUX D'AFFICHAGE.......... AE<br>PEINTURE MURALE.......... AF<br>INTERNET .... AG<br><br>**SOURCES MEDICALES**<br>MEDECIN.......... BA<br>SAGE-FEMME.......... BB<br>INFIRMIER/AI.......... BC<br>MATRONE / ASC .... BD<br>GUERISSEUR / ACCOUCHEUSE TRADITIONNELLE....... BE<br>PHARMACIEN .... BF<br><br>**SOURCES COMMUNAUTAIRES**<br>RELAI COMMUNAUTAIRE .... CA<br>ORGANISATION COMMUNAUTAIRE DE BASE (OCB)...... CB<br>VOLONTAIRES / PAIRS ÉDUCATEURS .... CC<br>ECOLE.......... CD<br>TRAVAILLEUR D'ONG.......... CE<br><br>**SOURCES INTERPERSONNELLES**<br>PARENTS.......... DA<br>BEAUX-PARENTS.......... DB<br>EPOUX/PARTENAIRE.......... DC<br>FRERES ET SŒURS.......... DD<br>BELLES SŒURS/BEAUX-FRERES.......... DE<br>AMIS / VOISINS.......... DF<br>AUTRES PARENTS/CONNAISSANCES.......... DG<br><br>**AUTRE (PRECISER)**__________ XX<br>**AUCUNE..........** YY<br>**NE SAIT PAS ....** ZZ	
Q602	Lisez-vous des journaux ?	OUI .... 1<br>NON 2	 → **Q604**
Q603	Quel(s) journal (ux) lisez-vous le plus souvent?<br><br>INSISTER: D'autres journaux?<br><br>ENREGISTRER TOUT CE QUI EST MENTIONNE.	L'OBSERVATEUR.......... A<br>LE POPULAIRE.......... B<br>LE QUOTIDIEN.......... C<br>LE SOLEIL.......... D<br>SUD QUOTIDIEN.......... E<br>WALF QUOTIDIEN.......... F<br>L'AS.......... G<br>KOTCH.......... H<br>LE MESSAGER.......... I<br>WALF GRAND PLACE.......... J<br>LE MATIN.......... K<br>AUTRE JOURNAL __________ X<br>(A PRECISER)	
Q604	Lisez-vous des magazines?	OUI .... 1<br>NON 2	 → **Q606**
Q605	Quel(s) magazine(s) lisez-vous le plus souvent ?<br><br>INSISTER: D'autres magazines?<br><br>ENREGISTRER TOUT CE QUI EST MENTIONNE.	WEEK-END.......... A<br>THIOF.......... B<br>LISSA.......... C<br>ICONE.......... D<br>DAKAR LIFE.......... E<br>LIFA.......... F<br>AUTRE __________ X<br>(A PRECISER)	

Q606	**VERIFIEZ: Q602 & 604**<br>**SI OUI A L'UNE OU L'AUTRE** (Q602=1 OU Q604=1) ☐<br>**SI NON AUX DEUX** (Q602=2 ET Q604=2) ☐		**Q609**
Q607	Avez-vous lues des informations sur la PF dans les journaux/magazines au cours des trois derniers mois ?	OUI .... 1<br>NON 2	 **Q609**
Q608	Quelles informations avez-vous lues dans les journaux/magazines sur la planification familiale ?<br><br>INSISTER: D'autres informations?<br><br>ENREGISTRER TOUT CE QUI EST MENTIONNE.<br><br>SI PILULE, INSISTER POUR SAVOIR SI PILULE JOURNALIERE OU CONTRACEPTION D'URGENCE.	PILULES.......... A<br>DIU.......... B<br>PRESERVATIFS.......... C<br>INJECTABLES .... D<br>IMPLANT.......... E<br>PILULES DU LENDEMAIN.......... F<br>STERILISATION FEMININE.......... G<br>STERILISATION MASCULINE.......... H<br>ALLAITEMENT MATERNEL.......... I<br>METHODE DES JOURS FIXES.......... J<br>AGE DU MARIAGE.......... K<br>RETARDER L'AGE DU 1[ER] RAPPORT SEXUEL.......... L<br>RETARDER LA PREMIERE NAISSANCE.......... M<br>ESPACEMENT DES NAISSANCES.......... N<br>LIMITATION DE LA TAILLE DE LA FAMILLE.......... O<br>AUTRES__________ X<br>(PRECISER)	
Q609	Ecoutez-vous la radio?	OUI .... 1<br>NON 2	 **Q613**
Q610	Quelles stations de radio écoutez-vous?<br><br>INSISTER: D'autres radios?<br><br>ENREGISTRER TOUT CE QUI EST MENTIONNE.	RTS.......... A<br>WALF FM.......... B<br>SUD FM.......... C<br>DUNYA FM.......... D<br>RFM.......... E<br>ZIK FM.......... F<br>OXYJEUNES.......... G<br>X-FM.......... H<br>ALFAIDA.......... I<br>NDEFLENG.......... J<br>SOXNA FM.......... K<br>DJIDA FM.......... L<br>LA COTIERE.......... M<br>RFI.......... N<br>AUTRE__________ X<br>(PRECISEZ)	
Q611	Avez-vous entendu à la radio des informations sur la PF au cours des trois derniers mois?	OUI .... 1<br>NON 2	 **Q613**

Q612	Quelles informations avez-vous entendues à la radio sur la planification familiale?<br><br>INSISTER: D'autres informations?<br><br>ENREGISTRER TOUT CE QUI EST MENTIONNE.<br><br>SI PILULE, INSISTER POUR SAVOIR SI PILULE JOURNALIERE OU CONTRACEPTION D'URGENCE.	PILULES…… A<br>DIU…… B<br>PRESERVATIFS…… C<br>INJECTABLES …… D<br>IMPLANT…… E<br>PILULES DU LENDEMAIN…… F<br>STERILISATION FEMININE…… G<br>STERILISATION MASCULINE…… H<br>ALLAITEMENT MATERNEL…… I<br>METHODE DES JOURS FIXES…… J<br>AGE DU MARIAGE…… K<br>RETARDER L'AGE DU 1[ER] RAPPORT SEXUEL…… L<br>RETARDER LA PREMIERE NAISSANCE…… M<br>ESPACEMENT DES NAISSANCES…… N<br>LIMITATION DE LA TAILLE DE LA FAMILLE…… O<br>AUTRES______ X<br>(PRECISER)	
Q613	A votre avis, les messages à la radio sur les thèmes suivants sont-ils acceptables ou non ?<br><br>a. Planification familial/espacement des naissances<br>b. VIH/SIDA<br>c. Santé maternelle (soins prenatals, acouchement,…)<br>d. Santé infantile (vaccination, soins préventifs,…)<br>e. Santé de reproduction (ISTs, infécondité)	ACCEPTABLE / INNACCEPTABLE<br>a 1 2<br>b 1 2<br>c 1 2<br>d 1 2<br>e 1 2	
Q614	Regardez-vous la télévision?	OUI…… 1<br>NON…… 2	 → **Q618**
Q615	Quelles chaînes regardez-vous généralement à la télévision?<br><br>INSISTER: D'autres chianes de télévision?<br><br>ENREGISTRER TOUT CE QUI EST MENTIONNE.	RTS 1 …… A<br>WALF TV…… B<br>SN2 …… C<br>RDV …… D<br>2STV …… E<br>AFRICABLE …… F<br>CANAL INFO …… G<br>CANAL HORIZONS …… H<br>TFM …… I<br>AUTRES______ X<br>((PRECISEZ)	
Q616	Avez-vous regardé à la télé des informations sur la PF au cours des trois derniers mois?	OUI …… 1<br>NON …… 2	 → **Q618**
Q617	Quelles informations avez-vous suivies à la télé sur la planification familiale?<br><br>INSISTER: D'autres informations?<br><br>ENREGISTRER TOUT CE QUI EST MENTIONNE.<br><br>SI PILULE, INSISTER POUR SAVOIR SI PILULE JOURNALIERE OU CONTRACEPTION D'URGENCE.	PILULES…… A<br>DIU…… B<br>PRESERVATIFS…… C<br>INJECTABLES …… D<br>IMPLANT…… E<br>PILULES DU LENDEMAIN…… F<br>STERILISATION FEMININE…… G<br>STERILISATION MASCULINE…… H<br>ALLAITEMENT MATERNEL…… I<br>METHODE DES JOURS FIXES…… J<br>AGE DU MARIAGE…… K<br>RETARDER L'AGE DU 1[ER] RAPPORT SEXUEL…… L<br>RETARDER LA PREMIERE NAISSANCE…… M<br>ESPACEMENT DES NAISSANCES…… N<br>LIMITATION DE LA TAILLE DE LA FAMILLE…… O<br>AUTRES______ X<br>(PRECISER)	

Q618	A votre avis, les messages à la télé sur les thèmes suivants sont-ils acceptables ou non ?<br>a. Planification familial/espacement des naissances<br>b.VIH/SIDA<br>c. Santé maternelle (soins prenatals, acouchement,…)<br>d. Santé infantile (vaccination, soins préventifs,…)<br>e. Santé de reproduction (ISTs, infécondité)	ACCEPTABLE / INNACCEPTABLE<br>a 1 2<br>b 1 2<br>c 1 2<br>d 1 2<br>e 1 2	
Q622	Avez-vous accès au téléphone portable?	OUI …… 1<br>NON …… 2<br>NE CONNAIT PAS TELEPHONE PORTABLE…… 8	 →Q627<br>→Q627
Q623	Avez-vous personnellement un téléphone portable à votre propre usage ?	OUI …… 1<br>NON …… 2	
Q624	Seriez-vous à l'aise en recevant des messages sur la PF /Contraception ou la santé par SMS ?	OUI …… 1<br>NON …… 2	
Q625	Au cours des 3 derniers mois, avez-vous reçu des messages SMS sur la PF/Contraception ?	OUI …… 1<br>NON …… 2	
Q626	Au cours des 3 derniers mois, avez-vous accédé à internet,web, ou email au moins une fois par téléphone portable?	OUI …… 1<br>NON …… 2<br>NE CONNAIT PAS INTERNET…… 8	 <br>→Q628
Q627	Au cours des 3 derniers mois, avez-vous accédé à internet,web, ou email au moins une fois par ordinateur?	OUI …… 1<br>NON …… 2	
Q628	Etes-vous membre d'une association, un groupe, une organisation?	OUI …… 1<br>NON …… 2	 →Q636

Q629<br>Quels sont les noms et types d'organisations dont vous êtes membre ?	Q630<br>Depuis combien de temps êtes-vous membre ?	Q631<br>A quelle périodicité cette organisation se réunit-elle ?	Q632<br>A quelle fréquence discutez-vous avec des membres de cette organisation en dehors de vos rencontres au sein de l'organisation ?	Q633<br>A quelle fréquence assistez-vous aux réunions de l'organisation ?	Q634<br>Avez-vous jamais vu ou entendu des informations sur la PF au cours des rencontres de l'organisation ?	Q635<br>Quelles informations sur la PF avez-vous vu/entendu au cours des rencontres de l'organisation ?
1)<br>____________________<br>NOM ORGANISATION<br>____________________<br>TYPE D'ORGANISATION<br>(Association professionnelle, Organisation sportive, Groupe de théâtre, Association religieuse, etc….)<br>[ ][ ]<br>A CODIFIER AU BUREAU	< 1 ANS …… 1<br>1 - 2 ANS …. 2<br>3 - 5 ANS …. 3<br>6 ANS ET PL 4<br>NSP………… 8	CHAQUE JOUR……… 1<br>CHAQUE SEM………. 2<br>CHAQUE MOIS ……… 3<br>CHAQUE ANNÉE …… 4<br>PAS RÉGULIER …….. 5<br>AUTRES __________ 6<br>(PRECISER)	CHAQUE JOUR……… 1<br>CHAQUE SEM………. 2<br>CHAQUE MOIS ……… 3<br>CHAQUE ANNÉE …… 4<br>PAS RÉGULIER …….. 5<br>AUTRES __________ 6<br>(PRECISER)	TOUJOURS…… 1<br>SOUVENT…….. 2<br>PARFOIS………. 3<br>RAREMENT…… 4<br>JAMAIS………… 5	OUI …………….. 1<br>NON……………. 2<br>LIGNE SUIV. ←<br>NSP……………. 8	PILULES……………………………………… A<br>DIU………………………………………… B<br>PRESERVATIFS…………………………… C<br>INJECTABLES …………………………… D<br>IMPLANT…………………………………… E<br>PILULES DU LENDEMAIN……………… F<br>STERILISATION FEMININE…………… G<br>STERILISATION MASCULINE………… H<br>METHODE DES JOURS FIXES………… I<br>AGE DU MARIAGE…………………… J<br>RETARDER L'AGE DU 1ER RAPPORT SEXUEL.. K<br>RETARDER LA PREMIERE NAISSANCE………. L<br>ESPACEMENT DES NAISSANCES……………… M<br>LIMITATION DE LA TAILLE DE LA FAMILLE…… N<br>AUTRES ____________________________ X<br>(PRECISER)
2)<br>____________________<br>NOM ORGANISATION<br>____________________<br>TYPE D'ORGANISATION<br>TYPE D'ORGANISATION<br>(Association professionnelle, Organisation sportive, Groupe de théâtre, Association religieuse, etc….)<br>[ ][ ]<br>A CODIFIER AU BUREAU	< 1 ANS …… 1<br>1 - 2 ANS …. 2<br>3 - 5 ANS …. 3<br>6 ANS ET PL 4<br>NSP………… 8	CHAQUE JOUR……… 1<br>CHAQUE SEM………. 2<br>CHAQUE MOIS ……… 3<br>CHAQUE ANNÉE …… 4<br>PAS RÉGULIER …….. 5<br>AUTRES __________ 6<br>(PRECISER)	CHAQUE JOUR……… 1<br>CHAQUE SEM………. 2<br>CHAQUE MOIS ……… 3<br>CHAQUE ANNÉE …… 4<br>PAS RÉGULIER …….. 5<br>AUTRES __________ 6<br>(PRECISER)	TOUJOURS…… 1<br>SOUVENT…….. 2<br>PARFOIS………. 3<br>RAREMENT…… 4<br>JAMAIS………… 5	OUI …………….. 1<br>NON……………. 2<br>LIGNE SUIV. ←<br>NSP……………. 8	PILULES……………………………………… A<br>DIU………………………………………… B<br>PRESERVATIFS…………………………… C<br>INJECTABLES …………………………… D<br>IMPLANT…………………………………… E<br>PILULES DU LENDEMAIN……………… F<br>STERILISATION FEMININE…………… G<br>STERILISATION MASCULINE………… H<br>METHODE DES JOURS FIXES………… I<br>AGE DU MARIAGE…………………… J<br>RETARDER L'AGE DU 1ER RAPPORT SEXUEL.. K<br>RETARDER LA PREMIERE NAISSANCE………. L<br>ESPACEMENT DES NAISSANCES……………… M<br>LIMITATION DE LA TAILLE DE LA FAMILLE…… N<br>AUTRES ____________________________ X<br>(PRECISER)

Q629<br>Quels sont les noms et types d'organisations dont vous êtes membre ?	Q630<br>Depuis combien de temps êtes-vous membre ?	Q631<br>A quelle périodicité cette organisation se réunit-elle ?	Q632<br>A quelle fréquence discutez-vous avec des membres de cette organisation en dehors de vos rencontres au sein de l'organisation ?	Q633<br>A quelle fréquence assistez-vous aux réunions de l'organisation ?	Q634<br>Avez-vous jamais vu ou entendu des informations sur la PF au cours des rencontres de l'organisation ?	Q635<br>Quelles informations sur la PF avez-vous vu/entendu au cours des rencontres de l'organisation ?
3)<br>________________<br>NOM ORGANISATION<br><br>________________<br>TYPE D'ORGANISATION<br>TYPE D'ORGANISATION<br>(Association professionnelle, Organisation sportive, Groupe de théâtre, Association religieuse, etc….)<br><br>[ ][ ]<br>A CODIFIER AU BUREAU	< 1 ANS …… 1<br>1 - 2 ANS …. 2<br>3 - 5 ANS …. 3<br>6 ANS ET PL 4<br>NSP………… 8	CHAQUE JOUR……… 1<br>CHAQUE SEM……….. 2<br>CHAQUE MOIS ……… 3<br>CHAQUE ANNÉE …… 4<br>PAS RÉGULIER …….. 5<br>AUTRES __________ 6<br>(PRECISER)	CHAQUE JOUR……… 1<br>CHAQUE SEM……….. 2<br>CHAQUE MOIS ……… 3<br>CHAQUE ANNÉE …… 4<br>PAS RÉGULIER …….. 5<br>AUTRES __________ 6<br>(PRECISER)	TOUJOURS…… 1<br>SOUVENT…….. 2<br>PARFOIS………. 3<br>RAREMENT…… 4<br>JAMAIS………… 5	OUI ……………. 1<br>NON……………. 2<br>LIGNE SUIV. ←<br>NSP……………. 8	PILULES…………………………………… A<br>DIU……………………………………… B<br>PRESERVATIFS……………………………… C<br>INJECTABLES ……………………………… D<br>IMPLANT…………………………………… E<br>PILULES DU LENDEMAIN………………… F<br>STERILISATION FEMININE……………… G<br>STERILISATION MASCULINE…………… H<br>METHODE DES JOURS FIXES…………… I<br>AGE DU MARIAGE…………………………… J<br>RETARDER L'AGE DU 1ER RAPPORT SEXUEL.. K<br>RETARDER LA PREMIERE NAISSANCE………. L<br>ESPACEMENT DES NAISSANCES……………. M<br>LIMITATION DE LA TAILLE DE LA FAMILLE…… N<br>AUTRES ____________________ X<br>(PRECISER)
4)<br>________________<br>NOM ORGANISATION<br><br>________________<br>TYPE D'ORGANISATION<br>TYPE D'ORGANISATION<br>(Association professionnelle, Organisation sportive, Groupe de théâtre, Association religieuse, etc….)<br><br>[ ][ ]<br>A CODIFIER AU BUREAU	< 1 ANS …… 1<br>1 - 2 ANS …. 2<br>3 - 5 ANS …. 3<br>6 ANS ET PL 4<br>NSP………… 8	CHAQUE JOUR……… 1<br>CHAQUE SEM……….. 2<br>CHAQUE MOIS ……… 3<br>CHAQUE ANNÉE …… 4<br>PAS RÉGULIER …….. 5<br>AUTRES __________ 6<br>(PRECISER)	CHAQUE JOUR……… 1<br>CHAQUE SEM……….. 2<br>CHAQUE MOIS ……… 3<br>CHAQUE ANNÉE …… 4<br>PAS RÉGULIER …….. 5<br>AUTRES __________ 6<br>(PRECISER)	TOUJOURS…… 1<br>SOUVENT…….. 2<br>PARFOIS………. 3<br>RAREMENT…… 4<br>JAMAIS………… 5	OUI ……………. 1<br>NON……………. 2<br>LIGNE SUIV. ←<br>NSP……………. 8	PILULES…………………………………… A<br>DIU……………………………………… B<br>PRESERVATIFS……………………………… C<br>INJECTABLES ……………………………… D<br>IMPLANT…………………………………… E<br>PILULES DU LENDEMAIN………………… F<br>STERILISATION FEMININE……………… G<br>STERILISATION MASCULINE…………… H<br>METHODE DES JOURS FIXES…………… I<br>AGE DU MARIAGE…………………………… J<br>RETARDER L'AGE DU 1ER RAPPORT SEXUEL.. K<br>RETARDER LA PREMIERE NAISSANCE………. L<br>ESPACEMENT DES NAISSANCES……………. M<br>LIMITATION DE LA TAILLE DE LA FAMILLE…… N<br>AUTRES ____________________ X<br>(PRECISER)

Q636	*Je voudrais vous demander quelques questions sur les discussions que vous auriez eues avec d'autres personnes au sujet des problemes de santé.*<br>VERIFIEZ Q415 & Q416:<br>MARIE OU VIT MARITALEMENT AVEC UNE FEMME (Q416=1 OU 2) ☐ ↓	PAS EN UNION ☐ (Q415=3 OU Q416=3, 4 OU 5)	→ Q644
Q637	Avez-vous, vous et votre épouse/partenaire, déjà discuté du nombre d'enfants que vous aimeriez avoir ?	OUI .... 1<br>NON 2	2 → Q639
Q638	Quelle est la fréquence avec laquelle vous avez parlé à votre épouse/partenaire de ce sujet au cours des 6 derniers mois?	N'AVONS PAS DISCUTE AU COURS DES SIX DERNIERS MOIS.......... 1<br>UNE OU DEUX FOIS.......... 2<br>PLUS DE DEUX FOIS.......... 3<br>SOUVENT/REGULIEREMENT.......... 4<br>AUTRES________________ 6<br>(PRECISER)	
Q639	Avez-vous déjà discuté de PF/l'utilisation de la contraception avec votre épouse/partenaire ?	OUI .... 1<br>NON 2	2 → Q643
Q640	Quelle est la fréquence avec laquelle vous avez parlé à votre épouse/partenaire de ce sujet au cours des 6 derniers mois?	N'AVONS PAS DISCUTE AU COURS DES SIX DERNIERS MOIS.......... 1<br>UNE OU DEUX FOIS.......... 2<br>PLUS DE DEUX FOIS.......... 3<br>SOUVENT/REGULIEREMENT.......... 4<br>AUTRES________________ 6<br>(PRECISER)	
Q641	D'habitude, qui d'entre-vous commence la discussion sur la PF, vous ou votre épouse/partenaire ?	MOI-MÊME.......... 1<br>EPOUX/PARTENAIRE.......... 2<br>L'UN OU L'AUTRE.......... 3	
Q642	Quel est le degré de difficulté à commencer une conversation sur l'espacement des naissances avec votre partenaire – très difficile, quelque peu difficile, facile ?	TRES DIFFICILE.......... 1<br>QUELQUE PEU DIFFICILE.......... 2<br>FACILE.......... 3	
Q643	Avez-vous l'intention de parler à votre épouse de la contraception au cours des trois prochains mois ?	OUI .... 1<br>NON .... 2<br>NE SAIT PAS.......... 8	
Q644	Pensez-vous que les autorités gouvernementales / élus locaux devraient parler publiquement de PF/ Contraception ?	OUI.......... 1<br>NON.......... 2<br>NE SAIT PAS.......... 8	
Q645	Au cours des derniers mois, avez-vous entendu ou vu un responsable gouvernemental / élus locaux parler publiquement <u>contre</u> la planification familiale ?	OUI .... 1<br>NON .... 2<br>NE SE RAPPELLE PAS.......... 8	
Q646	Au cours des derniers mois, avez-vous entendu ou vu un responsable gouvernemental / élus locaux parler publiquement <u>en faveur</u> de la planification familiale?	OUI .... 1<br>NON .... 2<br>NE SE RAPPELLE PAS.......... 8	
Q647	Pensez-vous que les responsables communautaires ou religieux devraient parler publiquement de PF/ Contraception ?	OUI.......... 1<br>NON.......... 2<br>NE SAIT PAS.......... 8	
Q648	Au cours des 12 dernièrs mois, avez-vous entendu ou vu un responsable communautaire ou religieux parler publiquement <u>contre</u> la planification familiale ?	OUI .... 1<br>NON .... 2<br>NE SE RAPPELLE PAS.......... 8	

Q649	Au cours des 12 dernièrs mois, avez-vous entendu ou vu un responsable communautaire ou religieux parler publiquement <u>en faveur</u> de la planification familiale?	OUI .... 1<br>NON .... 2<br>NE SE RAPPELLE PAS........................ 8	
Q650	Combien parmi vos proches amis/ les membres de votre famille diriez-vous qu'ils utilisent la PF/ Espacement des naissances: aucun, un peu, la plupart ou tous ?	AUCUN.. 1<br>PEU D'ENTRE-EUX............................ 2<br>LA PLUPART.................................. 3<br>TOUS.. 4<br>NE SAIT PAS................................. 8	
Q651	Pensez-vous qu'il existe des membres de votre communauté qui vous traiteront de mauvaise personne ou fuiront votre compagnie s'ils savaient que vous utilisez une méthode de PF/Espacement des naissances ?	OUI .... 1<br>NON .... 2<br>NE SAIT PAS................................. 8	
Q652	Pensez-vous qu'il existe des membres de votre communauté qui vont te féliciter, t'encourager ou dire du bien de toi s'ils savaient que vous utilisez une méthode de PF/Espacement des naissances ?	OUI .... 1<br>NON .... 2<br>NE SAIT PAS................................. 8	

	S'il vous plait, pouvez vous me dire si vous êtes totalement d'accord, d'accord, pas d'accord ou totalement contre les arguments suivants:	TOTALEMENT D'ACCORD	D'ACCORD	PAS D'ACCORD	TOTALEMENT CONTRE
Q653	Vous pouvez démarrer une conversation sur la PF avec votre partenaire/époux.	4	3	2	1
Q654	Vous pouvez convaincre votre partenaire que vous devez utiliser une méthode de PF.	4	3	2	1
Q655	Vous pouvez aller à un endroit où la PF est vendue/ offerte pour obtenir une méthode si vous décidez d'en avoir une.	4	3	2	1
Q656	Vous pouvez obtenir une méthode de PF si vous décidez d'en avoir une.	4	3	2	1
Q657	Vous pouvez utiliser une méthode de PF même si votre partenaire ne le veut pas.	4	3	2	1
Q658	Vous pouvez utiliser une méthode de PF même si aucun de vos amis ou voisins n'en utilise.	4	3	2	1
Q659	Vous pouvez utiliser une méthode de PF même si votre leader religieux pense que vous ne devrez pas l'utiliser.	4	3	2	1

Q660	S'il vous plait, en dehors de votre conjointe, donnez-moi les noms de 3 <u>principales</u> personnes avec qui vous vous sentez à l'aise pour discuter de problèmes personnels.<br><br>SI AUCUNE PERSONNE N'EST MENTIONNEE, ENREGISTRER 'AUCUNE PERSONNE'.	NOM 1 ____________________<br>NOM 2 ____________________<br>NOM 3 ____________________<br><br>AUCUNE PERSONNE: ☐	→ Q667

	Q661	Q662	Q663	Q664	Q665	Q666
**NOTEZ LE PRENOM DES 3 PERSONNES CITEES**	Quelle relation entretenez-vous avec (NOM)?	Cette personne habite-t-elle dans cette ville, une autre ville ou en zone rurale (village) ?	Avez-vous discuté de planification familiale avec(NOM) au cours des 12 derniers mois?	(NOM) utilise t-elle actuellement une méthode de PF ?	Quelle méthode (NOM) utilise t-elle ?	Cette personne pense-t-elle que vous devrez utiliser une méthode de PF ou non ?
______<br>______<br>______	PERE…………………………01<br>BEAU-PERE……………..…. 02<br>FRERE (BEAU-FRERE)…... 03<br>FILS (BEAU-FILS)…………. 04<br>ONCLE………………………. 05<br>UN AUTRE PARENT…..…… 06<br>AMI…………………………… 07<br>AUTRE ______________ 96<br>(A PRECISER)	CETTE VILLE……………… 1<br>AUTRE VILLE………………. 2<br>ZONE RURALE……………. 3	OUI…………………….… 1<br>NON……………………… 2<br>NE SAIT PAS…….…… 8	OUI……………… 1<br>NON……………… 2 → Q666<br>NE SAIT PAS….. 8	STERILISATION……….. 01<br>PILULES……………….… 02<br>DIU……………………… 03<br>INJECTABLES…………… 04<br>PRESERVATIFS……..… 05<br>AUTRE____________ 96<br>(PRECISER)<br>NE SAIT PAS………..… 98	OUI, LE PENSE…………. 1<br>NE LE PENSE PAS……… 2<br>NE SAIT PAS……………. 8
______<br>______<br>______	PERE…………………………01<br>BEAU-PERE……………..…. 02<br>FRERE (BEAU-FRERE)…... 03<br>FILS (BEAU-FILS)…………. 04<br>ONCLE………………………. 05<br>UN AUTRE PARENT…..…… 06<br>AMI…………………………… 07<br>AUTRE ______________ 96<br>(A PRECISER)	CETTE VILLE……………… 1<br>AUTRE VILLE………………. 2<br>ZONE RURALE……………. 3	OUI…………………….… 1<br>NON……………………… 2<br>NE SAIT PAS…….…… 8	OUI……………… 1<br>NON……………… 2 → Q666<br>NE SAIT PAS….. 8	STERILISATION……….. 01<br>PILULES……………….… 02<br>DIU……………………… 03<br>INJECTABLES…………… 04<br>PRESERVATIFS……..… 05<br>AUTRE____________ 96<br>(PRECISER)<br>NE SAIT PAS………..… 98	OUI, LE PENSE…………. 1<br>NE LE PENSE PAS……… 2<br>NE SAIT PAS……………. 8
______<br>______<br>______	PERE…………………………01<br>BEAU-PERE……………..…. 02<br>FRERE (BEAU-FRERE)…... 03<br>FILS (BEAU-FILS)…………. 04<br>ONCLE………………………. 05<br>UN AUTRE PARENT…..…… 06<br>AMI…………………………… 07<br>AUTRE ______________ 96<br>(A PRECISER)	CETTE VILLE……………… 1<br>AUTRE VILLE………………. 2<br>ZONE RURALE……………. 3	OUI…………………….… 1<br>NON……………………… 2<br>NE SAIT PAS…….…… 8	OUI……………… 1<br>NON……………… 2 → Q666<br>NE SAIT PAS….. 8	STERILISATION……….. 01<br>PILULES……………….… 02<br>DIU……………………… 03<br>INJECTABLES…………… 04<br>PRESERVATIFS……..… 05<br>AUTRE____________ 96<br>(PRECISER)<br>NE SAIT PAS………..… 98	OUI, LE PENSE…………. 1<br>NE LE PENSE PAS……… 2<br>NE SAIT PAS……………. 8

Q667	A votre avis, est-il acceptable pour une femme d'utiliser la contraception à l'insu de son mari ou de son partenaire si…	OUI	NON	NSP
	a. Il est contre l'utilisation de la contraception, mais elle insiste pour en utiliser?	1	2	8
	b. Ils ont beaucoup d'enfants?	1	2	8
	c. Le mari est violent envers sa femme ou ses enfants?	1	2	8
	d. Le couple ne possède pas assez d'argent pour pouvoir s'occuper d'autres enfan	1	2	8

SECTIONS 7: MESURES D'INEGALITE BASEE SUR LE SEXE

Maintenant, je voudrais vous poser des questions concernant la manière dont, à votre avis, les décisions concernant le ménage devraient être prises. S'il vous plaît, rappelez-vous que vous devez être le plus honnête possible et que vos réponses resteront confidentielles ; c'est-à-dire, que personne ne verra vos réponses.

Q701	**VERIFIEZ Q415 & Q416**<br>MARIE OU VIT ACTUELLEMENT AVEC UNE FEMME (Q416=1 OU 2) ☐ ↓<br>PAS EN UNION (Q415=3 OU Q416=3, 4 OU 5) ☐ →		**Q706**
Q702	**VERIFIEZ Q123:**<br>SI L'ENQUÊTEE GAGNE DE L'ARGENT (Q123=1 OU 2) ☐ ↓<br>SI L'ENQUÊTEE NE GAGNE PAS DE L'ARGENT (Q123 = 3 OU 4) OU NE TRAVAILLE PAS DU TOUT (Q119-2) ☐ →		**Q704**
Q703	Qui décide de comment utiliser l'argent quevous gagnez : principalement vous, principalement votre partenaire, ou vous et votre partenaire conjointement ?	REPONDANT……………………… 1<br>PARTENAIRE (S)……………………… 2<br>REPONDANT ET PARTENAIRE(S) CONJOINTEMENT……………………… 3<br>AUTRE__________________ 6<br>(A PRECISER)	
Q704	Votre /l'une de vos femmes/partenaires travaillent-elle actuellement ou gagne-t-elle de l'argent ?	OUI……………………… 1<br>NON……………………… 2	→ **Q706**
Q705	Qui décide de comment utiliser l'argent que votre/vos femme(s)/partenaires gagne(nt) : principalement vous, principalement votre/vos femme(s), ou vous et votre/ vos femmes conjointement ?	REPONDANT……………………… 1<br>PARTENAIRE (S)……………………… 2<br>REPONDANT ET PARTENAIRE(S) CONJOINTEMENT……………………… 3<br>AUTRE__________________ 6<br>(A PRECISER)	

			OUI	NON
Q706	Parfois dans le mariage ou relation amoureuse, l'homme interdit à la femme de faire certaines choses.<br><br>Interdiseriez-vous à votre femme/partenaire de:	a. Travailler en dehors de la maison ?	1	2
		b. Avoir des visites ?	1	2
		c. Rendre visite à ses amis ?	1	2
		d. Rendre visite à sa famille ?	1	2
		e. Utiliser une methode de contraception?	1	2
		f. Utiliser un téléphone mobile ?	1	2

		MARI	FEMME	ENSEMBLE	NSP/ÇA DEPEND
Q707	Selon vous, dans un couple qui devrait avoir le dernier mot dansles décisions suivantes :				
	a. Effectuer les grandes courses de la maison ?	1	2	3	8
	b. Effectuer les petites courses quotidiennes du ménage ?	1	2	3	8
	c. Décider de quand rendre visite à la famille, aux amis, ou aux parents ?	1	2	3	8
	d. Décider de quand et où chercher des soins médicaux pour votre/ vos femme(s)/ partenaire(s) ?	1	2	3	8

		OUI	NON	NSP
Q708	Souvent, un partenaire est contrarié ou vexé par certaines choses que fait sa femme. A votre avis, est-il justifié qu'un partenaire batte ou frappe sa femme dans les situations suivantes ?			
	a Si elle sort sans le lui dire?	1	2	8
	b.Si elle néglige la maison ou les enfants?	1	2	8
	c.Si elle dispute avec lui?	1	2	8
	d.Si elle refuse d'avoir des rapports sexuels avec lui?	1	2	8
	e. Si elle ne prépare pas bien la nourriture?	1	2	8
	f. Si il la suspecte d'être infidèle?	1	2	8
	g.Si elle refuse d'avoir un autre enfant?	1	2	8

Maintenant, je vais vous lire des déclarations concernant les normes basées sur le sexe et les motivations de fécondité. Pour chaque déclaration, SVP, dites-moi si vous approuvez fortement, approuvez quelque peu, désapprouvez quelque peu ou désapprouvez fortement avec ce qui est dit.					
		APPROUVE FORTEMENT	APPROUVE QUELQUE PEU	DESAPPROUVE QUELQUE PEU	DESAPPROUVE FORTEMENT
Q709	Le mari devrait être celui qui décide si le couple devrait utiliser une méthode d'espacement des naissances/de planification familiale.	4	3	2	1
Q710	Les couples qui pratiquent l'espacement des naissances/la planification familiale ont une meilleure qualité de vie que ceux qui n'en pratiquent pas.	4	3	2	1
Q711	Les maris et les femmes devraient discuter de la contraception/l'espacement des naissances.	4	3	2	1
Q712	Les hommes ne devraient pas permettre à leurs femmes d'utiliser la contraception.	4	3	2	1
Q713	Une femme qui utilise la contraception à l'insu de son mari devrait être punie.	4	3	2	1
Q714	Une femme qui n'a pas d'enfants n'est pas une femme complète.	4	3	2	1
Q715	Un homme qui n'a pas d'enfants n'est pas un homme complet.	4	3	2	1
Q716	C'est bien d'avoir beaucoup d'enfants parce que personne ne sait lequel d'entre eux survivra ou sera riche pour s'occuper de ses parents lorsque ceux-ci seront vieux.	4	3	2	1
Q717	C'est à Dieu seul de décider du nombre d'enfants qu'un couple aura.	4	3	2	1
Q718	Une femme devrait continuer de faire des enfants jusqu'à ce qu'elle ait au moins un garçon.	4	3	2	1
Q719	Une femme devrait continuer de faire des enfants jusqu'à ce qu'elle ait au moins une fille.	4	3	2	1
SQ2	L'espacement des naissances aide les parents à mieux prendre soin de leurs enfants.	4	3	2	1
SQ3	L'utilisation de l'espacement des naissances est permise seulement dans le souci de la santé de la mère et de l'enfant.	4	3	2	1
SQ4	Les maris et les femmes devraient discuter du nombre d'enfants qu'ils veulent avoir.	4	3	2	1

SECTION 8: MOUVEMENTS MIGRATOIRES			
	Maintenant, je voudrais vous demander depuis combien de temps vous vivez ici et d'où vous venez et à quelle fréquence vous visitez d'autres régions.		
Q801	Au cours des 12 derniers mois, vous est-il arrivé d'aller dans une autre ville du Sénégal pour visiter vos parents ou amis?	OUI …… 1<br>NON …… 2	 → Q811
Q802	A quelle ville êtes-vous allé le plus souvent pour rendre visite à vos parents ou amis au cours des 12 derniers mois?	NOM VILLE ____________<br>NOM DEPARTEMENT ____________<br>*à codifier au bureau* [ ][ ]<br>NOM REGION ____________<br>*à codifier au bureau* [ ][ ]	
Q803	Au cours des 12 derniers mois, à quelle fréquence avez-vous visité [LIEU MENTIONNE A Q802]?	PAR SEMAINE…… 1<br>PAR.MOIS…… 2<br>PAR.AN…… 3	
Q804	Combien de temps restez-vous habituellement lorsque vous allez là-bas ?	MINUTES…… 1<br>HEURES…… 2<br>JOURS …… 3<br>SEMAINES…… 4<br>MOIS…… 5	
Q805	Au cours de 12 derniers mois, combien de temps avez-vous passé à [NOM ENDROIT] ?	HEURES…… 1<br>JOURS …… 2<br>SEMAINES…… 3<br>MOIS…… 4	
Q806	Au cours de vos visites, vous arrive-t-il de discuter de Contraception ou de l'espacement des naissances avec quelqu'un là-bas?	OUI …… 1<br>NON …… 2	
Q807	Au cours de vos visites, vous arrive-t-il de recourir à des services de PF ou d'espacement des naissances là-bas?	OUI …… 1<br>NON …… 2	
Q808	Vos amis ou votre famille de cette ville, viennent-ils vous rendre visite?	OUI …… 1<br>NON …… 2	 → Q811
Q809	Vous arrive-t-il de parler de PF/Contraception lorsqu'ils vous rendent visite?	OUI …… 1<br>NON …… 2	
Q810	Au cours de leurs visites ici, arrive-t-il que vos visteurs recourent à des services de PF ou d'espacement des naissances?	OUI …… 1<br>NON …… 2<br>NE SAIT PAS …… 8	
Q811	Au cours des 12 derniers mois, vous est-il arrivé d'aller dans une zone rurale du Sénégal pour visiter vos parents ou amis?	OUI …… 1<br>NON …… 2	 → **FIN INTER VIEW**

Q812	A quelle zone eurale êtes-vous allé le plus souvent pour rendre visite à vos parents ou amis au cours des 12 derniersmois?	NOM VILLAGE________________________<br>NOM DEPARTEMENT _________________<br>*à codifier au bureau* [ ][ ]<br>NOM REGION ____________________<br>*à codifier au bureau* [ ][ ]	
Q813	Au cours des 12 derniers mois, à quelle fréquence avez-vous visité [LIEU MENTIONNE A Q812]?	PAR SEMAINE…………………… 1<br>PAR.MOIS………………………… 2<br>PAR.AN…………………………… 3	
Q814	Combien de temps restez-vous habituellement lorsque vous allez là-bas ?	MINUTES……………..…………. 1<br>HEURES……………………….... 2<br>JOURS …………………………… 3<br>SEMAINES………..……………. 4<br>MOIS…………………………….. 5	
Q815	Au cours de12 derniers mois, combien de temps avez-vous passé à [NOM ENDROIT] ?	HEURES……………………….... 1<br>JOURS …………………………… 2<br>SEMAINES………...…………. 3<br>MOIS…………………………….. 4	
Q816	Au cours de vos visites, vous arrive-t-il de discuter de Contraception ou de l'espacement des naissances avec quelqu'un là-bas?	OUI ………………………………………… 1<br>NON ……………………………………… 2	
Q817	Au cours de vos visites, vous arrive-t-il de recourir à des services de PF ou d'espacement des naissances là-bas?	OUI ………………………………………… 1<br>NON ……………………………………… 2	
Q818	Vos amis ou votre famille de cette zone rurale, viennent-ils vous rendre visite?	OUI ………………………………………… 1<br>NON ……………………………………… 2	**FIN INTER VIEW**
Q819	Vous arrive-t-il de parler de PF/Contraception lorsqu'ils vous rendent visite?	OUI ………………………………………… 1<br>NON ……………………………………… 2	
Q820	Au cours de leurs visites ici, arrive-t-il que vos visteurs recourent à des services de PF ou d'espacement des naissances?	OUI ………………………………………… 1<br>NON ……………………………………… 2<br>NE SAIT PAS …………………………… 8	
	HEURE DE FIN DE L'INTERVIEW	HEURE [ ][ ]<br>MINUTES [ ][ ]	

OBSERVATIONS DE L'ENQUÊTEUR
À REMPLIR APRÈS AVOIR TERMINÉ L'INTERVIEW

COMMENTAIRES SUR L'ENQUÊTÉ :

COMMENTAIRES SUR DES QUESTIONS PARTICULIÈRES :

OBSERVATIONS DU CHEF D'ÉQUIPE

NOM DU CHEF D'ÉQUIPE : ____________________ DATE : ____________

OBSERVATIONS DU SUPERVISEUR

NOM DU SUPERVISEUR ________________________________ DATE : __________
