# Supplementary material for: Association of men's exposure to family planning programming and reported discussion with partner and family planning use: The case of urban Senegal
Source: PLoS One. 2018 Sep 25;13(9):e0204049. doi: 10.1371/journal.pone.0204049 (PMC6155530; doi:10.1371/journal.pone.0204049)
Supplement: S1 Endline Men’s Questionnaire — (PDF) [file pone.0204049.s002.pdf]

SITE	GRAPPE	N° MEN	N° DIV MEN	N° LIGNE

MLE Measurement, Learning & Evaluation Project for the Urban Reproductive Health Initiative

ISSU Initiative Sénégalaise de Santé Urbaine Wellitaare

ENQUÊTE EN FIN D'ÉTUDE AUPRÈS DES MÉNAGES

2015

QUESTIONNAIRE HOMME

STRICTEMENT CONFIDENTIEL

REPUBLIQUE DU SENEGAL
MINISTÈRE DE LA SANTÉ ET L'ACTION SOCIALE

MLE/ISSU - ENQUÊTE EN FIN D'ÉTUDE AUPRÈS DES MÉNAGES - 2015

QUESTIONNAIRE HOMME

SITE	GRAPPE	N° MEN	N°LIGNE

IDENTIFICATION

SITE ____<br>(DAKAR=1, GUEDIAWAYE=2, PIKINE=3, MBAO=4)	SITE..........................
DISTRICT SANITAIRE..	DS......................
NOM DU QUARTIER ____	
NUMÉRO DE GRAPPE..	GRAPPE......
NUMÉRO DE CONCESSION..	NO CONS.....
NUMÉRO DE MÉNAGE..	N^O^ MÉN........
NOM ET NUMÉRO DE LIGNE DE L'HOMME ____	N^O^ LIGNE............

VISITES POUR L'INTERVIEW

	1	2	3	VISITE FINALE
DATE	____	____	____	JOUR ....
RÉSULTAT*	☐	☐	☐	MOIS ....
				ANNÉE 2 0 1 5
NOM DE L'ENQUÊTEUR	____	____	____	CODE RÉSULTAT.
CODE	____	____	____	CODE ENQ...
PROCHAINE VISITE DATE	____	____		NBRE TOTAL DE VISITES................
HEURE	____	____		

*CODES RÉSULTAT :

1	REMPLI	4	REFUSÉ		
2	PAS À LA MAISON	5	REMPLI PARTIELLEMENT	7	AUTRE ____ (PRÉCISER)
3	DIFFÉRÉ	6	INCAPACITÉ		

LANGUE DE QUESTIONNAIRE** ____ 1

LANGUE DE L'INTERVIEW** ____

**CODES LANGUE :

01 FRANÇAIS	04 SERER	96 AUTRES ____ (PRECISER)
02 WOLOF	05 DIOLA	
03 POULAR	06 MANDINGUE	

RECOURS A UN INTERPRÈTE : (OUI=1, NON=2)

SUPERVISEUR	CHEF D'ÉQUIPE	CONTRÔLE BUREAU	SAISI PAR :
NOM ____	NOM ____	NOM ____	NOM ____
CODE..........	CODE...........	CODE......	CODE.
DATE ____	DATE ____	DATE ____	DATE ____

SITE GRAPPE N° MEN N°LIGNE

Lettre d'information homme

Nom et prénoms de l'homme : ____

Monsieur,
Mon nom est______________________ et je travaille comme enquêteur/chef d'équipe dans une étude commanditée par Intrahealth pour le compte du Ministère de la Santé et de l'Action Sociale et réalisée par l'Agence pour la Promotion des Activités de Population - Sénégal (APAPS).

Je vous propose de participer à une enquête sur la santé maternelle et infantile qui vise à améliorer la qualité de vie des populations urbaines des villes sénégalaises à travers l'augmentation de l'accès et l'utilisation des services de santé de la reproduction. Si vous acceptez de participer, vous ferez partie des 14 000 personnes qui seront interrogées dans le cadre de cette étude. Les informations que vous allez nous fournir seront très utiles au gouvernement pour planifier les politiques de santé et répondre aux besoins non satisfaits en santé de la reproduction.

Je suis chargé de vous demander des informations sur votre accès et votre utilisation des services de santé, sur vos connaissances en matière de santé maternelle et infantile, sur votre couple ainsi que sur vos opinions et attitudes en matière de santé de la reproduction.

Dans le cadre du suivi des résultats de l'étude, il est prévu de s'entretenir avec des hommes vivant au sein des ménages échantillons tirés de l'enquête dénombrement dont votre chef de concession a eu à recevoir les enquêteurs, il y a environ un mois. Les informations obtenues seront conservée dans un endroit sécurisé.

L'entretien prend généralement entre 30 et 60 minutes. L'entretien se tiendra dans un endroit discret qui garantit la confidentialité des informations que vous nous fournirez. Si ces conditions ne sont pas réunies, nous allons arrêter l'interview jusqu'à ce que celles-ci le soient. L'exploitation de l'information sera et restera anonyme.

Vous ne tirerez personnellement aucun avantage direct de cette enquête, mais les informations que vous allez nous fournir aideront le Gouvernement à planifier ces actions futures dans le domaine de la santé de la reproduction.

Votre participation à cette enquête ne vous causera aucune gêne, hormis le fait de rester chez vous et de répondre aux questions posées. Aucun prélèvement biologique ou traitement médical n'est envisagé dans le cadre de cette enquête. S'il y a une question à laquelle vous ne voulez pas répondre, faites-le moi savoir et je passerai à la question suivante. Vous pouvez également renoncer à l'entretien à tout moment. J'espére cependant que vous participerez à cette enquête car votre opinion est particulièrement importante pour l'élaboration de programmes de santé reproductive adaptés aux besoins des populations.

Si vous acceptez de participer, vous devez signer la fiche de consentement ci jointe pour signifier que vous avez reçu l'information et marquer votre accord. Une copie de la fiche signée vous sera remise.

Vous pouvez contacter le Directeur Exécutif de l'APAPS, le Dr Ibrahima Lamine DIOP. Tél. 33 860 03 21 ou 77706 54 00// 77 553 21 42, à tout moment si vous avez des questions au sujet de l'enquête. Vous pouvez aussi contacter Dr. Samba Cor Sarr, Direction de la Santé, MSAS, 1 Rue Aimé Césaire, 2ème étage, Dakar Fann. BP 4024, Tél : 33 869 43 13, à tout moment si vous avez des questions au sujet de vos droits en tant que participant à l'enquête.

Votre participation à cette enquête est volontaire, ni votre entourage ni vous-même, ne subirez aucun préjudice si vous décidez d'arrêter votre participation.

Fait à______________, le ______________

Signature de l'enquêteur/trice

SITE GRAPPE N° MEN N°LIGNE

Fiche de consentement libre et éclairé du répondant

Je soussigné M.___________________________ atteste avoir reçu la lettre d'information contenant les explications détaillées sur le déroulement de l'enquête et les conditions de ma participation et avoir reçu une réponse satisfaisante à toutes les questions que j'ai posées.

Je retiens que ma participation est volontaire, que je ne suis pas obligé de répondre aux questions, que je pourrai mettre un terme à ma participation à tout moment si je le désire et que mon refus ne me causera aucun préjudice.

Si j'ai des questions à propos de l'enquête, je peux contacter à tout moment le Directeur Exécutif de l'APAPS, le Dr Ibrahima Lamine DIOP, Tél. 33 860 03 21//77 706 54 00//77 553 21 42. En ce qui concerne mes droits en tant que participant à l'enquête, je peux à tout moment contacter Dr. Samba Cor Sarr, Direction de la Santé, MSAS, 1 Rue Aimé Césaire, 2ème étage, Dakar Fann. BP 4024, Tél : 33 869 43 13.

OUI, ACCEPTE ET SIGNE ☐<br><br>Signature de l'enquêtée	OUI, ACCEPTE MAIS NE SOUHAITE PAS SIGNER<br><br>☐<br><br>Motifs :	NON, N'ACCEPTE PAS ☐

Fait à_______________, le _______________

SECTION 1: CARACTÉRISTIQUES DE BASE			
	**Questions et filtres**	**Modalités de codage**	**Aller à**
	**ENREGISTREZ L'HEURE**	**HEURE………** [ ][ ]<br>**MINUTES………** [ ][ ]	
Merci d'avoir accepté de participer à l'enquête. Comme je l'ai mentionné lorsque je vous ai demandé votre consentement, nous cherchons à évaluer vos besoins en santé et en informations sanitaires. Pour commencer, je vais vous poser des questions de base sur vous-mêmes.			
Q101	En quel mois et en quelle année êtes-vous né?	MOIS…………………………………… [ ][ ]<br>NE CONNAIT PAS LE MOIS………… 98<br>ANNÉE……………… [ ][ ][ ][ ]<br>NE CONNAIT PAS L'ANNÉE……… 9998	
Q102	Quel âge aviez-vous à votre dernier anniversaire ?<br>**COMPAREZ ET CORRIGEZ 101 ET/OU 102 SI INCOHÉRENT**	AGE EN ANNÉES RÉVOLUES [ ][ ]	
Q103	VÉRIFIEZ 101 & 102<br>A-T-IL ENTRE 15 ET 59 ANS ?<br>SI OUI, CONTINUER L'INTERVIEW : [ ]	SI NON : [ ]	**ARRET ER L'INTER**
Q104	Au cours des 12 derniers mois, à combien d'occasions différentes avez-vous voyagé hors de votre lieu résidence régulière et dormi ailleurs pendant au moins une nuit ?	NOMBRE DE DÉPLACEMENTS... [ ][ ]<br>AUCUN ………………………………………… 00<br>NE SE SOUVIENT PAS…………………… 98	 **Q106**
Q105	Au cours des 12 derniers mois, avez vous été absent de votre domicile pendant plus d'un mois d'affilé ?	OUI…………………………………………… 1<br>NON…………………………………………… 2	
Q106	Avez-vous fréquenté l'école?	OUI…………………………………………… 1<br>NON…………………………………………… 2	 **Q109**
Q107	Quel est le plus haut niveau d'études que vous avez atteint: Coranique seulement, Primaire, secondaire ou supérieur?	CORANIQUE SEULEMENT……………….. 0<br>PRIMAIRE……………………………………… 1<br>SECONDAIRE 1………………………………… 2<br>SECONDAIRE 2………………………………… 3<br>SUPÉRIEUR……………………………………… 4	**Q110**<br><br><br><br>**Q111**
Q108	Quelle est (l'année/classe) la plus élevée que vous avez achevée à ce niveau ?<br>**SI AUCUNE ANNÉE/CLASSE ACHEVÉE A CE NIVEAU, REPORTEZ "00"**	CLASSE / ANNÉE ……………………… [ ][ ]	
Q109	**VERIFIEZ 107:**<br>**PRIMAIRE**<br>**(Q107=1) :** [ ]	**SECONDAIRE 1 OU SECONDAIRE 2**<br>**(Q107=2 OU 3) :** [ ]	**Q111**
Q110	Maintenant, je voudrais vous demander de me lire cette phrase.<br>**MONTREZ A L'ENQUETE UNE PHRASE FIGURANT SUR LA CARTE D'ALPHABETISATION**<br>**SI L'ENQUETE NE PEUT PAS LIRE LA PHRASE ENTIERE, INSISTEZ** : Pouvez-vous me lire certaines parties de la phrase?	NE PEUT PAS LIRE DU TOUT ………………… 1<br>PEUT LIRE SEULEMENT CERTAINES PARTIES DE LA PHRASE ……………. 2<br>PEUT LIRE TOUTE LA PHRASE ………….. 3<br>PAS DE CARTE DANS LA LANGUE QUI CONVIENT ________________ 4<br>(PRECISER LA LANGUE)<br>AVEUGLE/MALVOYANT……………………… 5	

Q111	Quelle est votre religion?	CATHOLIQUE………………………………… 1<br>AUTRE RELIGION CHRÉTIENNE………………… 2<br>MUSULMAN………………………………………… 3<br>TRADITIONNELLE………………………………… 4<br>AUCUNE RELIGION……………………………… 5<br>AUTRE____________________________ 6<br>(PRÉCISER)	 <br><br><br>→ **Q113**
Q112	Dans quelle mesure vos croyances religieuses influencent-elles vos décisions en matière de PF ?<br>Diriez vous: Pas du tout, quelques fois, souvent ou toujours?	PAS DU TOUT………………………………… 1<br>PARFOIS… …………………………………… 2<br>SOUVENT……………………………………… 3<br>TOUJOURS……………………………………… 4<br>NE CONNAIT PAS LA PF…………………… 8	
Q113	Quel est votre groupe ethnique?	WOLOF/LEBOU………………………………… 01<br>POULAR……………………………………………… 02<br>SERER………………………………………………… 03<br>DIOLA………………………………………………… 04<br>MANDINGUE………………………………………… 05<br>SONINKÉ……………………………………………… 06<br>AUTRE ETHNIE ÉTRANGÈRE……………… 07<br>AUTRE____________________________ 96<br>(PRÉCISER)	
Q114	Quelle langue parlez-vous habituellement dans votre maison?	WOLOF/LEBOU………………………………… 01<br>POULAR……………………………………………… 02<br>SERER………………………………………………… 03<br>DIOLA………………………………………………… 04<br>MANDINGUE………………………………………… 05<br>SONINKÉ……………………………………………… 06<br>AUTRE ETHNIE ÉTRANGÈRE……………… 07<br>AUTRE____________________________ 96<br>(PRÉCISER)	
Q115	Au cours des 12 derniers mois, avez-vous eu un travail, quel qu'il soit ?	OUI……………………………………………………… 1<br>NON…………………………………………………… 2	→ **Q117**
Q116	Qu'avez-vous fait la plupart du temps au cours des 12 derniers mois?	RIEN…………………………………………………… 01<br>A LA RECHERCHE D'UN EMPLOI……………… 02<br>ALLER A L'ÉCOLE/ ÉTUDIER ………………… 03<br>INCAPABLE DE TRAVAILLER/<br>HANDICAPE……………………………………… 04<br>RETRAITE ………………………………………… 05<br>TRAVAUX DOMESTIQUES/S'OCCUPER<br>DES ENFANTS …………………………………… 06<br>AUTRE______________________________ 96<br>(A PRÉCISER)	} → **Q201**
Q117	Êtes-vous employé par un membre de votre famille, par quelqu'un d'autre, ou travaillez vous à votre compte?	POUR UN MEMBRE DE LA FAMILLE………… 1<br>POUR QUELQU'UN D'AUTRE……………………… 2<br>EST A SON COMPTE PERSONNEL……………… 3	
Q118	Travaillez-vous d'habitude pendant toute l'année, de manière saisonnière, ou seulement de temps en temps?	PENDANT TOUTE L'ANNÉE …………………… 1<br>DE MANIÈRE SAISONNIÈRE/UNE<br>PARTIE DE L'ANNÉE ………………………… 2<br>DE TEMPS EN TEMPS ………………………… 3	
Q119	Que faites-vous comme travail, c'est-à-dire, quel genre de travail faites-vous principalement ?	PROFESSIONNEL/TECHNICIEN/<br>ADMINISTRATIF…………………………………… 1<br>VENTES ET SERVICES………………………… 2<br>TRAVAIL MANUEL NON AGRICOLE………… 3<br>AGRICULTURE…………………………………… 4<br>AUTRE ______________________________ 6<br>(A PRÉCISER)	
Q120	Etes-vous payé en espèces ou en nature pour ce travail, ou n'êtes-vous pas payé du tout?	SEULEMENT EN ESPECES ………………… 1<br>EN ESPECES ET EN NATURE …………… 2<br>SEULEMENT EN NATURE ………………… 3<br>PAS PAYE ………………………………………… 4	

	SECTION 2: REPRODUCTION		
	**Questions et filtres**	**Modalités de codage**	**ALLER A**
Maintenant, je voudrais vous poser des questions sur vos enfants. S'il vous plaît, soyez le plus honnête possible et sachez que vos réponses ne seront communiquées à personne d'autre.			
Q201	Maintenant, je voudrais vous interroger sur les enfants que vous avez eus pendant votre vie. Je suis uniquement intéressé par les enfants qui sont biologiquement les vôtres.<br>Avez-vous déjà été/Êtes-vous pére d'un ou de plusieurs enfants?	OUI…………………………..…………………. 1<br>NON…………………………………….……… 2<br>NE SAIT PAS……………………….……… 8	 **Q206**<br>**Q206**
Q202	Y a-t-il des fils ou des filles dont vous êtes le père et qui vivent actuellement avec vous ?	OUI…………………………..…………………. 1<br>NON…………………………………….……… 2	 **Q204**
Q203	Combien de fils vivent avec vous ?<br>Et combien de filles vivent avec vous?	FILS VIVANT A LA MAISON…..[ ][ ]<br>FILLES VIVANT A LA MAISON..[ ][ ]	
Q204	Y a-t-il des fils ou des filles dont vous êtes père, qui sont toujours en vie, mais qui ne vivent pas avec vous ?	OUI…………………………..…………………. 1<br>NON…………………………………….……… 2	 **Q206**
Q205	Combien de fils sont en vie mais ne vivent pas avec vous?<br>Et combien de filles sont en vie mais ne vivent pas avec vous ?	FILS VIVANT AILLEURS ..…….[ ][ ]<br>FILLES VIVANT AILLEURS …..[ ][ ]	
Q206	Avez-vous eu un fils ou une fille qui est né vivant(e) mais qui est décédé(e) par la suite ?<br>**SI NON, INSISTEZ: UN BEBE AYANT CRIE OU AYANT MONTRE DES SIGNES DE VIE, MAIS QUI N'A PAS SURVECU?**	OUI…………………………...………………1<br>NON…...…………………...…………………2<br>NE SAIT PAS………………………..……… 8	 **Q208**<br>**Q208**
Q207	Combien de garçons sont décédés?<br>Et combien de filles sont décédées?	FILS DECEDES…………….....….[ ][ ]<br>FILLES DECEDEES ……………[ ][ ]	
Q208	FAIRE LA SOMME DES REPONSES AUX QUESTIONS Q203, Q205, ET Q207, ET ECRIVEZ LE TOTAL<br>**S'IL N'Y EN A AUCUN, ECRIVEZ '00'.**	TOTAL D'ENFANTS ……………[ ][ ]	
Q209	**VERIFIEZ Q208:**<br>A UN ENFANT OU DES ENFANTS : [ ] ↓	N'A PAS EU D'ENFANTS : [ ]	**Q214**
Q210	Quel était votre âge lorsque vous êtes devenu père <u>pour la première fois</u> ?<br>(AGE EN ANNEES REVOLUES)	AGE EN ANNEES ….…………...[ ][ ]<br>NE SAIT PAS/NE SE RAPPELLE PAS….. 98	
Q211	Quel âge a votre plus jeune enfant vivant (dernier-né)?<br>**(AGE EN MOIS SI MOINS D'UN AN ; SI MOINS D'UN MOIS, METTEZ 00; AGE EN ANNEES REVOLUES SI PLUS D'UN AN)**	AGE EN MOIS…..……………. 1 [ ][ ]<br>AGE EN ANNEES …………. 2 [ ][ ]<br>PAS D'ENFANTS VIVANTS……………….. 995<br>NE SAIT PAS/NE SE RAPPELLE PAS …. 998	
Q212	Au moment où vous avez eu votre plus jeune enfant (dernier-né), vouliez-vous avoir des enfants <u>à ce moment,</u> vouliez-vous attendre <u>plus tard,</u> ou vouliez-vous <u>ne plus/ne pas avoir d'enfants</u> ?	A CE MOMENT ……………………………1<br>PLUS TARD………………………………… 2<br>NE PAS/NE PLUS AVOIR D'ENFANTS … 3	**Q214**<br><br>**Q214**

Q213	Si cela dépendait de vous, combien de temps de plus auriez-vous souhaité attendre pour avoir votre (plus jeune) enfant ?	MOIS………………………… 1 [ ][ ]<br>ANNEES…………………… 2 [ ][ ]<br>AUTRE ___________________ 996<br>NE SAIT PAS……………………… 998	
Q214	*A présent, je voudrais vous poser des questions sur les risques de survenue d'une grossesse chez la femme.*<br>Entre la période des règles et les règles suivantes, y-a-t-il un moment où une femme a plus de chances de tomber enceinte que d'autres, si elle a des rapports sexuels ?	OUI ……………………………………………1<br>NON……………………………………………2<br>NSP……………………………………………8	 <br>**Q216**<br>**Q216**
Q215	A quelle période du cycle menstruel ces jours correspondent t-ils ? Juste avant le début des règles, Pendant les règles, Juste après la fin des règles, Au milieu entre 2 périodes ?<br>**UNE SEULE REPONSE EST ENREGISTREE.**	JUSTE AVANT LES REGLES…………………… 1<br>PENDANT LES REGLES……………………… 2<br>TOUT JUSTE APRES LA FIN DES REGLES.. 3<br>AU MILIEU ENTRE 2 PERIODES……………4<br>AUTRE______________________________ 6<br>(PRECISER)<br>NE SAIT PAS……………………………… 8	
Q216	Pensez-vous qu'une femme qui allaite son bébé au sein peut tomber enceinte ?	OUI ……………………………………………… 1<br>NON……………………………………………2<br>CELA DEPEND…………………………………3<br>NSP……………………………………………8	 <br><br>**Q301**
Q217	Dans quelles conditions l'allaitement maternel peut-être considéré comme une méthode contraceptive efficace ?<br>**INSISTER: Quelque chose d'autre?**<br>**ENREGISTRER TOUT CE QUI EST MENTIONNE.**	ALLAITEMENT MATERNEL EXCLUSIF (PAS DE SUPPLEMENTS)………… A<br>ALLAITEMENT MATERNEL EXCLUSIF JUSQU' A 6 MOIS……………………B<br>AVANT LE RETOUR DE COUCHES…..... C<br>AUTRES___________________________ X<br>(PRECISER)<br>AUCUNE…………………………………… Y<br>NE SAIT PAS……………………………… Z	

SECTION 3: CONTRACEPTION ET IMPLICATION DE L'HOMME

	VERIFIEZ LA PRESENCE D'AUTRES PERSONNES. AVANT DE CONTINUER, FAITES TOUT DISCRETEMENT POUR GARANTIR UN CADRE INTIME.		
	Maintenant, je voudrais vous poser des questions sur la planification familiale, sur les divers moyens ou les diverses méthodes qu'un couple peut utiliser pour retarder ou éviter une grossesse.		
	Quels sont les moyens ou les méthodes dont vous avez entendu parler?<br><br>**POUR LES METHODES QUI N'ONT PAS ETE MENTIONNEES SPONTANEMENT, DEMANDEZ:**<br><br>ENCERCLEZ '1' POUR CHAQUE METHODE MENTIONNEE SPONTANEMENT. PUIS PROCEDEZ EN LISANT DANS LA COLONNE EN BAS LE NOM ET LA DESCRIPTION DE CHAQUE METHODE QUI N'A PAS ETE MENTIONNEE SPONTANEMENT.<br>ENCERCLEZ '2' POUR CHAQUE METHODE RECONNUE APRES ECOUTE DE LA DESCRIPTION.<br>ENCERCLEZ '3' POUR CHAQUE METHODE QUI N'A PAS ÉTÉ MENTIONNEE NI RECONNUE.		
		301. Avez-vous déjà entendu parler de (METHODE)?<br><br>OUI(Mentionnée spontanément)…1<br>OUI (Après description).?……..…...2<br>NON………..……………………..…3	302.Vous ou votre partenaire , avez-vous déjà utilisé (METHODE)?
1	LA STERILISATION FEMININE les femmes peuvent subir une opération pour éviter d'avoir des enfants.	1 2 3 ↓ METHODE SUIVANTE	Votre partenaire a-t-elle jamais effectué une opération de stérilisation pour éviter d'avoir des enfants ?<br>OUI……… 1<br>NON…….. 2
2	LA STERILISATION MASCULINE/VASECTOMIE Les hommes peuvent subir une opération pour éviter d'avoir des enfants.	1 2 3 ↓ METHODE SUIVANTE	Avez-vous jamais effectué une opération (vasectomie) pour éviter d'avoir des enfants ?<br>OUI…..…. 1<br>NON…….. 2
3	LES PILULES JOURNALIERES Les femmes peuvent avaler une pilule chaque jour pour éviter de tomber enceinte.	1 2 3 ↓ METHODE SUIVANTE	OUI……… 1<br>NON…….. 2<br>NSP……… 8
4	DIU Les femmes peuvent avoir un stérilet que le médecin, la sage-femme ou l'infirmier/ière leur place à l'intérieur de l'utérus.	1 2 3 ↓ METHODE SUIVANTE	OUI……… 1<br>NON…….. 2<br>NSP……… 8
5	INJECTABLES Les femmes peuvent se faire faire une injection par un prestataire de santé pour éviter de tomber enceinte pendant un ou plusieurs mois.	1 2 3 ↓ METHODE SUIVANTE	OUI……… 1<br>NON…….. 2<br>NSP……… 8
6	IMPLANTS Les femmes peuvent se faire placer sous la peau du bras un produit se présentant sous la forme d'un ou plusieurs petits bâtonnets qui vont les empêcher de tomber enceinte pendant un ou plusieurs années.	1 2 3 ↓ METHODE SUIVANTE	OUI……… 1<br>NON…….. 2<br>NSP……… 8
7	PRESERVATIF MASCULIN Les hommes peuvent porter un préservatif avant l'acte sexuel.	1 2 3 ↓ METHODE SUIVANTE	OUI……… 1<br>NON…….. 2
8	PRESERVATIF FEMININ Les femmes peuvent placer un préservatif avant l'acte sexuel.	1 2 3 ↓ METHODE SUIVANTE	OUI……… 1<br>NON…….. 2<br>NSP……… 8
9	LA METHODE DU RYTHME chaque mois, pendant qu'elle est sexuellement active, une femme peut éviter de tomber enceinte en n'ayant pas de rapports sexuels les jours du mois pendant lesquels elle court beaucoup de risques de tomber enceinte.	1 2 3 ↓ METHODE SUIVANTE	OUI……… 1<br>NON…….. 2

10	LA METHODE DU RETRAIT Les hommes peuvent éviter une grossesse à leur partenaire en se retirant avant d'éjaculer.	1 2 3 → METHODE SUIVANTE	OUI……… 1<br>NON…….. 2	
11	CONTRACEPTION D'URGENCE Les femmes peuvent prendre des pilules jusqu'à cinq jours après un rapport sexuel pour éviter de tomber enceinte.	1 2 3 → METHODE SUIVANTE	OUI……… 1<br>NON…….. 2<br>NSP……… 8	
12	METHODE DE L'ALLAITEMENT MATERNEL ET DE L'AMENORRHEE (MAMA) Jusqu'à 6 mois après l'accouchement, une femme peut utiliser une méthode qui exige qu'elle allaite fréquemment son bébé, le jour comme la nuit, et à condition qu'elle n'ait pas encore eu le retour de couche.	1 2 3 → METHODE SUIVANTE	OUI……… 1<br>NON…….. 2<br>NSP……… 8	
13	COLLIER DU CYCLE Les femmes peuvent utiliser un collier contraceptif pour éviter des rapports sexuels non protégés pendant la période de fécondité	1 2 3 → METHODE SUIVANTE	OUI……… 1<br>NON…….. 2<br>NSP……… 8	
14	AUTRES MÉTHODES NATURELLES<br>Les femmes peuvent utiliser d'autre methodes naturelles comme abstinance	OUI………………………… 1<br>____________________<br>(A PRECISER)<br>NON……………………… 3 → MÉTHODE SUIVANTE	OUI……… 1<br>NON…….. 2<br>NSP……… 8	
15	Avez-vous entendu parler d'autres moyens ou d'autres méthodes que les femmes ou les hommes peuvent utiliser pour éviter la grossesse ?<br>**NOTE: SI L'ENQUETE MENTIONNE L'ABSTINENCE COMME UNE METHODE DE PF, NE PAS LE REPORTER. INSISTER POUR TOUTE AUTRE METHODE CONNUE.**	OUI………………………… 1<br>____________________<br>(A PRECISER)<br>NON……………………… 3 → Q303	OUI……… 1<br>NON…….. 2<br>NSP……… 8	
Q303	**VERIFIEZ Q301**<br>**SI L'ENQUETE A ENTENDU PARLER D'AU MOINS UNE METHODE (Q301=1 OU 2)** ☐ ↓	**SI LA REPONSE EST 'NON' (3) A TOUTES** ☐		**→Q401**
Q304	Au cours de 12 derniers mois, où avez-vous vu ou entendu parler de contraception / PF ou d'espacement des naissances ?<br><br>INSISTER: D'AUTRES SOURCES?<br><br>ENREGISTRER TOUT CE QUI EST MENTIONNE	**MEDIA**<br>RADIO……………………………………… AA<br>TELEVISION…………………………………… AB<br>JOURNAL……………………………………… AC<br>MAGAZINE……………………………………… AD<br>PANNEAUX D'AFFICHAGE……………………… AE<br>THEATRE SUR SCENE………………………… AF<br>ÉVENEMENTS COMMUNAUTAIRES……….. AG<br>FLYERS/DEPLIANTS…………………… AH<br>INTERNET……………………………………… AI<br>NUMERO VERT………………………………… AJ<br>**SECTEUR PUBLIC**<br>HOPITAL GOUVERNEMENTAL……………… BA<br>CENTRE SANTE GOUVERNEMENTAL……. BB<br>POSTE DE SANTE……………………………… BC<br>STRATEGIE AVANCEE/EQUIPE MOBILE…. BD<br>CENTRE CONSEILS ADOS…………………… BE<br>CASE DE SANTE……………………………… BF<br>AUTRE PUBLIC………………………………… BG<br>**SECTEUR PRIVE FORMEL**<br>HOPITAL/CLINIQUE/CABINET PRIVE……… CA<br>PHARMACIE…………………………………… CB<br>DISPENSAIRE RELIGIEUX…………………… CC<br>AUTRE MEDICAL PRIVE……………………… CD<br>**SECTEUR PRIVE INFORMEL**<br>MEDECIN………………………………………… DA<br>SAGE-FEMME…………………………………… DB<br>INFIRMIER/AI…………………………………… DC<br>MATRONE / ASC ……………………………… DD<br>GUERISSEUR /ACCOUCHEUSE DE<br>**AUTRES SOURCES**<br>ÉCOLE…………………………………………… EA<br>ÉGLISE/MOSQUEE……………………………… EB<br>BAR……………………………………………… EC<br>PARENTS/AMIS ………………………………… ED<br>ONG/OCB/RELAIS/BAJENN GOKH………… EE<br>**AUTRE**____________________________ XX<br>(PRECISER)<br>N'A NI VU, NI ENTENDU………………………… YY<br>NSP……………………………………………… ZZ		

Q305	**VERIFIEZ Q302.**<br>A DEJA UTILISE AU MOINS UNE METHODE (AU MOINS UN "1" DANS Q302) : ☐	N'A JAMAIS UTILISE UNE METHODE (PARTOUT "2" A Q302) : ☐	**Q317**
Q306	Maintenant, je voudrais vous poser des questions sur la première fois que vous avez fait quelque chose ou utilisé une méthode pour éviter d'avoir un enfant.<br>Combien d'enfants vivants aviez-vous en ce moment?<br>Combien de fils vivants aviez-vous en ce moment?<br>Combien de filles vivants aviez-vous en ce moment?<br>**S'IL N'Y EN A AUCUN, ENREGISTREZ '00'.**	NOMBRE D'ENFANTS............ ☐☐<br>AUCUN................................ 00<br>NOMBRE DE FILS................. ☐☐<br>NOMBRE DE FILLES............. ☐☐	 **Q307**
Q307	Vous (ou votre femme/partenaire) faites-vous quelque chose ou utilisez-vous une méthode actuellement pour retarder ou éviter d'avoir une grossesse ?	OUI.. 1<br>NON... 2<br>NSP.. 8	 **Q317**<br>**Q317**
Q308	Quelles méthodes utilisez-vous actuellement vous et votre femme/partenaire dans votre couple ?<br>SI PILULE, INSISTER POUR SAVOIR SI PILULE JOURNALIERE OU CONTRACEPTION D'URGENCE.<br>INSISTER: D'autres méthodes?<br>ENCERCLER TOUT CE QUI EST MENTIONNE.	STERILISATION FEMININE .... A<br>STERILISATION MASCULINE............................. B<br>IMPLANT.. C<br>DIU .... D<br>INJECTABLES... E<br>PILULE.. F<br>CONTRACEPTION D'URGENCE......................... G<br>PRESERVATIF MASCULIN................................ H<br>PRESERVATIF FEMININ.................................... I<br>COLLIER DU CYCLE... J<br>ALLAITEMENT AU SEIN /MAMA.......................... K<br>AUTRES MÉTHODES MODERNES<br>_______________________________ L<br>(PRÉCISER)<br>MÉTHODE DU RYTHME..................................... M<br>RETRAIT.. N<br>AUTRES MÉTHODES TRADITIONNELLES<br>_______________________________ X<br>(PRÉCISER)	
Q309	**VERIFIEZ Q308 :**<br>**SI PLUSIEURS METHODES MENTIONNEES, ENCERCLEZ DANS CETTE LISTE CELLE DONT LE CODE DE LA MODALITE EST ARRIVE LE PREMIER PAR ORDRE ALPHABETIQUE ET CONTINUEZ AVEC CELLE-CI POUR LES QUESTIONS SUIVANTES.**<br>**SI UNE SEULE METHODE EST MENTIONNEE, ENCERCLEZ CETTE MÊME METHODE DANS CETTE LISTE.**	STERILISATION FEMININE .... 01<br>STERILISATION MASCULINE............................. 02<br>IMPLANT.. 03<br>DIU .... 04<br>INJECTABLES... 05<br>PILULE.. 06<br>CONTRACEPTION D'URGENCE......................... 07<br>PRESERVATIF MASCULIN................................ 08<br>PRESERVATIF FEMININ.................................... 09<br>COLLIER DU CYCLE... 10<br>ALLAITEMENT AU SEIN /MAMA.......................... 11<br>AUTRES MÉTHODES MODERNES 12<br>MÉTHODE DU RYTHME..................................... 13<br>RETRAIT.. 14<br>AUTRES MÉTHODES TRADITIONNELLES 15	
Q310	Pourquoi avez-vous choisi d'utiliser cette méthode? (METHODE ENCERCLEE EN Q309)<br>INSISTEZ : Existe-t-il une autre raison ?<br>ENREGISTRER TOUT CE QUI EST MENTIONNE.	NE VEUT PAS RENDRE ENCEINTE.................. A<br>PAS D'EFFETS SECONDAIRES......................... B<br>NE VEUT PAS ÊTRE INFECTÉ PAR LE VIH OU LES AUTRES ISTs............................ C<br>FACILE / PRATIQUE À UTILISER....................... D<br>METHODE DISCRETE.. E<br>COÛT ABORDABLE.. F<br>FACILE A OBTENIR.. G<br>BEAUCOUP DE PERSONNES L'UTILISENT...... H<br>J'AIME COMME ON LE PREND AU QUOTIDIEN I<br>J'AIME COMME ON NE LE PREND PAS AU QUOTIDIEN.................................... J<br>FAIT PERDRE DU POIDS A MA PARTENAIRE.. K<br>FAIT GAGNER DU POIDS A MA PARTENAIRE. L<br>DONNE/MAINTIENT BON TEINT A PARTENAIF M<br>RECOMMANDÉ PAR UN PRESTATAIRE........... N<br>MA PARTENAIRE LA PRÉFÈRE......................... O<br>JE N'AI PAS À M'OCCUPER DE ÇA; MA PARTENAIRE EST RESPONSABLE DU CH(P<br>AUTRE _______________________ X<br>(PRÉCISER)	

Q311	Qui décide de quelle méthode contraceptive utiliser ou ne pas utiliser ? Est-ce vous principalement, votre partenaire principalement, ou décidez-vous ensemble?	VOUS PRINCIPALEMENT…………………………… 1<br>PARTENAIRE PRINCIPALEMENT................ 2<br>CONJOINTEMENT…………………………………… 3<br>AUTRE_____________________________ 6<br>(PRECISER)	
Q312	Un prestataire de service de sante/PF vous a-t-il jamais parlé d'autres methodes contraceptives en dehors celle que vous/votre partenaire utilisez actuellement ?	OUI……………………………………………………… 1<br>NON……………………………………………………… 2<br>NSP……………………………………………………… 8	
Q313	**VERIFIEZ Q309:**<br>**SI PRESERVATIF MASCULIN "8"** ☐<br>↓<br>**SI STERILISATION FEMININE "1", STERILISATION MASCULINE "2" IMPLANTS "3", DIU "4", INJECTABLES "5", PILULES "6", CONTRACEPTION D'URGENCE "7", PRESERVATIF FEMININ "9" COLLIER DU CYCLE "10" OU AUTRE METHODES MODERNES "12"** ☐ → **Q316**<br>**SI ALLAITEMENT AU SEIN /MAMA "11", MÉTHODE DU RYTHME "13", RETRAIT "14" OU AUTRES MÉTHODES TRADITIONNELLES "15"** ☐ → **Q318**		
Q314	Où (de qui) aviez-vous obtenu la dernière fois les condoms ?<br><br>**ENCERCLEZ LE TYPE DE STRUCTURE APPROPRIE.**	**SECTEUR PUBLIC**<br>HOPITAL GOUVERNEMENTAL……………… 11<br>CENTRE SANTE GOUVERNEMENTAL……… 12<br>POSTE DE SANTE…………………………… 13<br>STRATEGIE AVANCEE/EQUIPE MOBILE….. 14<br>CENTRE CONSEILS ADOS…………………… 15<br>CASE DE SANTE…………………………… 16<br>AUTRE PUBLIC……………………………… 17<br><br>**SECTEUR PRIVE FORMEL**<br>HOPITAL/CLINIQUE/CABINET PRIVE……… 21<br>PHARMACIE………………………………… 22<br>DISPENSAIRE RELIGIEUX………………… 23<br>AUTRE MEDICAL PRIVE…………………… 24<br><br>**SECTEUR PRIVE INFORMEL**<br>MEDECIN……………………………………… 31<br>SAGE-FEMME………………………………… 32<br>INFIRMIER/AI………………………………… 33<br>MATRONE / ASC .... 34<br>GUERISSEUR /ACCOUCHEUSE TRADITIONNELLE………………………… 35<br><br>**AUTRES SOURCES**<br>CLINIQUE AU LIEU DE TRAVAIL…………… 41<br>CENTRE POUR JEUNES…………………… 42<br>DISTRIBUTEUR AUTOMATIQUE…............ 43<br>CENTRE DE CONSEIL/TEST DE VIH……. 44<br>BAR/CAFE…………………………………… 45<br>KIOSQUE/BOUTIQUE/MARCHE…………… 46<br>ONG…………………………………………… 47<br>VOLONTAIRES/PAIRS ÉDUCATEURS……. 48<br>AMIS…………………………………………… 49<br>**AUTRE**_____________________________ 96<br>(PRECISER)<br>NSP…………………………………………… 98	
Q315	Combien de préservatifs (**unités**) avez-vous payés / obtenues la dernière fois ?	NOMBRE DE PRESERVATFS………. ☐☐<br><br>NE SAIT PAS……………………………98	
Q316	Quel est le prix que vous avez payé pour cette méthode **(METHODE ENREGISTREE A Q309)** la dernière fois?	MONTANT (FCFA).. ☐☐☐☐☐<br>GRATUIT……………………………00000<br>NE SAIT PAS…………………………99998	**Q318**

Q317	Quelles sont les principales raisons pour lesquelles vous n'utilisez pas actuellement une méthode de planification familiale pour retarder ou éviter une grossesse?<br><br>INSISTER: D'autres raisons?<br><br>**ENREGISTRERTOUT CE QUI EST MENTIONNE**.	**RAISONS DE FECONDITE**<br>PAS DE RAPPORTS SEXUELS……………..…. A<br>RAPPORTS SEXUELS NON FRÉQUENTS B<br>PAS ENCORE MARIÉ / PAS DE PARTENAIRI C<br>PARTENAIRE/FEMME ABSENT...…………… D<br>PARTENAIRE ENCEINTE……...………………E<br>PARTENAIRE ALLAITE…………………………F<br>RÉCEMMENT EU UN BÉBÉ…………………… G<br>VEUT PLUS D'ENFANTS / SOUHAITE QUE SA FEMME TOMBE ENCEINTE…………………..H<br>PARTENAIRE EN MENOPAUSEE/HYSTERE I<br>LUI OU PARTNENAIRE NE PEUT PAS EN A\ J<br>**OPPOSITION A L'UTILISATION**<br>L'ENQUETE EST OPPOSE……..…….......... K<br>LA PARTENAIRE EST OPPOSEE…………..... L<br>D'AUTRES PERSONNES SONT OPPOSEES M<br>INTERDICTION RELIGIEUSE……………........ N<br>**MANQUE DE CONNAISSANCE** :<br>NE SAIT PAS QUELLE MÉTHODE UTILISER O<br>NE SAIT PAS COMMENT UTILISER UNE METHODE……………….………… P<br>NE CONNAIT AUCUNE SOURCE……...….… Q<br>**RAISONS LIEES A LA METHODE**<br>PROBLEMES DE SANTE POUR LUI OU FEM R<br>PEUR DES EFFETS SECONDAIRES………. S<br>MANQUE D'ACCES / TROP ELOIGNE………T<br>COUTE TROP CHERE…………..………… U<br>PAS PRATIQUE A UTILISER……….……... V<br>N'AIME PAS LES METHODES EXISTANT….. W<br>EXPERIENCES MALHEUREUSES AVEC LES METHODES EXISTANT…………… X<br>FATALISTE: DEPEND DE DIEU ……..…..…. Y<br>AUTRE________________________________ WW<br>(PRECISER)<br>AUTRE________________________________ XX<br>(PRECISER)<br>AUTRE________________________________ YY<br>(PRECISER)<br>NE SAIT PAS …………..…..……………………. ZZ	 <br><br><br><br><br><br><br><br><br>I → **Q322**<br>J → **Q322**
Q318	Pensez-vous que vous (ou votre femme/partenaire) utiliserez une méthode pour retarder ou éviter une grossesse au cours des 12 prochains mois ?	OUI……………………….…...…………..………. 1<br>NON……………………….……………………. 2<br>NE SAIT PAS………………………...……….…. 8	 → **Q322**<br>→ **Q322**
Q319	Quelle méthode préfèreriez-vous utiliser **<u>LE PLUS</u>**, si vous utilisez effectivement une méthode à l'avenir?<br><br>SI PILULE, INSISTER POUR SAVOIR SI PILULE JOURNALIERE OU CONTRACEPTION D'URGENCE.	STERILISATION FEMININE .... 01<br>STERILISATION MASCULINE............................ 02<br>IMPLANT.........................…………………….... 03<br>DIU ……………………………………….......... 04<br>INJECTABLES…………………………………... 05<br>PILULE...……………………………………...... 06<br>CONTRACEPTION D'URGENCE……………..… 07<br>PRESERVATIF MASCULIN................................ 08<br>PRESERVATIF FEMININ.................................... 09<br>COLLIER DU CYCLE……………………………. 10<br>ALLAITEMENT AU SEIN /MAMA………………... 11<br>AUTRES MÉTHODES MODERNES……………………… 12<br>MÉTHODE DU RYTHME…………………………… 13<br>RETRAIT……………………………………………… 14<br>AUTRES MÉTHODES TRADITIONNELLES……………. 15	 <br><br><br><br><br><br><br><br><br>11 → **Q322**<br><br>13 → **Q322**<br>14 → **Q322**
Q320	Connaissez-vous un endroit ou une personne où vous pouvez obtenir cette méthode (METHODE ENCERCLER A Q319)?	OUI…………………...……...………….………. 1<br>NON……………………….……………………. 2	 → **Q322**

Q321	Quel est le type de place/personne où vous pouvez obtenir cette méthode (METHODE ENCERCLER A Q319)?<br><br>**ENCERCLEZ LE TYPE DE STRUCTURE APPROPRIE.**	**SECTEUR PUBLIC**<br>HOPITAL GOUVERNEMENTAL............... 11<br>CENTRE SANTE GOUVERNEMENTAL..... 12<br>POSTE DE SANTE................................ 13<br>STRATEGIE AVANCEE/EQUIPE MOBILE.. 14<br>CENTRE CONSEILS ADOS..................... 15<br>CASE DE SANTE.................................. 16<br>AUTRE PUBLIC.................................... 17<br><br>**SECTEUR PRIVE FORMEL**<br>HOPITAL/CLINIQUE/CABINET PRIVE........ 21<br>PHARMACIE.. 22<br>DISPENSAIRE RELIGIEUX....................... 23<br>AUTRE MEDICAL PRIVE.......................... 24<br><br>**SECTEUR PRIVE INFORMEL**<br>MEDECIN.. 31<br>SAGE-FEMME.. 32<br>INFIRMIER/AI.. 33<br>MATRONE / ASC .... 34<br>GUERISSEUR /ACCOUCHEUSE<br>TRADITIONNELLE............................ 35<br><br>**AUTRE**<br>CLINIQUE AU LIEU DE TRAVAIL................ 41<br>CENTRE DE JEUNES.............................. 42<br>DISTRIBUTEUR AUTOMATIQUE................ 43<br>CENTRE DE CONSEIL/TEST DE VIH....... 44<br>BAR/CAFE.. 45<br>KIOSQUE/BOUTIQUE/MARCHE.............. 46<br>ONG.. 47<br>VOLONTAIRES/PAIRS ÉDUCATEURS....... 48<br>AMIS.. 49<br>**AUTRE**________________________________ 96<br>(PRECISER)	
Q322	Approuvez-vous l'utilisation de la PF ?	OUI.. 1<br>NON.. 2<br>NE SAIT PAS.. 8	
Q323	**VERIFIEZ Q301 :**<br>**A ENTENDU PARLER DE PRESERVATIFS MASCULINS "7" CODES = 1 OU 2** ☐ ↓	**N'A JAMAIS ENTENDU PARLER DE PRESERVATIFS MASCULINS "7" CODE = 3** ☐ →	**Q330**

Q324	Est-il facile de vous procurer des préservatifs dans votre zone lorsque vous en avez besoin ?	OUI……………………………………………………… 1<br>NON……………………………………………………… 2<br>NE SAIT PAS………………………………………… 8	
Q325	Avez-vous jamais recommandé l'utilisation de préservatifs masculins comme une méthode de PF/Contraception à vos amis ou parents ?	OUI……………………………………………………… 1<br>NON……………………………………………………… 2	
Q326	Si un préservatif masculin est utilisé correctement, pensez-vous qu'il protège contre une grossesse la plupart du temps, seulement quelques fois, ou pas du tout ?	LA PLUPART DU TEMPS…………………………… 1<br>SOUVENT…………………………………………… 2<br>PAS DU TOUT………………………………………… 3<br>NE SAIT PAS/ PAS SUR……………………………… 8	
Q327	Pensez-vous que l'utilisation d'un préservatif réduit le plaisir sexuel de la femme ?	OUI……………………………………………………… 1<br>NON……………………………………………………… 2<br>NE SAIT PAS………………………………………… 8	
Q328	Pensez-vous que l'utilisation d'un préservatif réduit le plaisir sexuel de l'homme?	OUI……………………………………………………… 1<br>NON……………………………………………………… 2<br>NE SAIT PAS………………………………………… 8	
Q329	Pensez-vous que l'utilisation d'un préservatif est un signe d'infidélité?	OUI……………………………………………………… 1<br>NON……………………………………………………… 2<br>NE SAIT PAS………………………………………… 8	

Croyances - Représentations

	S'il vous plait, pouvez vous me dire si vous êtes totalement d'accord, d'accord, pas d'accord ou totalement contre les croyances/les arguments suivants:	ENTIER-MENT D'ACCORD	D'ACCORD	PAS D'ACCORD	TOTALEMENT CONTRE
Q330	L'utilisation des produits contraceptifs rend la femme stérile pour toujours	4	3	2	1
Q331	Les contraceptifs réduisent le désir sexuel des femmes	4	3	2	1
Q332	Les contraceptifs peuvent causer le cancer	4	3	2	1
Q333	Les contraceptifs peuvent vous donner des bébés malformés	4	3	2	1
Q334	Les contraceptifs sont dangereux pour la santé des femmes	4	3	2	1
Q335	La contraception/l'espacement des naissances est une affaire de femme, un homme n'a rien a y voir	4	3	2	1
Q336	Les femmes qui utilisent les méthodes contraceptives peuvent devenir de mœurs légères	4	3	2	1
Q337	Un homme doit accompagner sa femme à l'établissement de santé pour la PF	4	3	2	1
Q338	Les personnes qui utilisent des contraceptifs finissent par avoir des problèmes de santé	4	3	2	1
Q339	Les contraceptifs peuvent faire du mal à l'utérus	4	3	2	1

	MARIAGE ET ACTIVITES SEXUELLES		
	**Questions et filtres**	**Modalités de codage**	**Allez à**
Maintenant, je voudrais vous poser des questions sur votre activité sexuelle et vos relations maritales. Rappelez-vous que vos réponses sont confidentielles			
Q401	Quel âge aviez-vous lorsque vous avez eu un rapport sexuel pour la toute première fois?	AGE……..………………..…………………...... [ ][ ]<br>N'A JAMAIS EU DE RAPPORTS SEXUELS…………..…..……..…. 00	 **Q415**
Q402	La première fois que vous avez eu un rapport sexuel, aviez-vous (ou votre partenaire) utilisé une méthode contraceptive pour eviter une grossesse?	OUI…………………..…….……….……………..……..………..…… 1<br>NON…………………..………..……..……………..…………………… 2<br>NE SE SOUVIENT PAS…………..……..…..…………..………………. 8	 **Q404**<br>**Q404**
Q403	Quelle(s) méthode(s) aviez vous utilisé ?<br><br>INSISTER: D'autres méthodes?<br><br>**ENCERCLER TOUT CE QUI EST MENTIONNE**	STERILISATION FEMININE .... A<br>STERILISATION MASCULINE...................................... B<br>IMPLANT.........................……………………................... C<br>DIU …………………………………….…....................……..……. D<br>INJECTABLES……………………………………...…........…........ E<br>PILULE....………………………………………...............…….. F<br>CONTRACEPTION D'URGENCE…………….……………............…. G<br>PRESERVATIF MASCULIN.. H<br>PRESERVATIF FEMININ... I<br>COLLIER DU CYCLE…………………………………………..…… J<br>ALLAITEMENT AU SEIN /MAMA……………..…………….. K<br>AUTRES MÉTHODES MODERNES…………………………….. L<br>MÉTHODE DU RYTHME………………………………………… M<br>RETRAIT…………………………………………………………..…. N<br>AUTRES MÉTHODES TRADITIONNELLES…….……………………. X	
	Maintenant, je voudrais vous posez des questions sur votre activité sexuelle récente. Laissez-moi vous assurer encore que vos réponses resteront strictement confidentielles et ne seront communiquées à personne. Si nous arrivons à une question à laquelle vous ne souhaitez pas répondre, dites le-moi et nous irons à la question suivante.		
Q404	Quand avez-vous eu vos derniers rapports sexuels?<br><br>SI MOINS D'UNE JOURNÉE, ENREGISTRER « 00 » JOURS.<br>SI MOINS D'UNE SEMAINE, ENREGISTRER JOURS<br>SI MOINS D'UN MOIS, ENREGISTRER SEMAINES<br>SI MOINS DE 12 MOIS, ENREGISTRER MOIS<br>SI 12 MOIS (UNE ANNÉE) OU PLUS, ENREGISTRER EN ANNÉES	IL Y A DES JOURS…………………………..……..… 1 [ ][ ]<br>IL Y A DES SEMAINES………………….……….…. 2 [ ][ ]<br>IL Y A DES MOIS…....…......................…..…… 3 [ ][ ]<br>IL Y A DES ANNEES……......................…..…… 4 [ ][ ]	 <br><br>**Q415**

		**DERNIERE PARTENAIRE**	**AVANT DERNIERE PARTENAIRE**	**AVANT AVANT DERNIERE PARTENAIRE**	
INSTRUCTIONS *:POSEZ Q406, PUIS Q407, Q408 Q409, Q410, Q411, Q412, ET Q413 POUR LA DERNIERE PARTENAIRE AVANT DE CONTINUER AVEC L'AVANT DERNIERE PARTENAIRE PUIS SA PRECEDENTE.*					
Q405	A quand remontent vos derniers rapports sexuels avec cette personne ?		JOURS……… 1 [ ][ ]<br>SEMAINES… 2 [ ][ ]<br>MOIS……….. 3 [ ][ ]	JOURS…….. 1 [ ][ ]<br>SEMAINES… 2 [ ][ ]<br>MOIS……….. 3 [ ][ ]	
Q406	La dernière fois que vous avez eu un rapport sexuel avec cette partenaire, aviez-vous (ou avait-elle) fait quelque chose ou utilisé une méthode pour éviter une grossesse?	OUI…………………… 1<br>NON……..…………. 2 → **Q410**<br>NSP/PAS SÛR……… 8 → **Q410**	OUI………………… 1<br>NON……..………… 2 → **Q410**<br>NSP/PAS SÛR…… 8 → **Q410**	OUI……………………. 1<br>NON……..…………… 2 → **Q410**<br>NSP/PAS SÛR………. 8 → **Q410**	
Q407	Quelle(s) méthode(s) aviez vous utilisé?<br><br>**ENCERCLER TOUT CE QUI EST MENTIONNE**<br>**SI PILULE, INSISTER POUR SAVOIR SI PILULE JOURNALIERE OU CONTRACEPTION D'URGENCE**	STERILISATION FEMININE .... A<br>STERILISATION MASCULINE....... B<br>IMPLANT.........................………… C<br>DIU …………………………………. D<br>INJECTABLES…………………….. E<br>PILULE....……………………………. F<br>CONTRACEPTION D'URGENCE.. G<br>PRESERVATIF MASCULIN............ H<br>PRESERVATIF FEMININ................ I<br>COLLIER DU CYCLE……………… J<br>ALLAITEMENT AU SEIN /MAMA… K<br>AUTRES MÉTHODES MODERNE L<br>MÉTHODE DU RYTHME M<br>RETRAIT…………………………… N<br>AUTRES MÉTHODES TRADITION X	STERILISATION FEMININE .... A<br>STERILISATION MASCULINE. B<br>IMPLANT.............................… C<br>DIU …………………………… D<br>INJECTABLES………………. E<br>PILULE....……………………… F<br>CONTRACEPTION D'URGEN(G<br>PRESERVATIF MASCULIN..... H<br>PRESERVATIF FEMININ......... I<br>COLLIER DU CYCLE………… J<br>ALLAITEMENT AU SEIN /MAM K<br>AUTRES MÉTHODES MODEF L<br>MÉTHODE DU RYTHME M<br>RETRAIT……………………… N<br>AUTRES MÉTHODES TRADIT X	STERILISATION FEMININE .... A<br>STERILISATION MASCULINE.... B<br>IMPLANT...................................… C<br>DIU ………………………………. D<br>INJECTABLES…………………… E<br>PILULE....……………………….… F<br>CONTRACEPTION D'URGENCE G<br>PRESERVATIF MASCULIN........ H<br>PRESERVATIF FEMININ............ I<br>COLLIER DU CYCLE…………… J<br>ALLAITEMENT AU SEIN /MAMA K<br>AUTRES MÉTHODES MODERN L<br>MÉTHODE DU RYTHME M<br>RETRAIT…………………………. N<br>AUTRES MÉTHODES TRADITIC X	

		DERNIERE PARTENAIRE	AVANT DERNIERE PARTENAIRE	AVANT AVANT DERNIERE PARTENAIRE	
Q408	***VERIFIEZ Q407: POUR LES ENQUETES QUI N'ONT PAS UTILISE LE PRESERVATIF, ALLER A Q411***	***A UTILISE UNE METHODE AUTRE QUE LE PRESERVATIF*** ☐ → **Q411**<br><br>***PRESERVATIF MASCULIN OU FEMININ (Q407 = "H" OU "I")*** ☐ ↓	***A UTILISE UNE METHODE AUTRE QUE LE PRESERVATIF*** ☐ → **Q411**<br><br>***PRESERVATIF MASCULIN OU FEMININ (Q407 = "H" OU "I")*** ☐ ↓	***A UTILISE UNE METHODE AUTRE QUE LE PRESERVATIF*** ☐ → <br>***PRESERVATIF MASCULIN OU FEMININ (Q407 = "H" OU "I")*** ☐ ↓	**Q411**
Q409	**PRESERVATIF AU DERNIER RAPPORT SEXUEL:**<br>Quelle était la principale raison pour laquelle vous avez utilisé un préservatif à cette occasion-là?	EVITER MALADIES.................. 1<br>EVITER GROSSESSE.............. 2<br>EVITER A LA FOIS .... 3<br>N'A PAS CONFIANCE EN LA PARTENAIRE............ 4<br>LA PARTENAIRE L'A EXIGE / A INSISTE.............................. 5<br>AUTRE_____________________ 6<br>(A PRECISER) **Q411**	EVITER MALADIES............ 1<br>EVITER GROSSESSE........ 2<br>EVITER A LA FOIS .... 3<br>N'A PAS CONFIANCE EN LA PARTENAIRE...... 4<br>LA PARTENAIRE L'A EXIGE / A INSISTE............... 5<br>AUTRE________________ 6<br>(A PRECISER) **Q411**	EVITER MALADIES.......... 1<br>EVITER GROSSESSE...... 2<br>EVITER A LA FOIS .... 3<br>N'A PAS CONFIANCE EN LA PARTENAIRE..... 4<br>LA PARTENAIRE L'A EXIGE / A INSISTE....................... 5<br>AUTRE________________ 6<br>(A PRECISER)	→**Q411**
Q410	Quelle est la principale raison pour laquelle vous n'avez pas utilisé une méthode (OU NE SAIT PAS QU'UNE METHODE A ÉTÉ UTILISEE)cette fois là?	PARTENAIRE OCCASIONNELLE, N'AS PAS FAIT ATTENTION... 11<br>LA CONTRACEPTION EST UNE AFFAIRE DE FEMME............ 12<br><br>FEMME MENOPAUSEE............ 21<br>COUPLE STERILE................... 22<br>FEMME ENCEINTE.................. 23<br>FEMME AMENORRHEIQUE...... 24<br>FEMME ALLAITANTE............... 25<br>DESIR D'ENFANT.................... 26<br><br>ENQUÊTE OPPOSE A LA PF..... 31<br>FEMME OPPOSEE A LA PF....... 32<br>LES AUTRES S'OPPOSENT...... 33<br>INTERDICTION RELIGIEUSE..... 34<br><br>NE CONNAIT PAS DE METHODI 41<br>NE CONNAIT PAS D'ENDROIT... 42<br><br>PROBLEMES DE SANTE.......... 51<br>CRAINT EFFETS SECONDAIRE! 52<br>PROBLEME D'ACCES/TROP LO 53<br>COÛT TROP ELEVE................. 54<br>PAS FACILE A UTILISER........... 55<br>INFLUENCE LE PROCESSUS NORMAL DE CROISSANCE... 56<br><br>AUTRE___________________ 96<br>(A PRECISER)<br>NSP.. 98	PARTENAIRE OCCASIONNELLE N'AS PAS FAIT ATTENTI(11<br>LA CONTRACEPTION EST UNE AFFAIRE DE FEMME...... 12<br><br>FEMME MENOPAUSEE...... 21<br>COUPLE STERILE............. 22<br>FEMME ENCEINTE............ 23<br>FEMME AMENORRHEIQUE 24<br>FEMME ALLAITANTE......... 25<br>DESIR D'ENFANT.............. 26<br><br>ENQUÊTE OPPOSE A LA PI 31<br>FEMME OPPOSEE A LA PF 32<br>LES AUTRES S'OPPOSENT 33<br>INTERDICTION RELIGIEUSI 34<br><br>NE CONNAIT PAS DE METH 41<br>NE CONNAIT PAS D'ENDR(42<br><br>PROBLEMES DE SANTE.... 51<br>CRAINT EFFETS SECONDA 52<br>PROBLME D'ACCES/TROP 53<br>COÛT TROP ELEVE........... 54<br>PAS FACILE A UTILISER..... 55<br>INFLUENCE LE PROCESSUS NORMAL DE CROISSAN(56<br><br>AUTRE________________ 96<br>(A PRECISER)<br>NSP.................................. 98	PARTENAIRE OCCASIONNELLE, N'AS PAS FAIT ATTENTION.. 11<br>LA CONTRACEPTION EST UNE AFFAIRE DE FEMME........... 12<br><br>FEMME MENOPAUSEE........... 21<br>COUPLE STERILE.................. 22<br>FEMME ENCEINTE................. 23<br>FEMME AMENORRHEIQUE..... 24<br>FEMME ALLAITANTE.............. 25<br>DESIR D'ENFANT................... 26<br><br>ENQUÊTE OPPOSE A LA PF.... 31<br>FEMME OPPOSEE A LA PF...... 32<br>LES AUTRES S'OPPOSENT..... 33<br>INTERDICTION RELIGIEUSE.... 34<br><br>NE CONNAIT PAS DE METHOD 41<br>NE CONNAIT PAS D'ENDROIT.. 42<br><br>PROBLEMES DE SANTE......... 51<br>CRAINT EFFETS SECONDAIRE 52<br>PROBLEME D'ACCES/TROP LC 53<br>COÛT TROP ELEVE................. 54<br>PAS FACILE A UTILISER.......... 55<br>INFLUENCE LE PROCESSUS NORMAL DE CROISSANCE.. 56<br><br>AUTRE__________________ 96<br>(A PR (A PRECISER)<br>NSP....................................... 98	
Q411	Quelle est votre lien/relation avec cette femme?<br><br>SI LA FEMME EST "FIANCÉE" OU "PETITE AMIE", DEMANDEZ:<br>Votre fiancée ou petite amie vivait-elle avec vous quand vous aviez eu votre dernier rapport sexuel?<br><br>SI LA REPONSE EST "OUI", REPORTEZ "01" ("EPOUSE"); SI "NON", REPORTEZ "02" (FIANCEE) OU "03" (PETITE AMIE)	EPOUSE/CONCUBINE............. 01<br>FIANCÉE.................................. 02<br>PETITE AMIE .... 03<br>AUTRE AMIE .... 04<br>PARTENAIRE OCCASIONNELLE 05<br>PARTENAIRE COMMERCIALE / TRAVAILLEUSE DE SEXE. 06<br>EX-FEMME............................. 07<br>EX-PETITE AMIE .... 08<br>AUTRE___________________ 96<br>(A PRECISER)	EPOUSE/CONCUBINE.......... 01<br>FIANCÉE................................ 02<br>PETITE AMIE .... 03<br>AUTRE AMIE .... 04<br>PARTENAIRE OCCASIONNEL 05<br>PARTENAIRE COMMERCIALE / TRAVAILLEUSE DE SEX 06<br>EX-FEMME.......................... 07<br>EX-PETITE AMIE .... 08<br>AUTRE___________________ 96<br>(A PRECISER)	EPOUSE/CONCUBINE............. 01<br>FIANCÉE.................................. 02<br>PETITE AMIE .... 03<br>AUTRE AMIE .... 04<br>PARTENAIRE OCCASIONNELLI 05<br>PARTENAIRE COMMERCIALE / TRAVAILLEUSE DE SEXE. 06<br>EX-FEMME............................ 07<br>EX-PETITE AMIE .... 08<br>AUTRE__________________ 96<br>(A PRECISER)	
Q412	Quelle a été la durée de la relation avec cette femme?<br><br>SI A EU RAPPORT SEXUEL UNE SEULE FOIS AVEC CETTE FEMME, NOTEZ "01"JOURS.	JOURS.......... 1<br>SEMAINES.... 2<br>MOIS............ 3<br>ANNEES........ 4	JOURS...... 1<br>SEMAINES. 2<br>MOIS......... 3<br>ANNEES.... 4	JOURS..... 1<br>SEMAINES 2<br>MOIS........ 3<br>ANNEES... 4	
Q413	A part cette partenaire avez vous eu des rapports sexuels avec d'autres personnes au cours des 12 derniers mois?	OUI......1 Retourner à Q405 (Colonne suivante)<br><br>Non......2 → **Q414**	OUI......1 Retourner à Q405 (Colonne suivante)<br><br>Non......2 → **Q414**		

Q414	Au total, avec combien de femmes avez-vous eu un rapport sexuel au cours des 12 derniers mois?<br><br>ASSUREZ-VOUS DE LA COHERENCE ENTRE LE NOMBRE DE PARTENAIRES MENTIONNES ICI ET LE TABLEAU PRECEDENT	NOMBRE DE FEMMES……………… [ ][ ]<br>PLUS DE 95 FEMMES …………… 95	
	Maintenant, je voudrais vous poser des questions sur votre vie conjugale. Rappelez-vous que vos réponses sont confidentielles		
Q415	Avez-vous déjà été marié ou avez-vous déjà vécu avec une femme ?	OUI,A ETE MARIE………………… 1<br>OUI, A VECU AVEC UNE FEMME………… 2<br>NON ……………………… 3	NON → Q501
Q416	Quel est votre statut matrimonial actuel. Êtes-vous actuellement marié, ou vivez vous avec une femme, veuf, divorcé ou séparé ?	ACTUELLEMENT MARIE………………… 1<br>VIT AVEC UNE FEMME…………… 2<br>VEUF……………………… 3<br>DIVORCE…………………… 4<br>SEPARE…………………… 5	3-5 → Q420
Q417	Votre/vos femme (s)/partenaire(s) vit/vivent-elle(s) actuellement avec vous, où habite(ent)-elle(s) ailleurs?	VIT AVEC PARTENAIRE/EPOUSE………… 1<br>EPOUSE/PARTENAIRE HABITE AILLEURS……… 2	
Q418	Avez-vous plus d'une épouse ou femme avec qui vous vivez maritalement ?	OUI…………………… 1<br>NON…………………… 2	NON → Q420
Q419	En tout, avec combien d'épouses ou d'autres partenaires vivez-vous de manière maritale (ou comme si vous êtiez marié)?	NOMBRE TOTAL D'EPOUSES/<br>DE PARTENAIRES:……………… [ ][ ]	
Q420	Avez-vous été marié une seule fois ou plus d'une fois/ avez-vous vécu avec une seule femme ou avec plus d'une femme?	SEULEMENT UNE FOIS………………… 1<br>PLUS D'UNE FOIS ……………… 2	
Q421	***VERIFIEZ Q420:***<br><br>A ÉTÉ MARIE UNE SEULE FOIS/A VECU AVEC UNE SEULE FEMME [ ] → En quel mois et en quelle année avez-vous commencé à vivre avec votre femme?<br><br>A ÉTÉ MARIE PLUS D'UNE FOIS/A VECU AVEC PLUS D'UNE FEMME [ ] → En quel mois et année aviez-vous commencé à vivre avec votre première partenaire ou épouse?	MOIS………………… [ ][ ]<br>NE CONNAIT PAS LE MOIS ………… 98<br>ANNEE……………… [ ][ ][ ][ ]<br>NE CONNAIT PAS L'ANNEE ………… 9998	

SECTION 5: PRÉFÉRENCES EN MATIÈRE DE FÉCONDITÉ

	Questions et filtres	Modalités de codage	Allez à
Q501	**VÉRIFIEZ 302 : (02) ; STÉRILISATION MASCULINE**<br>HOMME **PAS STÉRILISÉ** ☐ ↓	HOMME STÉRILISÉ (Q302 (02) = 1) ☐ →	Q507
Q502	**VÉRIFIEZ Q415 & Q416 = STATUT MARITAL**<br>ACTUELLEMENT MARIE OU VIT EN UNION (Q416=1 OU 2) ☐ ↓	PAS EN UNION (Q415=3 OU Q416=3, 4 OU 5) ☐ →	Q505
Q503	Votre épouse/partenaire (ou une de vos épouses/ partenaires) est-elle actuellement enceinte ?	OUI …………………………………… 1<br>NON……………………………………… 2<br>NE SAIT PAS ……………………………… 8	 Q505<br>Q505
Q504	Lorsque votre épouse/partenaire est tombée enceinte si vous aviez le choix, auriez vous souhaité qu'elle tombe enceinte à ce moment-là, qu'elle attende plus tard ou ne pas avoir un (autre) enfant du tout ?	A CE MOMENT-LA……………………… 1<br>PLUS TARD………………………………… 2<br>NE PAS AVOIR D'(AUTRE) ENFANT……... 3	
Q505	**VÉRIFIEZ Q502 ET Q503:**<br>Maintenant, j'ai des questions concernant votre futur.<br>PAS D'ÉPOUSE OU PARTENAIRE /PARTENAIRE N'EST PAS ENCEINTE OU PAS SÛRE SI ELLE EST ENCEINTE ☐ ↓<br>Voudriez-vous avoir un (autre) enfant ou préfèreriez-vous ne pas avoir d'(autres) enfants?<br>FEMME/PARTENAIRE ENCEINTE ☐ ↓<br>Après l'enfant que votre épouse/partenaire attend, voudriez-vous avoir un autre enfant, ou préfèreriez vous ne pas avoir d'autre enfant du tout?	AVOIR UN (UN AUTRE) ENFANT…………. 1<br>NE PAS AVOIR D'AUTRE ENFANT……..… 2<br>FEMME/FEMMES TOUTE(S) INFÉCONDE(S) / STÉRILISÉE(S)………… 3<br>ENQUÊTÉ INFÉCOND……………………… 4<br>INDÉCIS / NE SAIT PAS……………………… 8	 Q507 (2, 3, 4, 8)
Q506	**VÉRIFIEZ Q503**<br>PAS ENCEINTE OU PAS SURE D'ÊTRE ENCEINTE ☐ ↓<br>Combien de temps voudriez-vous attendre à partir de maintenant avant la naissance d'un (autre) enfant ?<br>ENCEINTE ☐ ↓<br>Après la naissance de l'enfant que vous attendez, combien de temps voudriez-vous attendre pour avoir un autre enfant ?<br>SI LA RÉPONSE EST UN NOMBRE DÉCIMAL DE MOIS OU D'ANNÉES, CONVERTIR LE TOUT EN MOIS ET REPORTER DANS LA CASE "MOIS" (PAR EXEMPLE, 2 1/2 ANS=30 MOIS)	MOIS……………………… 1 ☐☐<br>ANNÉES …………………… 2 ☐☐<br>BIENTÔT/MAINTENANT……………… 993<br>FEMME NE PEUT PAS AVOIR D'ENFANTS……………………… 994<br>APRÈS MARIAGE ……………………. 995<br>AUTRE ____________________ 996<br>(PRÉCISER)<br>NE SAIT PAS …………………………… 998	 <br><br><br>Q511 (995)
Q507	ACTUELLEMENT MARIE VIT EN UNION (Q416=1 OU 2) ☐ ↓	PAS EN UNION (Q415=3 OU Q416=3, 4 OU 5) ☐ →	Q511
Q508	Combien d'enfants pensez-vous que votre femme (toutes vos femmes ensemble) voudrait(ent)-elle(s) avoir dans la vie ?<br>**SI AUCUN REPORTEZ "00"**	NOMBRE D'ENFANTS……. ☐☐<br>NE SAIT PAS ………………………………… 98	
Q509	Diriez-vous que ……..<br>Vous voudriez avoir le même nombre d'enfants qu'elle(s) ou plus ou bien moins d'enfants qu'elle(s) ?	MÊME NOMBRE……………………………… 1<br>PLUS D'ENFANTS…………………………… 2<br>MOINS D'ENFANTS………………………… 3<br>NE SAIT PAS ……………………………… 8	
Q510	Qui décide du nombre d'enfants que vous allez avoir -diriez-vous que cette décision vous appartient principalement, appartient principalement à votre femme/partenaire, ou avez-vous décidé tous les deux ensemble ?	PRINCIPALEMENT VOUS………………… 1<br>PRINCIPALEMENT VOTRE PARTENAIRE………… 2<br>CONJOINTEMENT……………………… 3<br>AUTRE____________________ 6<br>(PRECISER)	

Q511	**VÉRIFIEZ Q202 & Q204:**<br>**INSISTEZ POUR AVOIR UNE RÉPONSE NUMÉRIQUE**<br>A DES ENFANTS EN VIE ☐ → Si vous pouviez retourner à l'époque où vous n'aviez pas encore d'enfants et où vous pouviez choisir exactement le nombre d'enfants à avoir dans toute votre vie, combien auriez-vous voulu en avoir?<br>PAS D'ENFANTS EN VIE ☐ → Si vous pouviez choisir exactement le nombre d'enfants que vous voulez toute votre vie, combien auriez-vous voulu?	AUCUN .... 00<br>NOMBRE... ☐☐<br>AUTRE_________________________ 96<br>(PRÉCISEZ)	→ **Q513**<br><br>→ **Q513**
Q512	Parmi ces enfants, combien souhaiteriez-vous de garçons, combien souhaiteriez-vous de filles, et pour combien d'entre eux, le sexe n'aurait-il pas d'importance ?	GARçONS FILLES INDIFFERENT<br>NOMBRE ☐☐ ☐☐ ☐☐	
Q513	Seriez-vous prêt à utiliser une méthode de PF en vue d'avoir exactement ce nombre d'enfants ?	OUI .... 1<br>NON.. 2	
	Maintenant je voudrais vous demandez vos opinions concernant la grossesse et la naissance.		
Q514	Selon vous, combien de temps un couple doit il attendre après le mariage pour avoir leur premier enfant?	IMMEDIATEMENT.. 1<br>MOINS D'UN AN.. 2<br>ENTRE UN AN ET AVANT 2 ANS APRES MARIAGE.. 3<br>2 OU PLUSIEURS ANNEES.. 4<br>AUTRE_______________________________ 6<br>(PRECISER)	
Q515	Selon vous, quel devrait être l'âge idéal en années pour une femme de tomber enceinte ou avoir un enfant pour la première fois ?	ÂGE EN ANNEES .... ☐☐<br>NE SAIT PAS 98	
Q516	Selon vous, quel devrait être l'âge idéal en années pour un homme d'être pour la première fois pére d'un enfant ?	ÂGE EN ANNEES .... ☐☐<br>NE SAIT PAS 98	
Q517	A votre avis, quel devrait être l'intervalle d'âge idéal entre deux enfants?<br>SI LA REPONSE EST UN NOMBRE DECIMAL DE MOIS OU D'ANNES, CONVERTIR LE TOUT EN MOIS ET REPORTER DANS LA CASE "MOIS" (PAR EXEMPLE, 2 1/2 ANS=30 MOIS)	MOIS.. 1 ☐☐<br>OU<br>ANNEES.. 2 ☐☐<br>NE SAIT PAS 998	
Q518	Selon vous, quel serait le nombre idéal d'enfants pour un couple qui a les moyens financiers?<br><br>INSISTER POUR AVOIR UNE REPONSE NUMERIQUE.	AUCUN.. 00<br>NOMBRE.. ☐☐<br>AUTRE___________________________________ 96<br>(PRECISER)	

Q519	Quels sont les effets (positifs et/ou négatifs) sur la mère si elle utilise une méthode de PF ou d'espacement des naissances ?<br><br>INSISTER: D'autres effets?<br><br>ENREGISTRER TOUT CE QUI EST MENTIONNE.	MEILLEUR ETAT NUTRITIONNEL.................... A<br>INCIDENCE D'ANEMIE PLUS FAIBLE................ B<br>MOINS DE COMPLICATIONS DE GROSSESSE.. C<br>PREVENIR IST /VIH.. D<br>PREVENIR GROSSESSES INDESIRABLES........ E<br>MOINS D'ENFANT A EDUQUER....................... F<br>LA FEMME A PLUS DE TEMPS LIBRE............... G<br>LA FAMILLE GAGNE PLUS D'ARGENT.............. H<br>ESPRIT TRANQUILLE.. I<br>BONHEUR FAMILIAL /ENTENTE DANS<br>LE COUPLE.. J<br>MEILLEURE SANTE DE LA MERE..................... K<br>PROBLEMES DE COUPLE/DISPUTES/DIVORCE L<br>REJET COMMUNAUTE/PROCHES.................... M<br>PROBLEMES DE CYCLE................................. N<br>PROBLEMES DE SANTE................................. O<br>GAIN DE POIDS... P<br>PERTE DE POIDS... Q<br>AFFAIBLISSEMENT.. R<br>RETOUR TARDIF DE FECONDITE S<br>AUTRE_________________________ X<br>(PRECISER)<br>AUCUN... Y	
Q520	Quels sont les effets (positifs/négatifs) sur l'enfant si la mère utilise une méthode de PF ou d'espacement des naissances ?<br><br>INSISTER: D'autres effets?<br><br>NE PAS LIRE LES REPONSES.	MEILLEURE CROISSANCE.............................. A<br>MEILLEUR ETAT NUTRITIONNEL.................... B<br>MEILLEURE SANTE.. C<br>MEILLEURE CHANCE DE SURVIE.................... D<br>PLUS GRANDE ATTENTION DE LA MERE......... E<br>MEILLEURE EDUCATION................................. F<br>PLUS FAIBLE INCIDENCE DES MALADIES........ G<br>PLUS D'OPPORTUNITES DANS LA VIE............. H<br>MALFORMATIONS... I<br>PROBLEMES DE CROISSANCE...................... J<br>OBESITE.. K<br>PROBLEMES NUTRITIONNELS........................ L<br>MALADIES FREQUENTES................................ M<br>NAISSANCES MULTIPLES/JUMEAUX................ N<br>AUTRE_________________________ X<br>(PRECISER)<br>AUCUN... Y	
Q521	**VÉRIFIEZ Q415 ET Q416: DÉJÀ MARIE ET ACTUELLEMENT MARIE**<br>ACTUELLEMENT MARIE (Q416=1 OU 2) ☐ ↓	PAS EN UNION (Q415=3 OU (Q416=3, 4 OU 5) ☐	→ **Q601**
Q522	Avez-vous jamais accompagné votre épouse (ou votre enfant) dans une structure de santé ?	OUI .... 1<br>NON.. 2	 → **Q601**
Q523	A quelle fréquence accompagnez-vous votre épouse (ou enfant) dans une structure sanitaire ?	RAREMENT.. 1<br>QUELQUEFOIS....................................... 2<br>SOUVENT... 3<br>TOUJOURS... 4	

SECTION 6: COMMUNICATION ENTRE ÉPOUSE ET INTERPERSONNELLE			
Q601	*Je voudrais vous demander quelques questions sur les discussions que vous auriez eues avec d'autres personnes au sujet des problèmes de santé.*<br>VÉRIFIEZ Q415 & Q416:<br>MARIE OU VIT MARITALEMENT AVEC UNE FEMME (Q416=1 OU 2) ☐ ↓	PAS EN UNION (Q415=3 OU Q416=3, 4 OU 5) ☐ →	Q609
Q602	Avez-vous, vous et votre épouse/partenaire, déjà discuté du nombre d'enfants que vous aimeriez avoir ?	OUI .... 1<br>NON 2	 → Q604
Q603	Quelle est la fréquence avec laquelle vous avez parlé à votre épouse/partenaire de ce sujet au cours des 6 derniers mois?	NOMBRE DE FOIS........ ☐☐<br>PAS DISCUTE DU TOUT AU COURS DES SIX (6) DERNIERS MOIS........ 97	
Q604	Avez-vous déjà discuté de PF/l'utilisation de la contraception avec votre épouse/partenaire ?	OUI .... 1<br>NON 2	 → Q608
Q605	Quelle est la fréquence avec laquelle vous avez parlé à votre épouse/partenaire de ce sujet au cours des 6 derniers mois?	NOMBRE DE FOIS........ ☐☐<br>PAS DISCUTE DU TOUT AU COURS DES SIX (6) DERNIERS MOIS........ 97	
Q606	D'habitude, qui d'entre-vous commence la discussion sur la PF, vous ou votre épouse/partenaire ?	MOI-MÊME........ 1<br>ÉPOUSE/PARTENAIRE........ 2<br>L'UN OU L'AUTRE........ 3	
Q607	Quel est le degré de difficulté à commencer une conversation sur l'espacement des naissances avec votre partenaire – très difficile, quelque peu difficile, facile ?	TRÈS DIFFICILE........ 1<br>QUELQUE PEU DIFFICILE........ 2<br>FACILE........ 3	
Q608	Avez-vous l'intention de parler à votre épouse de la contraception au cours des trois prochains mois ?	OUI .... 1<br>NON .... 2<br>NE SAIT PAS........ 8	
Q609	Pensez-vous que les autorités gouvernementales / élus locaux devraient parler publiquement de PF/ Contraception ?	OUI........ 1<br>NON........ 2<br>NE SAIT PAS........ 8	
Q610	Au cours des 12 derniers mois, avez-vous entendu ou vu un responsable gouvernemental / élus locaux parler publiquement <u>contre</u> la planification familiale ?	OUI .... 1<br>NON .... 2<br>NE SE RAPPELLE PAS........ 8	
Q611	Au cours des 12 derniers mois, avez-vous entendu ou vu un responsable gouvernemental / élus locaux parler publiquement <u>en faveur</u> de la planification familiale?	OUI .... 1<br>NON .... 2<br>NE SE RAPPELLE PAS........ 8	
Q612	Pensez-vous que les responsables communautaires ou religieux devraient parler publiquement de PF/ Contraception ?	OUI........ 1<br>NON........ 2<br>NE SAIT PAS........ 8	
Q613	Au cours des 12 dernièrs mois, avez-vous entendu ou vu un responsable communautaire ou religieux parler publiquement <u>contre</u> la planification familiale ?	OUI .... 1<br>NON .... 2<br>NE SE RAPPELLE PAS........ 8	
Q614	Au cours des 12 dernièrs mois, avez-vous entendu ou vu un responsable communautaire ou religieux parler publiquement <u>en faveur</u> de la planification familiale?	OUI .... 1<br>NON .... 2<br>NE SE RAPPELLE PAS........ 8	

Q615	Combien parmi vos proches amis/ les membres de votre famille diriez-vous qu'ils utilisent la PF/ Espacement des naissances: aucun, un peu, la plupart ou tous ?	AUCUN………… 1<br>PEU D'ENTRE-EUX………… 2<br>LA PLUPART………… 3<br>TOUS………… 4<br>NE SAIT PAS………… 8	
Q616	Pensez-vous qu'il existe des membres de votre communauté qui vous traiteront de mauvaise personne ou fuiront votre compagnie s'ils savaient que vous utilisez une méthode de PF/Espacement des naissances ?	OUI………… 1<br>NON………… 2<br>NE SAIT PAS………… 8	
Q617	Pensez-vous qu'il existe des membres de votre communauté qui vont te féliciter, t'encourager ou dire du bien de toi s'ils savaient que vous utilisez une méthode de PF/Espacement des naissances ?	OUI………… 1<br>NON………… 2<br>NE SAIT PAS………… 8	

	S'il vous plait, pouvez vous me dire si vous êtes totalement d'accord, d'accord, pas d'accord ou totalement contre les arguments suivants:	TOTALEMENT D'ACCORD	D'ACCORD	PAS D'ACCORD	TOTALEMENT CONTRE
Q618	Vous pouvez initier une conversation sur la PF avec votre partenaire/épouse.	4	3	2	1
Q619	Vous pouvez convaincre votre partenaire que vous devez utiliser une méthode de PF.	4	3	2	1
Q620	Vous pouvez aller à un endroit où la PF est vendue/ offerte pour obtenir une méthode si vous décidez d'en avoir une.	4	3	2	1
Q621	Vous pouvez obtenir une méthode de PF si vous décidez d'en avoir une.	4	3	2	1
Q622	Vous pouvez utiliser une méthode de PF même si votre partenaire ne le veut pas.	4	3	2	1
Q623	Vous pouvez utiliser une méthode de PF même si aucun de vos amis ou voisins n'en utilise.	4	3	2	1
Q624	Vous pouvez utiliser une méthode de PF même si votre leader religieux pense que vous ne devrez pas l'utiliser.	4	3	2	1

SECTION 7: EXPOSITION AUX MEDIA

Maintenant, je voudrais vous parler de vos besoins en informations et des endroits où obtenir des informations sur la PF.

Q701	Quelles sont vos principales sources d'informations sur la santé ?<br><br>**INSISTER SEPAREMENT POUR :**<br>A. SOURCES MEDIATIQUES<br>B. SOURCES MEDICALES<br>C. SOURCES COMMUNAUTAIRES<br>D. SOURCES INTERPERSONNELLES<br><br>ENREGISTRER TOUT CE QUI EST MENTIONNE.	**SOURCES MEDIATIQUES**<br>RADIO.......... AA<br>TV.......... AB<br>JOURNAUX.......... AC<br>MAGAZINES.......... AD<br>PANNEAUX D'AFFICHAGE.......... AE<br>PEINTURE MURALE.......... AF<br>INTERNET .... AG<br>FLYERS/DEPLIANTS.......... AH<br>NUMERO VERT.......... AI<br><br>**SOURCES MEDICALES**<br>MEDECIN.......... BA<br>SAGE-FEMME.......... BB<br>INFIRMIER/AI.......... BC<br>MATRONE / ASC .... BD<br>GUERISSEUR / ACCOUCHEUSE TRADITIONNELLE.......... BE<br>PHARMACIEN .... BF<br><br>**SOURCES COMMUNAUTAIRES**<br>RELAI COMMUNAUTAIRE/BAJENN GOKH .... CA<br>ORGANISATION COMMUNAUTAIRE DE BASE (OCB).......... CB<br>VOLONTAIRES / PAIRS ÉDUCATEURS .... CC<br>ECOLE.......... CD<br>COMMUNICATEUR TRADITIONNEL/RELIGIEUX.......... CE<br><br>**SOURCES INTERPERSONNELLES**<br>PARENTS.......... DA<br>BEAUX-PARENTS.......... DB<br>EPOUX/PARTENAIRE.......... DC<br>FRERES ET SŒURS.......... DD<br>BELLES SŒURS/BEAUX-FRERES.......... DE<br>AMIS / VOISINS.......... DF<br>AUTRES PARENTS/CONNAISSANCES.......... DG<br><br>**AUTRE (PRECISER)**__________ XX<br>**AUCUN..........** YY<br>**NE SAIT PAS ....** ZZ	
Q702	Lisez-vous des journaux ?	OUI .... 1<br>NON 2	
Q703	Lisez-vous des magazines?	OUI .... 1<br>NON 2	
Q704	**VERIFIER: Q702 ET 703**<br>SI OUI A L'UNE OU L'AUTRE : ☐ ↓	SI NON AUX DEUX : ☐ →	Q707

Q705	Avez-vous lu des informations sur la PF dans les journaux/magazines au cours des <u>3 derniers mois</u> ?	OUI .... 1<br>NON 2	 → Q707
Q706	Quelles informations avez-vous lu dans les journaux/magazines sur la planification familiale?<br><br>INSISTER: D'autres informations?<br><br>ENCERCLER TOUT CE QUI EST MENTIONNE.<br><br>SI PILULE, INSISTER POUR SAVOIR SI PILULE JOURNALIERE OU CONTRACEPTION D'URGENCE.	PILULES........ A<br>DIU........ B<br>PRESERVATIFS........ C<br>INJECTABLES .... D<br>IMPLANT........ E<br>CONTRACEPTION D'URGENCE........ F<br>STERILISATION FEMININE........ G<br>STERILISATION MASCULINE........ H<br>ALLAITEMENT MATERNEL........ I<br>METHODE DES JOURS FIXES........ J<br>AGE DU MARIAGE........ K<br>RETARDER L'AGE DU 1ER RAPPORT SEXUEL........ L<br>RETARDER LA PREMIERE NAISSANCE........ M<br>ESPACEMENT DES NAISSANCES/MOYTU NEF........ N<br>LIMITATION DE LA TAILLE DE LA FAMILLE........ O<br>BIEN-FONDÉ DE LA PF........ P<br>COMMUNICATION ENTRE EPOUX........ Q<br>AUTORITE GOUVERMENTALE PARLER DE PF........ R<br>DISCOURS D'UN LEADER RELIGIEUX SUR LA PF........ S<br>RUMEURS ET CRAINTES SUR LA PF........ T<br>AUTRES________ X<br>(PRECISER)	
Q707	Ecoutez-vous la radio?	OUI .... 1<br>NON 2	 → Q713
Q708	Quelles stations de radio écoutez-vous?<br><br>INSISTER: quelles autres stations radio?<br><br>ENCERCLER TOUT CE QUI EST MENTIONNE.	RTS........ A<br>WALF FM........ B<br>SUD FM/SUD FM KL........ C<br>DUNYA FM/DOUNYA MBOUR........ D<br>RFM........ E<br>ZIK FM........ F<br>OXYJEUNES........ G<br>X-FM........ H<br>AFIA FM........ I<br>ALFAIDA........ J<br>NDEFLENG........ K<br>SOXNA FM........ L<br>DJIDA FM........ M<br>JOKKO FM........ N<br>MBOUR FM........ O<br>LAMP FALL FM........ P<br>RAIL BI FM........ Q<br>ALMADINA FM........ R<br>RFI........ S<br>MOZDAIR........ T<br>RADIO RENNDO........ U<br>RSI........ V<br>AUTRE________ X<br>(PRECISER)	
Q709	Combien de jours par semaine écoutez-vous la radio?	NOMBRE DE JOURS PAR SEMAINE........ [ ]<br>PAS REGULIEREMENT........ 8	

Q710	En moyenne combien de temps au total écoutez-vous la radio dans un jour normal ?	HEURES PAR JOUR.................................1 [0][ ][ ]<br>OU<br>MINUTES PAR JOUR..............................2 [ ][ ][ ]	
Q711	Avez-vous entendu des informations sur la PF à la radio au cours des trois derniers mois?	OUI...1<br>NON...2	 → Q716
Q712	Quelles informations avez-vous entendu à la radio sur la planification familiale?<br><br>INSISTER: D'autres informations?<br><br>ENCERCLER TOUT CE QUI EST MENTIONNE.<br><br>SI PILULE, INSISTER POUR SAVOIR SI PILULE JOURNALIERE OU CONTRACEPTION D'URGENCE.	PILULES...A<br>DIU...B<br>PRESERVATIFS...C<br>INJECTABLES ....D<br>IMPLANT..E<br>CONTRACEPTION D'URGENCE..............................F<br>STERILISATION FEMININE..................................G<br>STERILISATION MASCULINE...............................H<br>ALLAITEMENT MATERNEL..................................I<br>METHODE DES JOURS FIXES..............................J<br>AGE DU MARIAGE..K<br>RETARDER L'AGE DU 1ER RAPPORT SEXUEL.......................L<br>RETARDER LA PREMIERE NAISSANCE...............................M<br>ESPACEMENT DES NAISSANCES/MOYTU NEF....................N<br>LIMITATION DE LA TAILLE DE LA FAMILLE..........................O<br>BIEN-FONDÉ DE LA PF..P<br>COMMUNICATION ENTRE EPOUX......................................Q<br>AUTORITE GOOUVERMENTALE PARLER DE PF..................R<br>DISCOURS D'UN LEADER RELIGIEUX SUR LA PF................S<br>RUMEURS ET CRAINTES SUR LA PF.................................T<br>AUTRES_______________________________________X<br>(PRECISER)	

Q713	Vous arrive-t-il d'ecouter/suivre un des programmes suivants ?	OUI TOUJOURS/ SOUVENT	OCCASION NELLEMENT	NON JAMAIS	N'ECOUTE PAS CES RADIOS	NE CONNAIT PAS CETTE EMISSION
	a. Emission santé sur Rail Bi FM, Oxyjeunes Afia FM, Mozdair, Jokko rufisque, dounya, Sud FM Renndo, Lamp fall, Mbour FM ou Alfayda ?...........	1	2	3	7	8
	b. Emission Religieuse sur Rail Bi FM, Oxyjeunes Afia FM, Jokko rufisque, dounya, Sud FM KL, Renndo, Lamp fall, Mbour FM ou Alfayda ?..........	1	2	3	7	8
	c. Emission musicale sur Rail Bi FM, Oxyjeunes Afia FM, Jokko rufisque, dounya, Sud FM KL Mbour FM ou Alfayda ?....................................	1	2	3	7	8
	d. Emission interactive Loci Xam sur Sud FM ?......	1	2	3	7	8
	e. Emission Xam sa yaram sur Lamp fall FM?........	1	2	3	7	8

Q714	POUR TOUTE REPONSE (1) OU (2) A Q713, POSEZ LA QUESTION CI-APRES<br>Au cours des 12 derniers mois, avez-vous entendu un thème sur la PF ou l'Espacement des naissances dans cette émission ?	OUI / NON / NSP/PAS SURE	
	a. Emission santé sur Rail Bi FM, Oxyjeunes Afia FM, Mozdair, Jokko rufisque, dounya, Sud FM Renndo, Lamp fall, Mbour FM ou Alfayda ?..........	1 / 2 / 8	
	b. Emission Religieuse sur Rail Bi FM, Oxyjeunes Afia FM, Jokko rufisque, dounya, Sud FM KL, Renndo, Lamp fall, Mbour FM ou Alfayda ?.........	1 / 2 / 8	
	c. Emission musicale sur Rail Bi FM, Oxyjeunes Afia FM, Jokko rufisque, dounya, Sud FM KL Mbour FM ou Alfayda ?...................................	1 / 2 / 8	
	d. Emission interactive Loci Xam sur Sud FM ?........	1 / 2 / 8	
	e. Emission Xam sa yaram sur Lamp fall FM?........	1 / 2 / 8	
Q715	Au cours des 12 derniers mois, avez-vous entendu **un spot/une publicité** qui fait la promotion de la PF ou l'espacement des naissances sur la radio **Sud FM, Zik FM, RFM, Walf FM, Lamp FALL FM ou RSI OU DANS UNE RADIO COMMUNAITAIRE**	OUI........................ 1<br>NON........................ 2<br>N'ECOUTE PAS CES RADIOS........................ 7<br>NSP/PAS SURE........................ 8	
Q716	A votre avis, les messages sur les thèmes suivants à la radio sont-ils acceptables ou non ?	ACCEPTABLE / INACCEPTABLE	
	a. Planification familial/espacement des naissances	a. 1 / 2	
	b. VIH/SIDA	b. 1 / 2	
	c. Santé maternelle (soins prenatals, acouchement,…)	c. 1 / 2	
	d. Santé infantile (vaccination, soins préventifs,…)	d. 1 / 2	
	e. Santé de reproduction (ISTs, infécondité)	e. 1 / 2	
Q717	Regardez-vous la télévision?	OUI........................ 1<br>NON........................ 2	→ Q723
Q718	Combien de jours par semaine regardez-vous la télévision?	NOMBRE DE JOURS PAR SEMAINE........................ [ ]<br>PAS REGULIEREMENT........................ 8	
Q719	En moyenne combien de temps au total regardez-vous la télévision dans un jour normal ?	HEURES PAR JOUR............ ....1 [0][ ][ ]<br>OU<br>MINUTES PAR JOUR............ .....2 [ ][ ][ ]	

Q720	Quelles chaînes regardez-vous généralement à la télévision?<br><br>INSISTER: D'autres chaines de TV?<br><br>ENREGISTER TOUT CE QUI EST MENTIONNE.	RTS 1 .... A<br>WALF TV.......... B<br>RTS2.......... C<br>TFM .... D<br>2STV .... E<br>SENTV .... F<br>LAMP FALL TV .... G<br>RDV.......... H<br>LCS .... I<br>AFRICABLE .... J<br>MOURCHID TV .... K<br>TOUBA TV .... L<br>SALOUM TV .... M<br>TV5.......... N<br>FRANCE24.......... O<br>CANAL HORIZONS .... P<br>AUTRES________________ X<br>(PRECISER)	
Q721	Avez-vous vu des informations sur la PF à la télévision au cours des trois derniers mois ?	OUI .... 1<br>NON 2	 → Q723
Q722	Quelles informations avez-vous vu à la télévision sur la planification familiale?<br><br>INSISTER: D'autres informations?<br><br>ENCERCLER TOUT CE QUI EST MENTIONNE.<br><br>SI PILULE, INSISTER POUR SAVOIR SI PILULE JOURNALIERE OU CONTRACEPTION D'URGENCE.	PILULES.......... A<br>DIU.......... B<br>PRESERVATIFS.......... C<br>INJECTABLES .... D<br>IMPLANT.......... E<br>CONTRACEPTION D'URGENCE.......... F<br>STERILISATION FEMININE.......... G<br>STERILISATION MASCULINE.......... H<br>ALLAITEMENT MATERNEL.......... I<br>METHODE DES JOURS FIXES.......... J<br>AGE DU MARIAGE.......... K<br>RETARDER L'AGE DU 1[ER] RAPPORT SEXUEL.......... L<br>RETARDER LA PREMIERE NAISSANCE.......... M<br>ESPACEMENT DES NAISSANCES/MOYTU NEF.......... N<br>LIMITATION DE LA TAILLE DE LA FAMILLE.......... O<br>BIEN-FONDÉ DE LA PF.......... P<br>COMMUNICATION ENTRE EPOUX.......... Q<br>AUTORITE GOOUVERMENTALE PARLER DE PF.......... R<br>DISCOURS D'UN LEADER RELIGIEUX SUR LA PF.......... S<br>RUMEURS ET CRAINTES SUR LA PF.......... T<br>AUTRES________________ X<br>(PRECISER)	
Q723	Vous arrive-t-il suivre l'emission **Dine Ak Diamano** sur **Walf TV** ?	OUI TOUJOURS/SOUVENT.......... 1<br>OCCASIONNELLEMENT.......... 2<br>NON JAMAIS.......... 3<br>NE SUIVRE PAS CETTE CHAINE 7<br>NE CONNAIT PAS CETTE ÉMISSION 8	 <br>→ Q725<br>→ Q725<br>→ Q725
Q724	Au cours des 12 derniers mois, avez-vous entendu un thème sur la PF/l'Espacement des naissances dans cette émission ?	OUI.......... 1<br>NON.......... 2<br>NSP/PAS SURE.......... 8	
Q725	Vous arrive-t-il suivre l'emission **Ndieguemar** sur **Télévision Futurs Média (TFM)** ?	OUI TOUJOURS/SOUVENT.......... 1<br>OCCASIONNELLEMENT.......... 2<br>NON JAMAIS.......... 3<br>NE SUIVRE PAS CETTE CHAINE 7<br>NE CONNAIT PAS CETTE ÉMISSION 8	 <br>→ Q727<br>→ Q727<br>→ Q727
Q726	Au cours des 12 derniers mois, avez-vous entendu un thème sur la PF/l'Espacement des naissances dans cette émission ?	OUI.......... 1<br>NON.......... 2<br>NSP/PAS SURE.......... 8	
Q727	Vous arrive-t-il suivre l'emission **Li ci penc mi** sur **Télévision Futurs Média (TFM)** ?	OUI TOUJOURS/SOUVENT.......... 1<br>OCCASIONNELLEMENT.......... 2<br>NON JAMAIS.......... 3<br>NE SUIVRE PAS CETTE CHAINE 7<br>NE CONNAIT PAS CETTE ÉMISSION 8	 <br>→ Q729<br>→ Q729<br>→ Q729

Q728	Au cours des 12 derniers mois, avez-vous entendu un thème sur la PF/l'Espacement des naissances dans cette émission ?	OUI....... 1<br>NON....... 2<br>NSP/PAS SURE....... 8	
Q729	Vous arrive-t-il de suivre l'emission **Thow li Thiow li** sur **2STV** ?	OUI TOUJOURS/SOUVENT....... 1<br>OCCASIONNELLEMENT....... 2<br>NON JAMAIS....... 3<br>NE SUIVRE PAS CETTE CHAINE 7<br>NE CONNAIT PAS CETTE ÉMISSION 8	 <br>→ Q731<br>→ Q731<br>→ Q731
Q730	Au cours des 12 derniers mois, avez-vous entendu un thème sur la PF/l'Espacement des naissances dans cette émission ?	OUI....... 1<br>NON....... 2<br>NSP/PAS SURE....... 8	
Q731	Vous arrive-t-il suivre l'emission **Sen DINE** sur la **SEN TV**?	OUI TOUJOURS/SOUVENT....... 1<br>OCCASIONNELLEMENT....... 2<br>NON JAMAIS....... 3<br>NE SUIVRE PAS CETTE CHAINE 7<br>NE CONNAIT PAS CETTE ÉMISSION 8	 <br>→ Q733<br>→ Q733<br>→ Q733
Q732	Au cours des 12 derniers mois, avez-vous entendu un thème sur la PF/l'Espacement des naissances dans cette émission ?	OUI....... 1<br>NON....... 2<br>NSP/PAS SURE....... 8	
Q733	Vous arrive-t-il de suivre l'emission **Decriptage sur la 2STV?**	OUI TOUJOURS/SOUVENT....... 1<br>OCCASIONNELLEMENT....... 2<br>NON JAMAIS....... 3<br>NE SUIVRE PAS CETTE CHAINE 7<br>NE CONNAIT PAS CETTE ÉMISSION 8	 <br>→ Q735<br>→ Q735<br>→ Q735
Q734	Au cours des 12 derniers mois, avez-vous entendu un thème sur la PF/l'Espacement des naissances dans cette émission ?	OUI....... 1<br>NON....... 2<br>NSP/PAS SURE....... 8	
Q735	Vous arrive-t-il de suivre l'emission **Xam sa yaram sur Lamp FALL TV?**	OUI TOUJOURS/SOUVENT....... 1<br>OCCASIONNELLEMENT....... 2<br>NON JAMAIS....... 3<br>NE SUIVRE PAS CETTE CHAINE 7<br>NE CONNAIT PAS CETTE ÉMISSION 8	 <br>→ Q737<br>→ Q737<br>→ Q737
Q736	Au cours des 12 derniers mois, avez-vous entendu un thème sur la PF/l'Espacement des naissances dans cette émission ?	OUI....... 1<br>NON....... 2<br>NSP/PAS SURE....... 8	
Q737	Au cours des 12 derniers mois, avez-vous entendu **un spot/une publicité** qui fait la promotion de la PF ou l'espacement des naissances sur la télévision **TFM, SEN TV, WALF TV, RTS1, RTS2, Lamp FALL TV ou 2STV**	OUI....... 1<br>NON....... 2<br>N'REGARDE PAS CES CHAINES....... 7<br>NSP/PAS SURE....... 8	
Q738	A votre avis, les messages sur les thèmes suivants à la télévision sont-ils acceptables ou non ?	(voir tableau ci-dessous)	
Q739	Avez-vous personnellement un téléphone portable à votre propre usage?	OUI .... 1<br>NON .... 2<br>NE CONNAIT PAS TELEPHONE PORTABLE....... 8	→ Q741<br><br>→ Q744b

Q738:

		ACCEPTABLE	INACCEPTABLE
a. Planification familial/espacement des naissances	a.	1	2
b.VIH/SIDA	b.	1	2
c. Santé maternelle (soins prenatals, acouchement,…)	c.	1	2
d. Santé infantile (vaccination, soins préventifs,…)	d.	1	2
e. Santé de reproduction (ISTs, infécondité)	e.	1	2

Q740	Avez-vous accès au téléphone portable?	OUI ... 1<br>NON ... 2	
Q741	Avez-vous jamais eu une conversation téléphonique sur la PF ?	OUI ... 1<br>NON ... 2	
Q742	Seriez-vous à l'aise en recevant des messages sur la PF /Contraception ou la santé par SMS ?	OUI ... 1<br>NON ... 2<br>NE CONNAIT PAS SMS... 8	 <br>→ Q744
Q743	Au cours des <u>3 derniers mois</u>, avez-vous reçu des messages SMS sur la PF/Contraception ?	OUI ... 1<br>NON ... 2	
Q744	Au cours des <u>3 derniers mois</u>, avez-vous accédé à internet,web, ou email au moins par l'un des moyens suivants :<br>a. Téléphone portable	OUI ... 1<br>NON ... 2<br>NE CONNAIT PAS INTERNET... 8	 <br>→ Q747
	b. Ordinateur	OUI ... 1<br>NON ... 2	
Q745	Au cours des <u>3 derniers mois</u>, avez-vous accédé à internet,web, facebook, ou email au moins une fois?	OUI ... 1<br>NON ... 2<br>NE SAIT PAS ... 8	 → Q747<br>→ Q747
Q746	Avez-vous vu des messages sur la PF à l'internet,web, facebook, ou email au cours des <u>trois derniers mois</u> ?	OUI ... 1<br>NON ... 2<br>NE SAIT PAS ... 8	
Q747	Etes-vous membre d'une association, un groupe, une organisation?	OUI ... 1<br>NON ... 2	 → Q749
Q748	Avez-vous jamais vu ou entendu des informations sur la PF au cours de rencontres de l'organisation?	OUI ... 1<br>NON ... 2<br>NE SAIT PAS ... 8	
Q749	<u>Au cours des 12 derniers mois,</u> avez-vous participe à une **conversation communautaire** animee par un relais/bajenu gokh (ou autre personnel communautaire) où l'on a parlé de PF ou de l'espacement des naissances?	OUI... 1<br>NON... 2<br>NSP/NE SE SOUVIENT PAS... 8	 → Q751<br>→ Q751
Q750	De quoi avait-on parlé ?<br><br>ENCERCLER TOU CE QUI EST MENTIONNE	GESTION DES RUMEURS ET CRAINTES... A<br>IMPLICATION DES HOMMES... B<br>POSITION DE L'ISLAM SUR LA PF... C<br>DISCUSSION AU SEIN DU COUPLE... D<br>GESTION DES EFFETS SECONDAIRES... E<br>BIEN-FONDÉ DE LA PF... F<br>OBSTACLES SOCIO- CULTURELS À L'ORIGINE DE LA FAIBLE ACCEPTATION DE LA PF... G<br>AUTRE ___ X<br>PRECISER	
Q751	<u>Au cours des 12 derniers mois,</u> avez-vous participe à une **niche** animee par un relais/bajenu gokh (ou autre personnel communautaire) où l'on a parlé de PF ou de l'espacement des naissances ?	OUI... 1<br>NON... 2<br>NSP/NE SE SOUVIENT PAS... 8	 → Q753<br>→ Q753

Q752	De quoi avait-on parlé ?<br><br>ENCERCLER TOU CE QUI EST MENTIONNE	GESTION DES RUMEURS ET CRAINTES....... A<br>IMPLICATION DES HOMMES....... B<br>POSITION DE L'ISLAM SUR LA PF....... C<br>DISCUSSION AU SEIN DU COUPLE....... D<br>GESTION DES EFFETS SECONDAIRES....... E<br>BIEN-FONDÉ DE LA PF....... F<br>OBSTACLES SOCIO- CULTURELS À L'ORIGINE DE LA FAIBLE ACCEPTATION DE LA PF....... G<br>AUTRE ______ X<br>PRECISER	
Q753	Au cours des 12 derniers mois, avez-vous recu chez-vous une **visite** d'un relais/bajenu gokh (ou autre personnel communautaire) qui a parlé de PF ou de l'espacement des naissances ?	OUI....... 1<br>NON....... 2<br>NSP/NE SE SOUVIENT PAS....... 8	 → Q755<br>→ Q755
Q754	De quoi avait-il/elle parlé ?<br><br>ENCERCLER TOU CE QUI EST MENTIONNE	GESTION DES RUMEURS ET CRAINTES....... A<br>IMPLICATION DES HOMMES....... B<br>POSITION DE L'ISLAM SUR LA PF....... C<br>DISCUSSION AU SEIN DU COUPLE....... D<br>GESTION DES EFFETS SECONDAIRES....... E<br>BIEN-FONDÉ DE LA PF....... F<br>OBSTACLES SOCIO- CULTURELS À L'ORIGINE DE LA FAIBLE ACCEPTATION DE LA PF....... G<br>AUTRE ______ X<br>PRECISER	
Q755	Au cours des 12 derniers mois, avez-vous participé à une **causerie religieuse** où l'on a parlé de PF/espacement des naissances ?	OUI....... 1<br>NON....... 2<br>NSP/NE SE SOUVIENT PAS....... 8	 → Q757<br>→ Q757
Q756	De quoi avait-on parlé ?<br><br>ENCERCLER TOU CE QUI EST MENTIONNE	GESTION DES RUMEURS ET CRAINTES....... A<br>IMPLICATION DES HOMMES....... B<br>POSITION DE L'ISLAM SUR LA PF....... C<br>DISCUSSION AU SEIN DU COUPLE....... D<br>GESTION DES EFFETS SECONDAIRES....... E<br>BIEN-FONDÉ DE LA PF....... F<br>OBSTACLES SOCIO- CULTURELS À L'ORIGINE DE LA FAIBLE ACCEPTATION DE LA PF....... G<br>AUTRE ______ X<br>PRECISER	
Q757	Au cours des 12 derniers mois, avez-vous participe à une **autre activite publique** (conférence, animation, sensibilisation de masse, etc..) où l'on a parlé de PF/espacement des naissances ?	OUI....... 1<br>NON....... 2<br>NSP/NE SE SOUVIENT PAS....... 8	 → Q759<br>→ Q759
Q758	De quoi avait-on parlé ?<br><br>ENCERCLER TOUT CE QUI EST MENTIONNE	GESTION DES RUMEURS ET CRAINTES....... A<br>IMPLICATION DES HOMMES....... B<br>POSITION DE L'ISLAM SUR LA PF....... C<br>DISCUSSION AU SEIN DU COUPLE....... D<br>GESTION DES EFFETS SECONDAIRES....... E<br>BIEN-FONDÉ DE LA PF....... F<br>OBSTACLES SOCIO- CULTURELS À L'ORIGINE DE LA FAIBLE ACCEPTATION DE LA PF....... G<br>AUTRE ______ X<br>PRECISER	
Q759	Au cours des 12 derniers mois, avez-vous entendu/suivi un **Imam, chef religieux ou prêcheur** parler de la PF ou de l'espacement des naissances ?	OUI....... 1<br>NON....... 2<br>NSP/NE SE SOUVIENT PAS....... 8	 → Q801<br>→ Q801
Q760	Parlait-il en faveur ou contre la PF / l'espacement des naissances ?	EN FAVEUR....... 1<br>CONTRE....... 2<br>NE SAIT PLUS....... 8	

SECTIONS 8: MESURES D'INEGALITE BASEE SUR LE SEXE

Maintenant, je voudrais vous poser des questions concernant la manière dont, à votre avis, les décisions concernant le ménage devraient être prises. S'il vous plaît, rappelez-vous que vous devez être le plus honnête possible et que vos réponses resteront confidentielles ; c'est-à-dire, que personne ne verra vos réponses.

Q801	**VERIFIEZ Q415 & Q416**<br>MARIE OU VIT ACTUELLEMENT AVEC UNE FEMME (Q416=1 OU 2) ☐ ↓<br>PAS EN UNION (Q415=3 OU Q416=3, 4 OU 5) ☐ →		**Q806**
Q802	**VERIFIEZ Q120:**<br>SI L'ENQUÊTEE GAGNE DE L'ARGENT (Q120=1 OU 2) ☐ ↓<br>SI L'ENQUÊTEE NE GAGNE PAS DE L'ARGENT (Q120 = 3 OU 4) OU NE TRAVAILLE PAS DU TOUT (Q115=2) ☐ →		**Q804**
Q803	Qui décide de comment utiliser l'argent que vous gagnez : principalement vous, principalement votre partenaire, ou vous et votre partenaire conjointement ?	REPONDANT……… 1<br>PARTENAIRE (S)……… 2<br>REPONDANT ET PARTENAIRE(S) CONJOINTEMENT……… 3<br>AUTRE__________ 6<br>(A PRECISER)	
Q804	Votre /l'une de vos femmes/partenaires travaillent-elle actuellement ou gagne-t-elle de l'argent ?	OUI……… 1<br>NON……… 2	→ **Q806**
Q805	Qui décide de comment utiliser l'argent que votre/vos femme(s)/partenaires gagne(nt) : principalement vous, principalement votre/vos femme(s), ou vous et votre/ vos femmes conjointement ?	REPONDANT……… 1<br>PARTENAIRE (S)……… 2<br>REPONDANT ET PARTENAIRE(S) CONJOINTEMENT……… 3<br>AUTRE__________ 6<br>(A PRECISER)	

			OUI	NON
Q806	Parfois dans le mariage ou relation amoureuse, l'homme interdit à la femme de faire certaines choses.<br>Interdiseriez-vous à votre femme/partenaire de:	a. Travailler en dehors de la maison ?	1	2
		b. Avoir des visites ?	1	2
		c. Rendre visite à ses amis ?	1	2
		d. Rendre visite à sa famille ?	1	2
		e. Utiliser une methode de contraception?	1	2
		f. Utiliser un téléphone mobile ?	1	2

		MARI	FEMME	ENSEMBLE	NSP/ÇA DEPEND
Q807	Selon vous, dans un couple qui devrait avoir le dernier mot dansles décisions suivantes :				
	a. Effectuer les grandes courses de la maison ?	1	2	3	8
	b. Effectuer les petites courses quotidiennes du ménage ?	1	2	3	8
	c. Décider de quand rendre visite à la famille, aux amis, ou aux parents ?	1	2	3	8
	d. Décider de quand et où chercher des soins médicaux pour votre/ vos femme(s)/ partenaire(s) ?	1	2	3	8

		OUI	NON	NSP
Q808	Souvent, un partenaire est contrarié ou vexé par certaines choses que fait sa femme. A votre avis, est-il justifié qu'un partenaire batte ou frappe sa femme dans les situations suivantes ?			
	a Si elle sort sans le lui dire?	1	2	8
	b.Si elle néglige la maison ou les enfants?	1	2	8
	c.Si elle dispute avec lui?	1	2	8
	d.Si elle refuse d'avoir des rapports sexuels avec lui?	1	2	8
	e. Si elle ne prépare pas bien la nourriture?	1	2	8
	f. Si il la suspecte d'être infidèle?	1	2	8
	g.Si elle refuse d'avoir un autre enfant?	1	2	8

Maintenant, je vais vous lire des déclarations concernant les normes basées sur le sexe et les motivations de fécondité. Pour chaque déclaration, SVP, dites-moi si vous approuvez fortement, approuvez quelque peu, désapprouvez quelque peu ou désapprouvez fortement avec ce qui est dit.					
		APPROUVE FORTEMENT	APPROUVE QUELQUE PEU	DESAPPROUVE QUELQUE PEU	DESAPPROUVE FORTEMENT
Q809	Le mari devrait être celui qui décide si le couple devrait utiliser une méthode d'espacement des naissances/de planification familiale.	4	3	2	1
Q810	Les couples qui pratiquent l'espacement des naissances/la planification familiale ont une meilleure qualité de vie que ceux qui n'en pratiquent pas.	4	3	2	1
Q811	Les maris et les femmes devraient discuter de la contraception/l'espacement des naissances.	4	3	2	1
Q812	Les hommes ne devraient pas permettre à leurs femmes d'utiliser la contraception.	4	3	2	1
Q813	Une femme qui utilise la contraception à l'insu de son mari devrait être punie.	4	3	2	1
Q814	Une femme qui n'a pas d'enfants n'est pas une femme complète.	4	3	2	1
Q815	Un homme qui n'a pas d'enfants n'est pas un homme complet.	4	3	2	1
Q816	C'est bien d'avoir beaucoup d'enfants parce que personne ne sait lequel d'entre eux survivra ou sera riche pour s'occuper de ses parents lorsque ceux-ci seront vieux.	4	3	2	1
Q817	C'est à Dieu seul de décider du nombre d'enfants qu'un couple aura.	4	3	2	1
Q818	Une femme devrait continuer de faire des enfants jusqu'à ce qu'elle ait au moins un garçon.	4	3	2	1
Q819	Une femme devrait continuer de faire des enfants jusqu'à ce qu'elle ait au moins une fille.	4	3	2	1
Q820	L'espacement des naissances aide les parents à mieux prendre soin de leurs enfants.	4	3	2	1
Q821	L'utilisation de l'espacement des naissances est permise seulement dans le souci de la santé de la mère et de l'enfant.	4	3	2	1
Q822	Les maris et les femmes devraient discuter du nombre d'enfants qu'ils veulent avoir.	4	3	2	1

SECTION 9 : MOUVEMENTS MIGRATOIRES			
	Maintenant, je voudrais vous demander depuis combien de temps vous vivez ici et d'où vous venez et à quelle fréquence vous visitez d'autres régions.		
Q901	Ou êtes-vous né? Dans cette ville, une autre zone urbaine ou une zone rurale/village ?	CETTE VILLE 1<br>AUTRE ZONE URBAINE………………………… 2<br>UNE ZONE RURALE/VILLAGE………………… 3<br>A L'ÉTRANGER………………………………… 4	
Q902	Depuis combien de temps vivez-vous de manière continue dans cette ville ?<br><br>SI DE QUELQUES MOIS A 11 MOIS, ENREGISTRER 00-11.<br>AUTREMENT, ENREGISTRER NOMBRE D'ANNÉES.	MOIS……………………………………… 1 [ ][ ]<br>ANNÉES………………………………… 2 [ ][ ]<br>DEPUIS TOUJOURS……………………………. 995<br>VISITEUR…………………………………… 996	 <br>Q904<br>Q905
Q903	Juste avant d'habiter dans cette maison/cet appartement, viviez-vous à Mbao, Guédiawaye, Pikine, Dakar, Mbour, Kaolack, ou ailleurs?<br><br>SI LA REPONSE EST LE NOM D'UNE REGION, DEMANDER S'IL S'AGIT D'UNE ZONE RURALE OU D'UNE ZONE URBAINE.<br>SI LA REPONSE EST KAOLACK OU MBOUR, ENCERCLER LES CODES '06' OU '05' SI SEULEMENT IL S'AGIT DES COMMUNES CORRESPONDANTES.	MBAO…………………………………………… 01<br>GUEDIAWAYE…………………………………02<br>PIKINE…………………………………………… 03<br>DAKAR……………………………………………04<br>MBOUR…………………………………………… 05<br>KAOLACK………………………………………… 06<br>AUTRE VILLE…………………………………… 07<br>ZONE RURALE…………………………………… 08<br>ÉTRANGER………………………………………… 09	
Q904	Depuis JANVIER 2011, avez-vous change de résidence/domicile ?	OUI……………………………………………………1<br>NON……………………………………………………2	
Q905	Depuis que vous avez commencé à vivre ici, avez-vous vécu ailleurs pendant 6 mois ou plus?	OUI……………………………………………………1<br>NON……………………………………………………2	
Q906	Quelle ville/zone urbaine au Sénégal avez-vous le plus visite au cours des 12 derniers mois ?<br><br>MENTIONNER LE NOM DE LA VILLE/ZONE URBAINE, DÉPARTEMENT ET RÉGION	IL A VOYAGÉ L'ANNÉE DERNIÈRE…………1<br>NOM VILLE/ZONE URBAINE________________<br><br>NOM DÉPARTEMENT ________________ [ ][ ]<br>A CODIFIER AU BUREAU<br>NOM RÉGION ______________________ [ ][ ]<br>A CODIFIER AU BUREAU<br>IL N'A PAS VOYAGÉ L'ANNÉE DERNIÈRE…. 2	 <br><br><br><br><br>Q911
Q907	Au cours des 12 derniers mois, à quelle fréquence avez-vous visité [LIEU MENTIONNE A Q906]?	PAR SEMAINE………………………… 1 [ ][ ]<br>PAR MOIS……………………………2 [ ][ ]<br>PAR.AN………………………………… 3 [ ][ ]	
Q908	Combien de temps restez-vous habituellement lorsque vous allez là-bas ?	HEURES……………………………… 1 [ ][ ]<br>JOURS ………………………………2 [ ][ ]<br>SEMAINES…………………………… 3 [ ][ ]<br>MOIS…………………………………… 4 [ ][ ]	
Q909	Au cours de vos visites, vous arrive-t-il de discuter de Contraception ou de l'espacement des naissances avec quelqu'un là-bas?	OUI ……………………………………………1<br>NON ……………………………………………2	

Q910	Au cours de vos visites, vous arrive-t-il de recourir à des services de PF ou d'espacement des naissances là-bas?	OUI .... 1<br>NON 2	
Q911	Arrive-t-il que vos amis ou votre famille d'UNE AUTRE VILLE au Sénégal viennent vous rendre visite?	OUI .... 1<br>NON 2	→ Q914
Q912	Vous arrive-t-il de parler de PF/Contraception lorsqu'ils vous rendent visite?	OUI .... 1<br>NON 2	
Q913	Au cours de leurs visites ici, arrive-t-il que vos visiteurs recourent à des services de PF ou d'espacement des naissances?	OUI .... 1<br>NON .... 2<br>NE SAIT PAS 8	
Q914	Au cours des 12 derniers mois, vous est-il arrivé d'aller dans une zone rurale du Sénégal pour visiter vos parents (famille) ou amis?	OUI .... 1<br>NON 2	→ Q920
Q915	A quelle zone rurale êtes-vous allé le plus souvent pour rendre visite à vos parents ou amis au cours des 12 derniers mois?	NOM VILLAGE/ZONE RURALE ____________<br>NOM DÉPARTEMENT ________ [ ][ ]<br>A CODIFIER AU BUREAU<br>NOM RÉGION ________ [ ][ ]<br>A CODIFIER AU BUREAU	
Q916	Au cours des 12 derniers mois, à quelle fréquence avez-vous visité [LIEU MENTIONNE A Q915]?	PAR SEMAINE........ 1 [ ][ ]<br>PAR MOIS........ 2 [ ][ ]<br>PAR.AN........ 3 [ ][ ]	
Q917	Combien de temps restez-vous habituellement lorsque vous allez là-bas ?	HEURES........ 1 [ ][ ]<br>JOURS .... 2 [ ][ ]<br>SEMAINES........ 3 [ ][ ]<br>MOIS........ 4 [ ][ ]	
Q918	Au cours de vos visites, vous arrive-t-il de discuter de Contraception ou de l'espacement des naissances avec quelqu'un là-bas?	OUI .... 1<br>NON 2	
Q919	Au cours de vos visites, vous arrive-t-il de recourir à des services de PF ou d'espacement des naissances là-bas?	OUI .... 1<br>NON 2	
Q920	Arrive-t-il que vos amis ou votre famille d'une zone rurale viennent vous rendre visite?	OUI .... 1<br>NON 2	→ **FIN**
Q921	Vous arrive-t-il de parler de PF/Contraception lorsqu'ils vous rendent visite?	OUI .... 1<br>NON 2	
Q922	Au cours de leurs visites ici, arrive-t-il que vos visiteurs recourent à des services de PF ou d'espacement des naissances?	OUI .... 1<br>NON .... 2<br>NE SAIT PAS 8	
	**HEURE DE FIN DE L'INTERVIEW**	**HEURE** [ ][ ]<br>**MINUTES** [ ][ ]	

OBSERVATIONS DE L'ENQUÊTEUR
À REMPLIR APRÈS AVOIR TERMINÉ L'INTERVIEW

COMMENTAIRES SUR L'ENQUÊTÉ :

COMMENTAIRES SUR DES QUESTIONS PARTICULIÈRES :

OBSERVATIONS DU CHEF D'ÉQUIPE

NOM DU CHEF D'ÉQUIPE : ____________________ DATE : ____________

OBSERVATIONS DU SUPERVISEUR

NOM DU SUPERVISEUR ________________________________ DATE : __________
