# Supplementary material for: Association of men's exposure to family planning programming and reported discussion with partner and family planning use: The case of urban Senegal
Source: PLoS One. 2018 Sep 25;13(9):e0204049. doi: 10.1371/journal.pone.0204049 (PMC6155530; doi:10.1371/journal.pone.0204049)
Supplement: S1 Supporting Information — (DOCX) [file pone.0204049.s003.docx]

- Ethics approval and consent to participate - All study procedures were approved by the Institutional Review Board at the University of North Carolina at Chapel Hill as well as the Comité National d’Ethique pour la Recherche en Santé through the Ministry of Health in Senegal. All participating men were asked to provide signed consent to participate. Prior to the interviews, the household head agreed that the interviewer can approach eligible males ages 15-17 years to request their participation in the study.
- Availability of data and material – Data from this study and all documentation are available upon request through the MLE Dataverse website at: https://dataverse.unc.edu/dataverse/mle
- Competing interests – The authors declare that they have not competing interests
- Funding – Funding for this project comes from a grant awarded to the Carolina Population Center (CPC) by the Bill & Melinda Gates Foundation, and general support from an award to the CPC (P2C HD050924) at The University of North Carolina at Chapel Hill by the Eunice Kennedy Shriver National Institute of Child Health and Human Development. The contents of this article are solely the responsibility of the authors and do not necessarily represent the official views of the CPC, NIH or the Bill & Melinda Gates Foundation.
- Authors' contributions – ISS conceived of the article; ISS and MC performed the analyses for this article; ISS wrote the initial draft of the article; AG oversaw data collection for endline data in the field and reviewed the final manuscript; LC and DG provided insights into the study design and analysis; All authors reviewed versions of the manuscript and provided substantive and methodological inputs throughout manuscript development. All authors read and approved the final version of the manuscript.
